# Characterization of the FMDV-serotype-O isolates collected during 1962 and 1997 discloses new topotypes, CEY-1 and WCSA-1, and six new lineages

**DOI:** 10.1038/s41598-019-51120-0

**Published:** 2019-10-10

**Authors:** Lahiru Thilanka Ranaweera, Upendra Kumari Wijesundara, Hashan Sri-Madhubashana Jayarathne, Nick Knowles, Jemma Wadsworth, Valerie Mioulet, Jayantha Adikari, Cholani Weebadde, Suneth S. Sooriyapathirana

**Affiliations:** 1 Department of Molecular Biology and Biotechnology, Faculty of Science, University of Peradeniya, Peradeniya, Sri Lanka; 2 The Pirbright Institute, Pirbright, Woking, Surrey, United Kingdom; 3 Department of Animal and Food Sciences, Faculty of Agriculture, Rajarata University of Sri Lanka, Puliyankulama, Anuradhapura, Sri Lanka; 4 Department of Plant, Soil and Microbial Sciences, College of Agriculture and Natural Resources, Michigan State University, East Lansing, MI USA; 5 Postgraduate Institute of Science, University of Peradeniya, Peradeniya, Sri Lanka

**Keywords:** Viral epidemiology, Viral evolution

## Abstract

The genetic diversity of the FMD viruses collected from the outbreaks during the second half of the 20^th^ Century in Sri Lanka was assessed in the present study. We sequenced the *VP1* genomic region of the samples collected during FMDV epidemics caused by serotype O in Sri Lanka during 1962 and 1997. For comparison, we sequenced the *VP1* of the related viral isolates collected from other Asian countries. We analyzed the VP1 sequences of the viral strains using the UPGMA method with uncorrected pairwise distances. Nucleotide divergence (ND) thresholds of 15%–20% and 5%–<15% were used to differentiate topotypes and lineages, respectively. We calibrated the divergence times and lineage-specific substitution rates using Bayesian-skyline models. Based on the ND estimations and phylogenetic relationships, we identified and named two new topotypes [CEYLON 1 (CEY-1) and WEST, CENTRAL AND SOUTH ASIA 1 (WCSA-1)] and six new lineages (Syr-62, Srl-77, Tur-69, May-78, Tai-87 and Bur-77) of serotype O. We believe that the novel topotypes and lineages named may have disappeared although they have similar substitution rates for epizootic outbreaks. Because the amino acid selection analysis revealed that the two topotypes and six lineages identified were under purifying selection during the outbreaks.

## Introduction

Foot-and-mouth disease (FMD) is considered as one of the most contagious and acute diseases in ruminants and swine^1,2^. FMD imposes a massive threat to the livestock industry^3,4^. The identification of the correct FMD virus (FMDV) causing a particular FMD incident/epidemic plays a crucial role in taking measures to control and eradicate the disease^5^. There are seven FMDV serotypes (A, C, O, Asia 1, SAT 1, SAT 2, and SAT 3) described according to their antigenic and immunological properties. According to the antigenic and genetic attributes, scientists divided the distribution of FMDV into seven different pools where some serotypes exhibit a tendency to be restricted within specific pools. Among the seven serotypes described, serotype O is the most prevalent and distributed form of FMDV spreading across six pools out of the seven described^4,6^.

Samuel and Knowles, (2001)^1^ divided serotype O into eight geographically and genetically distinct topotypes, viz, MIDDLE EAST-SOUTH ASIA (ME-SA), SOUTHEAST ASIA (SEA), INDONESIA-1 (ISA-2), INDONESIA-2 (ISA-2), CATHAY, WEST AFRICA (WA), EAST AFRICA (EA) and EUROPE-SOUTH AMERICA (Euro-SA). Later EA was renamed EA-1 when three further East African topotypes (EA-2, EA-3, and EA- 4) were described^7^. The topotype and lineage level differentiation is mainly carried out using Nucleotide Divergence (ND) of the VP1 genomic region of FMDV genome^1^. The topotypes and lineages are defined at the VP1 nucleotide identity (NI) thresholds of 80–85% and 90–95%, respectively^1,4,8^.

The VP1 structural protein together with proteins VP2, VP3, and VP4 form the capsid of FMDV. The VP1 capsid protein consists of 211 amino acids (coded by 633 nucleotides). The genotyping, molecular epidemiology and evolutionary studies and development of vaccines against FMDV are widely carried out using the coding region of the VP1 capsid protein due to its high variability among different topotypes and lineages^1,5,9–12^. The highest variability within VP1 genomic region among different topotypes and lineages is located in the critical antigenic sites of VP1 ranging from 140–160 (G-H loop) and 200–213 (C-terminus region)^13^. The G-H loop contains an RGD tripeptide which mediates host cell binding of FMDV via an integrin host cell receptor^14^. Due to the high variability (mutation by substitutions, and insertions and deletions)^15–17^, the serotype specificity and the importance in host cell binding^18^, the VP1 genomic region is widely used for genotyping. The VP1 genomic region is also used to understand the patterns of distributions and molecular phylogenetics of FMDV^6^.

FMD was first reported in Sri Lanka in the mid-19th century^19,20^. Although numerous outbreaks occurred since then, most of the causative strains were not identified. Abeyratne *et al*.^21^ characterized the viral isolates collected from FMD outbreaks from 1997–2014 and introduced a novel endemic lineage, Srl-97, present in Sri Lanka. However, no study has systematically described the outbreaks and the reported cases before 1997 in Sri Lanka leaving a considerable knowledge gap in FMDV evolutionary dynamics within the country. We believe that some of the causative strains could be new to the FMD research community and it is essential to characterize past outbreaks and report the novel lineages and topotypes even if they have become extinct. Thus we have sequenced the VP1 region of the FMDV samples isolated during 1962–1990 in Sri Lanka. For comparison purposes, we carried out VP1 sequencing of the viral isolates which might be related to the FMDV strains of Sri Lanka. Thereby, in the present study, we introduce novel topotypes and lineages of serotype O through detailed molecular phylogenetic and deduced amino acid (AA) analyses.

## Materials and Methods

### Reverse transcription PCR (RT-PCR) and sequencing

Vesicular epithelium samples collected by the veterinary authorities from infected animals in Sri Lanka (n = 22) and Southeast Asian and Middle Eastern countries (n = 34) during 1962–1990 were submitted to the FAO World Reference Laboratory for FMD at Pirbright. Viruses were isolated on primary bovine thyroid cells or on the IB-RS-2 pig kidney cell line (explained in Mao *et al*.^22^ and Chapman and Ramshaw^23^ respectively). Total RNA was extracted from virus isolates using the RNeasy kit (Qiagen, Crawley, West Sussex, UK) as described in Knowles *et al*.^8^. To amplify the VP1 region of the FMDV genome two one-step RT-PCRs were performed using serotype O specific forward primers, *O-1C244F* and *O-1C272F* each with the reverse primer, *EUR-2B52R* using conditions previously described^8^ (Knowles *et al*., 2016). Sequencing of each amplicon was performed using the BigDye® Terminator v3.1 Cycle Sequencing kit and run on an ABI 3730 DNA Analyzer. The VP1 sequences generated in this study are deposited in NCBI, GenBank repository (Table S1). The Pirbright Institute where all the viral experiments were conducted is a United Kingdom Accreditation Service (UKAS) Laboratory. The testing and analysis were conducted according to the strictest bio-containment and safety procedures given in https://www.pirbright.ac.uk/node/37681#panel-9334.

### Phylogenetic analysis

For the differentiation of the FMDV topotypes, we created an alignment using ClustalW algorithm^24^ with all the identified topotypes of serotype O in MEGA 7^25^. We manually checked the total alignment for the addition of unnecessary gaps and interference to the reading frame. As the high amount of missing data can delude the analytical power, we excluded the sequences having less than 500 nucleotides to increase the robustness of the analysis. We analyzed the alignment using Unweighted Pair Group Method with Arithmetic Mean (UPGMA) and uncorrected pairwise distance methods. The tree was then plotted using FigTree v1.4.3^26^.

We subjected the data matrix of the alignment to a model selection analysis to find out the best fit model. We implemented Akaike Information Criterion (AIC)^27^, Bayesian Information Criterion (BIC) and Decision Theory (DT) in jModel Test 2.1.10^28^ for the model selection using CIPRES Science Portal^29^. A rapid boostrap algorithm^30^, using 1000 pseudo-replicates, was implemented using the RAxML software^31^. The best ML tree was selected using – (minus) log-likelihood values, and we imprinted bootstrap values to the tree topology given in the best ML tree.

### Molecular dating to infer divergence times

The nucleotide alignment file was imported into the BEAUti 2 software to create an XML file. The GTR site model^32^ with nucleotide substitution parameters given in the model selection in jModel Test was used. We set the model to the relaxed lognormal clock by considering the substitution rates along each branch. Since we dealt with several topotypes and lineages which have different diversification rates, we used the uncorrelated relaxed lognormal clock as it allows the formation of each clade using one lognormal distribution^33^. We used the software BEAST 2 (2.4.8)^34^ to create the divergence time tree. BEAST allows utilizing a range of stochastic branching models and permits to use the best model that fits the specific data set generated in the present study. Since some of the lineages of the virus that we sequenced could have gone extinct and no longer detected, it is more reliable to use mechanical species level process when drawing the tree topology. We used birth-death process which accounts for both speciation as well as extinction^35^. Since we used the time-stamped sequences to infer the divergence time, we used Birth Death Skyline Serial model as the branching model^36^. We kept the reproductive number (R0) to its default value and maximum boundary up to 10 as the rate of spreading of an epidemic is rarely above 10. Usually, FMD hosts are known to gain immunity in one week after the infection, thus we set infectious-rate-prior to 52 per year^37^. The RNA viruses have a mutation rate of 0.001 substitutions per year per site. Thus we used the mean clock rate of 1 × 10^−3^ with a lognormal distribution. We ran 50 million cycles of Markov Chain Monte Carlos (MCMC) chains to sample from the posterior distribution. We assessed the independent sampling and the chain performance using Effective Sample Size (ESS) and Auto-Correlation time by analyzing the log file in TRACER^38^. We used all the sampled trees used to draw the Maximum Clade Credibility (MCC) tree in the software TreeAnotater. We carried out all the analyses in CIPRES Science Gateway^29^ that provide access to most of the tools used. The time tree was plotted using FigTree v1.4.3^26^.

### Nucleotide selection analysis

We checked the evolutionary signatures on the VP1 genomic region of the novel lineages and topotypes identified in this study using Datamonkey 2.0 platform^39,40^. We used the Fixed Effective Likelihood (FEL) to test the codon-specific selective pressure^41^. We implemented a method known as the Fast, Unconstrained Bayesian Approximation for Inferring Selection (FUBAR)^42^ to compare the FEL result. We performed five MCMC chains for two million generations. We kept the burn-in to 20% while sampling 100 trees from each chain cycle. The selective pressure predictions can be tested in both ML and Bayesian frameworks thus the robustness of our results can be increased. We executed the adaptive Branch Site Random Effective Likelihood model (aBSREL)^43,44^ to check the clade-specific evolution and episodic diversification of newly identified topotypes and lineages.

### Amino acid residue selection analysis

The Directional Evolution of Protein Sequences (DEPS) analysis was performed for the deduced amino acid sequences of the viral isolates in the topotypes CEY-I vs. ISA-II and WCSA-II vs. EA and WA in Datamonkey platform^40^. Initially, two sequence alignments were prepared for the novel topotypes CEY-I and WCSA-II and their sister lineages. The automatic model selection was conducted before the DEPS analysis, as described in Kosakovsky Pond *et al*.^45^. The General Discrete Model was used to calculate the site-to-site rate variation.

## Results

### Phylogenetic analysis

**Figure 1:**
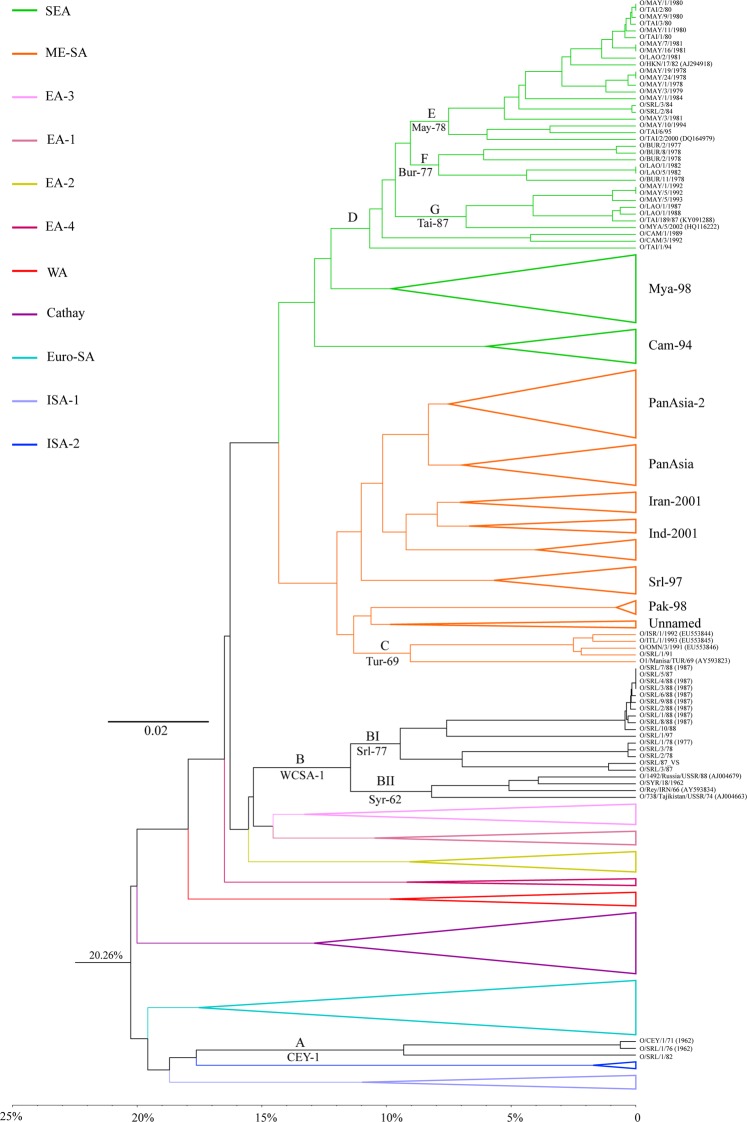
The midpoint rooted distance tree built using UPGMA algorithm. The X-axis represents the nucleotide divergence (ND) calculated using uncorrected pair-wise distances. The colored lines except for the black ones within the tree indicate the known topotypes as depicted in the legend. The collapsed branches represent already known topotypes and lineages. The expanded branches show novel lineages and novel topotypes along with their operational taxonomic units (OTUs). A→G indicate newly formed clades in the tree. The newly assigned names are given below the clades. A and B: Newly named topotypes CEY-1 and WCSA-1, respectively; BI, BII, C, E, F and G: Newly named lineages; BI: Srl-77; BII: Syr-62; C: Tur-69; D: the clade containing three newly named lineages (E: May-78, F: Bur-77 and G: Tai-87) within the topotype SEA.

**Figure 2:**
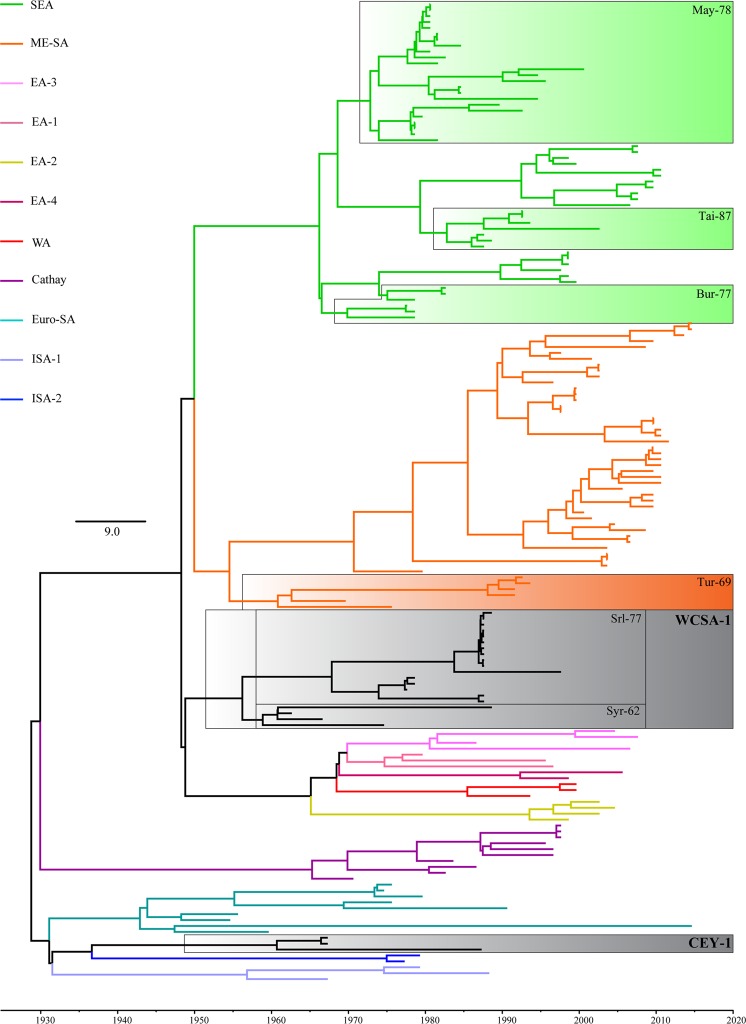
The midpoint rooted MCC tree showing the divergence times of the topotypes and lineages in serotype O. The X-axis represents the time scale. The scale bar indicates the rate of substitutions per site per year. The colored lines except for the black ones within the tree indicate the known topotypes as depicted in the legend. The shaded boxes indicate the novel topotypes and the novel lineages. The exact midpoint rooted MCC tree showing the divergence times with tip labels, node support (Posterior Probabilities and bootstrap values) and 95% Highest Posterior Density node bars is given as Fig. S1.

The topotypes and lineages were resolved at major nodes of the distance tree (Fig. 1). The best fit model with highest AIC value (0.733) for our dataset was GTR + I + G. The substitution rates for the model were calculated as AC = 1.112; AG = 7.827; AT = 1.236; CG = 0.506; CT = 10.392; and GT = 1.0. The proportion of invariance was 0.3183, and the gamma shape parameter was 0.85. Both BIC and DT criteria also gave the similar model in the model selection. We used the parameters of the best-selected model for Bayesian tree construction. The MCMC chains converged maximally after 500,000 cycles and first 10% of the trees sampled were discarded as the burn-in. The ESS for all the priors were above 200 indicating higher robustness in our analysis. It is important to run MCMC chains until ESS exceeds 200 thus the poor mixing of the samples can be avoided. The clade structure and the branching pattern of the MCC tree generated was similar to the best ML tree deduced from the ML bootstrap analysis. Thus, the major clades were well supported by both bootstrap and posterior probability values (Fig. S1). The MCC tree exhibits the evolution of FMD serotype O with divergence times (Fig. 2).

### Evolutionary signatures in VP1 region

The total predicted AA alignment including newly named lineages and topotypes, and FMDV serotype O VP1 reference sequence had 46 Parsimony Informative (PI) sites out of 214 positions considered. There was seven PI amino acids (AA) in G-H loop and two in C-terminus region. To infer the evolutionary aspects of newly identified clades, we primarily used ML-based methods given in Datamonkey platform. The evolution of positive (adaptive) or negative (purifying) selection was measured based on the synonymous (dS) and non-synonymous (dN) substitution rates. The FEL analysis calculates the site (codon) specific dS and dN rates in a given alignment based on likelihood ratios. The FUBAR, however, estimates the dS and dN ratios based on the posterior probabilities (>90% pp are given as positively selected codons). The FEL and FUBAR analyses inferred that all the codon positions of the studied viral isolates are under the PURIFYING selection. We have provided the codon-specific rates, likelihood ratios, p values, and pp values in the Table S2. According to the aBSREL analysis, the phylogeny did not support any episodic diversification along each clade.

### Topotype and lineage characterization

The UPGMA tree separated the 55 isolates studied into four major clades that were not reported before (Fig. 1). The distance tree built using UPGMA algorithm had topotype separation at a nucleotide similarity level of 80%–85%.

We identified the separation of topotypes and lineages at major nodes in the midpoint rooted distance tree (Fig. 1). The Clade A consists of FMDV VP1 sequences of the isolates collected in Sri Lanka during 1962–1982. The Clade A separated in the UPGMA tree with an ND of 17.76% indicating the existence of a novel topotype. We named this novel topotype as ‘CEYLON-1 (CEY-1)’ due to its apparent geographical restriction within Sri Lanka. CEY-1 cladded sister to the topotype, ISA-2. We identified two unique AA substitutions within CEY-1 at the positions of 140 and 186 (T140H and T186V) from which T140H is located within the G-H loop and T186V is located within the B-C loop of the VP1 protein. CEY-1 was distantly related to the extinct Indonesian topotypes ISA-1 and ISA-2. The topotypes, CEY-1, ISA-1, and ISA-2, shared the ‘insertion’ of an AA in the B-C loop of VP1 where CEY-1 contained E/Q insertion and ISA-1 and ISA-2 contained either A/T insertion. According to the divergent time analysis, the topotype CEY-1 had diverged from ISA-1 in the year 1936 [95% highest posterior density (HPD) = 66.0308–85.676] (Fig. 2). We estimated the topotype specific substitution rate of CEY-1 as 8.1 × 10^−3^ substitutions per site per year.

The Clade B nested monophyletic to the African topotypes, EA-3 and EA-1 (Fig. 1). The FMDV sequences isolated in Eurasian and Middle East countries represent the Clade B (Table S1). The clade B exceeds the ND cut-off of 15%, thus can be treated as a novel topotype and we named it as ‘WEST, CENTRAL AND SOUTH ASIA 1’ (WCSA-1). The MCC time tree showed that the Time for The Most Recent Common Ancestor (TMRCA) was 70 years ago in 1955 (95% HPD = 59.288–70.333) (Fig. 2). The comparison of the AA sequences of VP1 protein revealed a unique substitution of A by either P, S or T at the 210^th^ position located within the C-terminus. We estimated the topotype specific substitution rate of WCSA-1 as 8.8 × 10^−3^ substitutions per site per year.

The topotype WCSA-1 split at 11.44% of ND into two sister clades, BI and BII indicating the existence of two lineages (Fig. 1). The Clade BI solely comprised of Sri Lankan isolates and the first isolate of B1 was sampled in 1977. Thus we identified Clade BI as a novel lineage and named as ‘Srl-77’. This novel lineage is distinctive by having unique AA substitution of K by either E or V (K43E/V) at the βB sheet and E by either P or T (E47P/T) at B-C loop. The Srl-77 lineage had a prominent AA replacement of T by A (T172A) located within the βI sheet. We estimated the topotype specific substitution rate of Srl-77 as 4.8 × 10^−3^ substitutions per site per year.

The Clade BII, representing the other lineage sister to Srl-77 (Fig. 1), shared multiple geographical localities ranging from Central Asia, South Asia, and the Middle East. We named BII as ‘Syr-62’. The divergence time for the split between Srl-77 and Syr-62 was estimated to be at 63 years ago in 1955 (95% HPD = 54.632–63.454) (Fig. 2). Syr-62 contained two unique mutations which are, substitution of T by either V or D at 172^nd^ position (T172V/D) located within the βI loop and substitution of D by T (D139T) found within the G-H loop. We estimated the topotype specific substitution rate of Syr-62 as 5.9 × 10^−3^ substitutions per site per year.

We identified a novel clade, labeled C, within the ME-SA topotype (Fig. 1). The Clade C separated from rest of the group with an ND of 11.32%. Thus we treated this clade also as a novel lineage. Even though the existence of this lineage is found in multiple regions, we named this lineage as ‘Tur-69’ as this was first detected in Turkey in 1969. Four viral strains including one Sri Lankan isolate represent Tur-69. Among these four isolates, except the one collected from Turkey (O_1_/Manisa/TUR/69; AY593823), substitution of I by A at 57^th^ position (I57A) located within the C-D loop was unique to Tur-69. The deduced AA sequence of Tur-69 further revealed that replacement of A by V (A4V) located within the N-terminus was unique for all the individuals of ME-SA including Tur-69. This lineage has diverged from rest of the ME-SA group around 1965 (95% HPD = 44.404–53.340) (Fig. 2). We estimated the topotype specific substitution rate of Tur-69 as 4.7 × 10^−3^ substitutions per site per year.

The Clade D formed a sister clade with Mya-98 of SEA topotypes separating each other at an ND of 12.22% (Fig. 1). The UPGMA tree divided the Clade D into three sub-clades that exceed 5% ND. The Clades E and F separated at 9.04% of ND. The Clade E consisted of isolates collected from different countries (mostly from Southeast Asia and two isolates from Sri Lanka). Thus we named Clade E as ‘May-78’ a novel lineage under the topotype, SEA. Within the SEA topotype, a unique substitution of A by T (A13T) at N-terminus was prominent in May-78. We estimated the topotype specific substitution rate of May-78 as 4.5 × 10^−3^ substitutions per site per year.

The Clade F consisted of the viral isolates collected from Burma and Laos. The first isolate of Clade F was reported in Burma (now named Myanmar) in 1977. We identified Clade F as a novel lineage and assigned the name ‘Bur-77’. This lineage had two fixed AA substitutions of T by either L or P (T142L/P) and N by either A or T (N143A/T) located within the G-H loop. Bur-77 shared T142L/P and N143A/T with Cam-94 in the deduced VP1 capsid protein chain. Within the Clade D, May-78 and Bur-77 separated from the Clade G at an ND of 9.64%. The Clade G exclusively comprised of sequences obtained from the isolates collected in Malaysia, Thailand, and Laos in the years between 1987 and 2002. Since the first isolate was collected from Laos in 1987, we named the Clade G as ‘Tai-87’. The deduced AA sequence of VP1 region revealed prominent AA substitution of Q by H located within the N-terminus (Q28H) which was shared with the lineage, Mya-98. We identified a unique AA substitution of E by A present in Tai-87, situated within the E-F loop (E102A). Moreover, some individuals of Tai-87 showed a unique AA substitution of K by Q (K41Q) located within the αZ helix.

We used the clustering pattern of the distance tree to identify the topotypes and lineages based on the ND (Fig. 1) as described previously^1,46,47^. However, the MCC and ML trees had different topology within the topotype SEA. A well supported clade (bs = 94 and PP = 96%) was formed including May-78 individuals (Fig. S1). All the individuals (O/MYA/1/92, O/MYA/5/92, O/MYA/5/93, O/MYA/5/2002, O/LAO/1/87, O/LAO/1/88, O/TAI/189/87) of Tai-87 lineage in the UPGMA tree cladded sister to Mya-98 in both ML and MCC trees (bs = 98, PP = 98) (Fig. S1). The TMRCA among Mya-98, Tai-87, and May-78 was estimated as the year 1967 (95% HDP = 40.534–48.376) (Fig. 2). Moreover, Cam-94^48^ also formed a sister clade with the all the individuals of Bur-77 lineage (O/LAO/1/82, O/LAO/5/82, O/BUR/11/78, O/BUR/2/77, O/BUR/8/78, O/BUR/2/78) in both ML and MCC trees. Tai-87 estimated to be originated in 1965 (95% HPD = 43.1129–51.539) in the calibrated time tree (Fig. 2). The newly named two topotypes and six lineages along with the estimated year of origin and evolutionary parameters are summarized in Table 1.

**Table 1:** Summary of newly identified clades (topotypes and lineages).

Clade	Estimated year of origin	95% height HPD^#^	Evolutionary rate^*^	Specific mutations in VP1 protein sequence	No. of PI^$^ sites
**Topotype**					
CEY-1	1936	66.031–85.676	8.10 × 10^−3^	E/Q insertion, T140H, T186V	20
WCSA-1	1955	54.632–63.454	8.82 × 10^−3^	A210S/P/T	120
**Lineage** (**topotype**)					
Syr-62 (WCSA-1)	1962	53.653–59.840	5.92 × 10^−3^	D139T, T172D/V	29
Srl-77 (WCSA-1)	1967	41.472–52.618	4.84 × 10^−3^	K43E/V	88
Tur-69 (ME-SA)	1960	51.697–63.540	4.74 × 10^−3^	I57A	32
May-78 (SEA)	1972	43.112–51.539	4.45 × 10^−3^	A13T	109
Tai-87 (SEA)	1982	27.860–32.314	4.99 × 10^−3^	E102A, K41Q	40
Bur-77 (SEA)	1965	43.113–51.538	3.93 × 10^−3^	—	60

^#^Highest posterior density.

^*^Evolutionary rate is given in Substitutions per site per year.

^$^Parsimony informative site.

### Directional selection of amino acid residues

The DEPS analyses revealed that there was one directionally evolving residue when CEY-1 is compared with ISA-2 (*P* = 0.0013). There were eight directionally evolving residues when the topotype WCSA-1 is compared with EA and WA topotypes (0.0 ≤ *P* ≤ 0.0008) (Table 2). The two sites undergoing directional selection between CEY-1 and ISA-2 appeared as convergent evolution or repeated substitution. There were 19 sites undergoing directional selection between WCSA-1 vs. EA and WA. Only one site out of 19 was undergoing frequency-dependent selection. Whereas 14 sites were undergoing the selection kind of convergent evolution or repeated substitution. The remaining four sites were undergoing convergent evolution / repeated substitution or frequency-dependent selection (Table 3).

**Table 2:** Selected residue report of the DEPS analyses.

Residue	*P*-value	Bias term	Proportion of affected sites	Directionally evolving sites^*^
**CEY-1 vs**. **ISA-2**				
V	0.0013	19.68	12.78%	2
**WCSA-1 vs**. **EA and WA topotypes**				
A	0.0001	14.69	3.40%	3
D	0.0000	14.25	11.61%	3
G	0.0000	15.56	9.07%	2
I	0.0001	29.53	5.48%	3
R	0.0002	27.69	4.03%	3
S	0.0000	8.35	10.41%	5
T	0.0000	5.78	12.44%	5
V	0.0008	3.48	18.15%	1

^*^The details of the sites are given in Table 3.

**Table 3:** Sites undergone directional selection revealed in DEPS analyses.

Site	Composition	Root	Inferred Substitutions	DEPS EBF	Selection kind
**CEY-1 vs**. **ISA-2**					
145	V_2_G_1_E_1_	G	E_1_ ↔ _0_G, G_0_ ↔ _2_V	V: 181.7	V: CES/RSS
174	V_3_L_1_	L	L_0_ ↔ _3_V	V: 1084.5	V: CES/RSS
**WCSA-1 vs**. **EA and WA topotypes**					
4	P_17_S_6_L_3_Q_1_	P	L_3_ ↔ _0_P, P_0_ ↔ _1_Q, P_0_ ↔ _6_S	S: 202.1	S: CES/RSS
21	E_23_A_4_V_1_	E	A_4_ ↔ _0_E, E_0_ ↔ _1_V	A: 113.5	A: CES/RSS
57	T_14_I_13_A_1_	I	A_1_ ↔ _0_I, I_0_ ↔ _14_T	I: >10^5^ T: >10^5^	I: FDSS T: CES/RSS
69	T_19_A_9_	A	A_0_ ↔ _19_T	T: 1140.4	T: CES/RSS
70	A_22_S_5_	A	A_0_ ↔ _5_S	A: >10^5^ S: >10^5^	A: FDSS S: CES/RSS
77	L_26_I_2_	L	I_2_ ↔ _0_L	I: 362.6	I: CES/RSS
86	N_24_D_3_E_1_	N	D_3_ ↔ _0_N, E_1_ ↔ _0_N	D: 617.2	D: CES/RSS
97	S_10_A_9_T_9_	A	A_9_ ↔ _0_S, A_0_ ↔ _1_T, S_0_ ↔ _8_T	S: 2014.9	S: FDSS
130	V_25_A_3_	V	A_3_ ↔ _0_V	A: 472.2	A: CES/RSS
134	N_24_S_3_E_1_	N	E_1_ ↔ _0_N, N_0_ ↔ _3_S	S: 293.9	S: CES/RSS
136	K_21_R_7_	K	K_0_ ↔ _7_R	R: 7664.2	R: CES/RSS
139	D_7_G_6_T_6_A_3_E_2_V_2_R_2_	D	A_3_ ↔ _0_D, A_0_ ↔ _1_G, A_0_ ↔ _1_T, D_0_ ↔ _2_E, D_0_ ↔ _5_G, D_0_ ↔ _2_R, D_0_ ↔ _5_T, D_0_ ↔ _2_V	D: 6454.0 G: >10^5^ T: 1011.1	D: FDSS G: CES/RSS T: CES/RSS
140	G_12_T_7_A_7_S_1_V_1_	G	A_7_ ↔ _0_G, G_0_ ↔ _1_S, G_0_ ↔ _7_T, G_0_ ↔ _1_V	G: 4010.7 T: 247.6	G: FDSS T: CES/RSS
154	Q_25_R_3_	Q	Q_0_ ↔ _3_R	R: 2552.6	R: CES/RSS
155	K_25_R_3_	K	K_0_ ↔ _3_R	R: 284.6	R: CES/RSS
159	P_12_T_11_A_2_M_2_S_1_	P	A_2_ ↔ _0_P, M_2_ ↔ _0_P, P_0_ ↔ _1_S, P_0_ ↔ _11_T	T: >10^5^	T: CES/RSS
175	T_25_I_2_V_1_	T	I_2_ ↔ _0_T, T_0_ ↔ _1_V	I: 432.1	I: CES/RSS
198	S_20_D_5_T_2_N_1_	N	D_5_ ↔ _1_S, N_1_ ↔ _0_S, S_0_ ↔ _2_T	D: 686.0 S: 661.9	D: CES/RSS S: FDSS
210	V_9_A_9_S_4_E_2_T_2_I_1_P_1_	A	A_0_ ↔ _2_E, A_0_ ↔ _1_I, A_0_ ↔ _1_P, A_0_ ↔ _4_S A_0_ ↔ _2_T, A_0_ ↔ _8_V, S_0_ ↔ _1_V	V: 2926.5	V: CES/RSS

CES: Convergent Evolution Site; RSS: Repeated Substitution Site; FDSS: Frequency Dependent Selection Site

## Discussion

In the present study, the FMDV isolates collected during 1962–1992 nested into four major distinct clades in both UPGMA and MCC trees (Figs 1 and 2). We used a data set containing all the described topotypes of serotype O to identify the phylogenetic positions of the FMDV strains assessed in the present study (Table S1). We used 80–85% NI level for topotype differentiation^1,2,49^. Since the newly identifying lineages could be replaced or extinct, we used 85%-95% NI to distinguish the lineages. A summary of newly named lineages and topotypes are given in Table 1.

The introduction of novel topotypes and lineages are essential when it comes to identification of the viral type of an FMD epidemic. The molecular characterization of FMDV is vital in taking the counter measures to control a disease spreading situation. The typing of the old viral strains allows us to understand and predict the viral evolution which could be extended to present context. The emergence of topotype and lineages to their geographic origin may help to understand the evolutionary process as well as the history of the disease distribution. Although all the topotypes and lineages we introduce in this study are extinct and no longer detected, it solidifies the platform of molecular dating in the context of sampling of ‘old topotypes/lineages.’ Thus, we believe it is necessary to report and characterize the old topotypes and lineages to the scientific community.

The topotype ISA-1 includes FMDV isolates collected in 1962, 1974 and 1983 (ISA/1/62, ISA/8/83, ISA/9/74), while the two sequences of topotype ISA-2 were from isolates collected during 1972 and 1974 (JAV/5/72, ISA/1/74). Although the common ancestry of ISA-1 and ISA-2 was ambiguous in previous studies^4^, these topotypes were thought to be distantly related since they shared only one insertion of a single AA between residues 45 and 46 of the B-C loop. Remarkably, the newly identified topotype CEY-1 had similar insertion and cladded with ISA-2 with higher node supports (bs = 63, PP = 92). The present analysis confirmed that FMD was introduced into Sri Lanka before 1950. CEY-1 had also started to evolve before 1950. According to our MCC tree (Fig. 2), the topotypes ISA-1, ISA-2 and CEY-1 shared monophyly with EURO-SA (bs = 97 and PP = 97), and TMRCA is around 1931 (95% HPD = 72.868–89.331). We believe that ISA-1 viruses may have originated from the introduction of FMD into Indonesia from Holland during the end of the Dutch colony there around 1930.

In Sri Lanka, a massive epizootic FMD outbreak was reported in 1987 where 85,641 cases were reported. Although the FMD researchers identified the responsible serotype of 1987 outbreak, they did not know the exact topotype or the origin of the causative strain. In the present study, we revealed that the causative viral of the FMD outbreak in 1987 was lineage Srl-77 belonged to the newly named topotype WCSA-1. The sister lineage (Syr-62) of Srl-77 shares the isolates in multiple geographic locations. A clade within Syr-62 comprised of three isolates namely O/1492/Russia/USSR/88, O/SYR/18/62 and O/Rey/IRN/66 which shared the most common recent ancestor with a strain collected from Tajikistan (O/738/Tajikistan/USSR/74). Since the Syr-62 lineage contains sequences of the oldest samples collected, we believe WCSA-1 topotype may have a Central Asian origin where it could have spread through the Middle East. The WCSA-1 topotype shares a monophyletic relationship with the clade containing topotypes EA-1, EA-2, EA-3, EA-4, and WA. They all share an AA substitution of A by either S, P or L in N-terminus (at 4th position). The previous understanding was the origin of WA topotype might have evolved from ME-SA^11^. Moreover, many studies on EA topotypes did not reveal the possible origin^7,50^. The recent WRLFMD reports^51–53^ shows that the WA topotype also clustered inside EA topotypes, which is somewhat congruent with the analysis reported in the present study. We believe that the WCSA-1 topotype may be the missing link between African topotypes and ME-SA. We reveal that the African topotypes were originated 54 years before (95% HPD = 46.236–62.432). The African topotypes share a common ancestor with WCSA-1 before 70 years (95% HPD = 58.288–70.233).

The Tur-69 lineage in ME-SA is the oldest lineage present in MCC tree generated in the present study. This Tur-69 has a Mediterranean origin. From there, Tur-69 could have moved to Asia. In 1991, an epidemic was reported in Sri Lanka. The FMDV isolates collected in 1991 from Sri Lanka, O/SRL/1/91, have not been assigned to any lineage to date. We identified the ancestral lineages to all the existing O/SEA lineages. Our time analysis calculated the oldest TMRCA for SEA topotype to be around 1968 (95% HPD = 40.534–48.376). The lineage Cam-94 within SEA could have evolved from Bur-77 according to our analysis. Similarly, the monophyletic relationship between Mya-98 and Tai-87 implies that Mya-98 may have been evolved from Tai-87 lineage. We believe that both Bur-77 and Tai-87 could have been replaced with Cam-94 and Mya-98, respectively, and these types of lineage turnovers are a common phenomenon in FMDV^50^. Although SEA topotype was known to be endemic to Southeast Asian countries until the end of 1990^54^, we identified spread of O/SEA/May-78 towards South Asia as the FMDV isolates, O/SRL/2/84 and O/SRL/3/84), collected from Sri Lanka cladded under May-78. However, the FMD cases caused by May-78 in Sri Lanka may have gone unnoticed due to the existence of Srl-77 outbreak in 1987.

All the newly identified lineages had rates of diversification closer to or lower than the rates of the known epizootic outbreak. The closer and lower diversifications could be due to smaller sampling size in short period leading to a slight elevation in the rates of evolutions of the topotypes and lineages. However, the selection analysis of the present study exhibits all the lineages are under purifying selection thus we believe that new FMDV strains would have replaced all the topotypes and lineages described in the present study or reached evolutionary static states. The dominant convergent evolution or repeated substitution of the residue-sites between the novel topotypes explained in comparison to the sister groups implies that the development of specific immunity in host organisms may have shaped the evolution of FMDV topotypes.

## Conclusion

We identified and named two novel topotypes, CEY-1 and WCSA-1, and six novel lineages, Syr-62, Srl-77, Tur-69, May-78, Tai-87 and Bur-77 by characterizing the VP1 genomic region of the FMDV isolates collected during 1962–1990. We also described the possible origin events and the dynamics of the topotypes and lineages based on the phylogenetic relationships and deduced protein sequences (Table 1). We found that the FMDV outbreaks in Sri Lanka had different geographic origins. We believe that introduction of genetically diverse viral strains of distinct topotypes may have caused the epizootic outbreaks in Sri Lanka. Moreover, in this study, we reconstructed the phylogeny of the serotype O of FMDV. Thus the present study will help FMD scientific community to understand the disease epidemiology, origin, and viral diversification patterns further.

## Supplementary information

Supplementary Information

## Data Availability

The VP1 sequences generated in this study (n = 56) were submitted to GenBank under MK390887-MK390940, KY091288, and AJ294918. The MCC tree with HPD values and expanded OUT names are given in Supplementary Fig. S1. The data generated during the nucleotide selection analysis is provided in the Supplementary Table S1. The data generated in FUBA and FEL analyses are given in Supplementary Table S2. The deduced amino acid sequences are depicted in the Supplementary Table S3.
