# Supplementary material for: Characterization of the FMDV-serotype-O isolates collected during 1962 and 1997 discloses new topotypes, CEY-1 and WCSA-1, and six new lineages
Source: Sci Rep. 2019 Oct 10;9:14526. doi: 10.1038/s41598-019-51120-0 (PMC6787213; doi:10.1038/s41598-019-51120-0)
Supplement: Supplementary file 1 — Supplementary Information [file 41598_2019_51120_MOESM1_ESM.pdf]

Characterization of the FMDV-serotype-O isolates collected during 1962 and 1997 discloses new topotypes, CEY-1 and WCSA-1, and six new lineages

Lahiru Thilanka Ranaweera[1], Upendra Kumari Wijesundara[1], Hashan Sri-Madhubashana Jayarathne, Nick Knowles[2], Jemma Wadsworth[2], Valerie Mioulet[2], Jayantha Adikari[3], Cholani Weebadde[4], Suneth S. Sooriyapathirana[1,5]*

[1]Department of Molecular Biology and Biotechnology, Faculty of Science, University of Peradeniya, Peradeniya, Sri Lanka.

[2]The Pirbright Institute, Pirbright, Woking, Surrey, United Kingdom.

[3]Department of Animal and Food Sciences, Faculty of Agriculture, Rajarata University of Sri Lanka, Puliyankulama, Anuradhapura, Sri Lanka.

[4]Department of Plant, Soil and Microbial Sciences, College of Agriculture and Natural Resources, Michigan State University, East Lansing, MI, USA.

[5]Postgraduate Institute of Science, University of Peradeniya, Peradeniya, Sri Lanka.

*Corresponding author
sunethuop@gmail.com (SSS)

Contents

Supplementary Figure S1: The MCC tree with HPD values and expanded OUT names.

Supplementary Table S1: The data generated during the nucleotide selection analysis.

Supplementary Table S2: The data generated in FUBA and FEL analyses.

Supplementary Table S3: The deduced amino acid sequences.

Supplementary Figure S1: The MCC tree with HPD values and expanded OUT names.

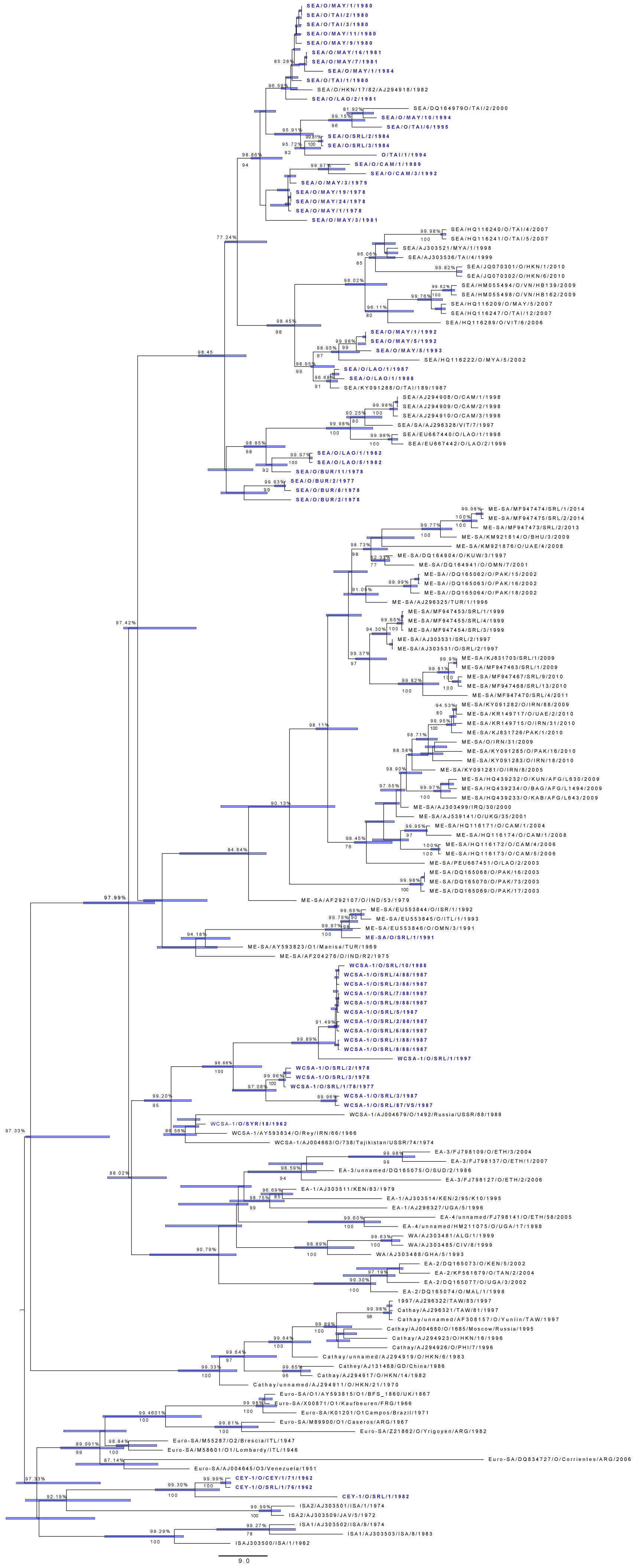
SEA/O/MAY/1/1980
SEA/O/TAI/2/1980
SEA/O/TAI/3/1980
SEA/O/MAY/11/1980
SEA/O/MAY/9/1980
SEA/O/MAY/16/1981
SEA/O/MAY/7/1981
SEA/O/MAY/1/1984
SEA/O/TAI/1/1980
SEA/O/HKN/17/82/AJ294918/1982
SEA/O/LAO/2/1981
SEA/DQ164979O/TAI/2/2000
SEA/O/MAY/10/1994
SEA/O/TAI/6/1995
SEA/O/SRL/2/1984
SEA/O/SRL/3/1984
O/TAI/1/1994
SEA/O/CAM/1/1989
SEA/O/CAM/3/1992
SEA/O/MAY/3/1979
SEA/O/MAY/19/1978
SEA/O/MAY/24/1978
SEA/O/MAY/1/1978
SEA/O/MAY/3/1981
SEA/HQ116240/O/TAI/4/2007
SEA/HQ116241/O/TAI/5/2007
SEA/AJ303521/MYA/1/1998
SEA/AJ303536/TAI/4/1999
SEA/JQ070301/O/HKN/1/2010
SEA/JQ070302/O/HKN/6/2010
SEA/HM055494/O/VN/HB139/2009
SEA/HM055498/O/VN/HB162/2009
SEA/HQ116209/O/MAY/5/2007
SEA/HQ116247/O/TAI/12/2007
SEA/HQ116289/O/VIT/6/2006
SEA/O/MAY/1/1992
SEA/O/MAY/5/1992
SEA/O/MAY/5/1993
SEA/HQ116222/O/MYA/5/2002
SEA/O/LAO/1/1987
SEA/O/LAO/1/1988
SEA/KY091288/O/TAI/189/1987
SEA/AJ294908/O/CAM/1/1998
SEA/AJ294909/O/CAM/2/1998
SEA/AJ294910/O/CAM/3/1998
SEA/SA/AJ296328/VIT/7/1997
SEA/EU667440/O/LAO/1/1998
SEA/EU667442/O/LAO/2/1999
SEA/O/LAO/1/1982
SEA/O/LAO/5/1982
SEA/O/BUR/11/1978
SEA/O/BUR/2/1977
SEA/O/BUR/8/1978
SEA/O/BUR/2/1978
ME-SA/MF947474/SRL/1/2014
ME-SA/MF947475/SRL/2/2014
ME-SA/MF947473/SRL/2/2013
ME-SA/KM921814/O/BHU/3/2009
ME-SA/KM921876/O/UAE/4/2008
ME-SA/DQ164904/O/KUW/3/1997
ME-SA/DQ164941/O/OMN/7/2001
ME-SA//DQ165062/O/PAK/15/2002
ME-SA//DQ165063/O/PAK/16/2002
ME-SA//DQ165064/O/PAK/18/2002
ME-SA/AJ296325/TUR/1/1996
ME-SA/MF947453/SRL/1/1999
ME-SA/MF947455/SRL/4/1999
ME-SA/MF947454/SRL/3/1999
ME-SA/AJ303531/SRL/2/1997
ME-SA/AJ303531/O/SRL/2/1997
ME-SA/KJ831703/SRL/1/2009
ME-SA/MF947463/SRL/1/2009
ME-SA/MF947467/SRL/9/2010
ME-SA/MF947468/SRL/13/2010
ME-SA/MF947470/SRL/4/2011
ME-SA/KY091282/O/IRN/88/2009
ME-SA/KR149717/O/UAE/2/2010
ME-SA/KR149715/O/IRN/31/2010
ME-SA/KJ831726/PAK/1/2010
ME-SA/O/IRN/31/2009
ME-SA/KY091285/O/PAK/16/2010
ME-SA/KY091283/O/IRN/18/2010
ME-SA/KY091281/O/IRN/8/2005
ME-SA/HQ439232/O/KUN/AFG/L630/2009
ME-SA/HQ439234/O/BAG/AFG/L1494/2009
ME-SA/HQ439233/O/KAB/AFG/L643/2009
ME-SA/AJ303499/IRQ/30/2000
ME-SA/AJ539141/O/UKG/35/2001
ME-SA/HQ116171/O/CAM/1/2004
ME-SA/HQ116174/O/CAM/1/2008
ME-SA/HQ116172/O/CAM/4/2006
ME-SA/HQ116173/O/CAM/5/2006
ME-SA/PEU667451/O/LAO/2/2003
ME-SA/DQ165068/O/PAK/16/2003
ME-SA/DQ165070/O/PAK/73/2003
ME-SA/DQ165069/O/PAK/17/2003
ME-SA/AF292107/O/IND/53/1979
ME-SA/EU553844/O/ISR/1/1992
ME-SA/EU553845/O/ITL/1/1993
ME-SA/EU553846/O/OMN/3/1991
ME-SA/O/SRL/1/1991
ME-SA/AY593823/O1/Manisa/TUR/1969
ME-SA/AF204276/O/IND/R2/1975
WCSA-1/O/SRL/10/1988
WCSA-1/O/SRL/4/88/1987
WCSA-1/O/SRL/3/88/1987
WCSA-1/O/SRL/7/88/1987
WCSA-1/O/SRL/9/88/1987
WCSA-1/O/SRL/5/1987
WCSA-1/O/SRL/2/88/1987
WCSA-1/O/SRL/6/88/1987
WCSA-1/O/SRL/1/88/1987
WCSA-1/O/SRL/8/88/1987
WCSA-1/O/SRL/1/1997
WCSA-1/O/SRL/2/1978
WCSA-1/O/SRL/3/1978
WCSA-1/O/SRL/1/78/1977
WCSA-1/O/SRL/3/1987
WCSA-1/O/SRL/87/VS/1987
WCSA-1/AJ004679/O/1492/Russia/USSR/88/1988
WCSA-1/O/SYR/18/1962
WCSA-1/AY593834/O/Rey/IRN/66/1966
WCSA-1/AJ004663/O/738/Tajikistan/USSR/74/1974
EA-3/FJ798109/O/ETH/3/2004
EA-3/FJ798137/O/ETH/1/2007
EA-3/unnamed/DQ165075/O/SUD/2/1986
EA-3/FJ798127/O/ETH/2/2006
EA-1/AJ303511/KEN/83/1979
EA-1/AJ303514/KEN/2/95/K10/1995
EA-1/AJ296327/UGA/5/1996
EA-4/unnamed/FJ798141/O/ETH/58/2005
EA-4/unnamed/HM211075/O/UGA/17/1998
WA/AJ303481/ALG/1/1999
WA/AJ303485/CIV/8/1999
WA/AJ303488/GHA/5/1993
EA-2/DQ165073/O/KEN/5/2002
EA-2/KF561679/O/TAN/2/2004
EA-2/DQ165077/O/UGA/3/2002
EA-2/DQ165074/O/MAL/1/1998
1997/AJ296322/TAW/83/1997
Cathay/AJ296321/TAW/81/1997
Cathay/unnamed/AF308157/O/Yunlin/TAW/1997
Cathay/AJ004680/O/1685/Moscow/Russia/1995
Cathay/AJ294923/O/HKN/16/1996
Cathay/AJ294926/O/PHI/7/1996
Cathay/unnamed/AJ294919/O/HKN/6/1983
Cathey/AJ131468/GD/China/1986
Cathey/AJ294917/O/HKN/14/1982
Cathay/unnamed/AJ294911/O/HKN/21/1970
Euro-SA/O1/AY593815/O1/BFS_1860/UK/1867
Euro-SA/X00871/O1/Kaufbeuren/FRG/1966
Euro-SA/K01201/O1Campos/Brazil/1971
Euro-SA/M89900/O1/Caseros/ARG/1967
Euro-SA/Z21862/O/Yrigoyen/ARG/1982
Euro-SA/M55287/O2/Brescia/ITL/1947
Euro-SA/M58601/O1/Lombardy/ITL/1946
Euro-SA/DQ834727/O/Corrientes/ARG/2006
Euro-SA/AJ004645/O3/Venezuela/1951
CEY-1/O/CEY/1/71/1962
CEY-1/O/SRL/1/76/1962
CEY-1/O/SRL/1/1982
ISA2/AJ303501/ISA/1/1974
ISA2/AJ303509/JAV/5/1972
ISA1/AJ303502/ISA/9/1974
ISA1/AJ303503/ISA/8/1983
ISAJ303500/ISA/1/1962
9.0

Supplementary Table S1: The data generated during the nucleotide selection analysis.

Virus name	Serotype	Topotype	Lineage	Accession	Collected	Species	Location	Country	Reference
GD/CHA/86	O	CATHAY	unnamed	AJ131468	1986	pig	Guangdong, PR	China	unpublished*
HKN/21/70	O	CATHAY	unnamed	AJ294911	3/13/1970	pig	Sheung Shui, New Terrirories, Kowloon	Hong Kong	Knowles *et al* ., 2001
HKN/14/82	O	CATHAY	unnamed	AJ294917	25/02/1982	pig	Hei Ling Chau Island,N.T., Kowloon	Hong Kong	Knowles et al, 2001
HKN/6/83	O	CATHAY	unnamed	AJ294919	12/18/1982	cattle	Hong Kong Island	Hong Kong	Knowles *et al* ., 2001
HKN/16/96	O	CATHAY	unnamed	AJ294923	29/05/1996	pig	TaKwu Ling, Fanling	Hong Kong	Knowles et al, 2001
PHI/7/96	O	CATHAY	unnamed	AJ294926	1996	not known		Philippines	Knowles et al, 2001
N1685/RUS/95Œ	O	CATHAY	unnamed	AJ004680	Jun-95	pig	Moscow	Russian Federation	Beard and Mason, 2000
Yunlin/TAW/97	O	CATHAY	unnamed	AF308157	1997	pig	Yunlin	Taiwan	Beard and Mason, 2000
TAW/81/97	O	CATHAY	unnamed	AJ296321	4/17/1997	pig	I-lan, Taiwan, POC	Taiwan	Samuel and Knowles, 2001
TAW/83/97	O	CATHAY	unnamed	AJ296322	24/40/1997	pig	T'ai-tung, Taiwan, POC	Taiwan	Samuel and Knowles, 2001
SRL/1/82	O	CEY-1	unnamed	MK390919	3/25/1982	cattle (Sinhala)	Lat: 7.500000, Long: 81.666667	Sri Lanka	This study
SRL/1/76	O	CEY-1	unnamed	MK390894	1962	not known		Sri Lanka (Ceylon)	This study
CEY/1/71	O	CEY-1	unnamed	MK390893	12/19/1962	bovine (Scindhe)	Central Livestock Research Station, Polonnaruwa	Sri Lanka (Ceylon)	This study
KEN/2/95	O	EA1	unnamed	AJ303514	1995	not known	Homa Bay	Kenya	Samuel and Knowles, 2001
UGA/5/96	O	EA1	unnamed	AJ296326	17/01/1996	cattle	Mbarara	Uganda	Samuel and Knowles, 2001
K83/79*	O	EA-1	unnamed	AJ303511	1979	not known		Kenya	Samuel and Knowles, 2001
KEN/5/2002	O	EA-2	unnamed	DQ165073	10/9/2002	bovine	Nakuru, Lanet Division	Kenya	unpublished*
MAL/1/98	O	EA-2	unnamed	DQ165074	1998	not known		Malawi	unpublished*
TAN/2/2004	O	EA-2	unnamed	KF561679	2004	bovine	Kibaha District, Pwani Region	Tanzania	Kasanga et al., 2015
UGA/3/2002	O	EA-2	unnamed	DQ165077	2002	not known	Nakasongola district	Uganda	unpublished*
ETH/3/2004	O	EA-3	unnamed	FJ798109	2004	cattle	Shasemene, Oromiya	Ethiopia	Ayelet et al., 2009
ETH/2/2006	O	EA-3	unnamed	FJ798127	May-06	cattle		Ethiopia	Ayelet et al., 2009
ETH/1/2007	O	EA-3	unnamed	FJ798137	2/8/2007	cattle	Ankasha, Agew Awi Zone, Amhara province	Ethiopia	Ayelet et al., 2009
SUD/2/86	O	EA-3	unnamed	DQ165075	11/17/1986	cattle	Omdurman, Khartoum province	Sudan	unpublished
ETH/58/2005	O	EA-4	unnamed	FJ798141	4/20/2005	cattle	Mizan Teferi-Maji	Ethiopia	Ayelet et al., 2009
UGA/17/98	O	EA-4	unnamed	HM211075	1998	not known		Uganda	Ayelet et al., 2009
Campos/BRA/58	O	EURO-SA	O1	K01201	1958	nk	Campos	Brazil	Cheung et al. 1983
Kaufbeuren/FRG/66	O	EURO-SA	O1	X00871	1966	cattle	Kaufbeuren	Germany	Forss et al., 1984
BFS 1860/UK/67	O	EURO-SA	O1	AY593815	1967	cattle	Wallington, Wrexham	United Kingdom	Carrillo et al., 2005
Lombardy/ITL/46	O	EURO-SA	O2	M58601	1946	nk	Lombardy	Italy	Krebs et al., 1991
Brescia/ITL/47	O	EURO-SA	O2	M55287	1947	nk	Brescia	Italy	Krebs et al., 1991
VEN/51	O	EURO-SA	O3	AJ004645	1951	nk		Venezuela	Samuel and Knowles, 2001
Yrigoyen/ARG/82	O	EURO-SA	unnamed	Z21862	1982	nk	Yrigoyen	Argentina	Saiz et al. (1993)
Caseros/ARG/67	O	EURO-SA	unnamed	M89900	1967	cattle	Caseros, Santa Fe	Argentina	Rieder Rojas et al., 1992
Corrientes/ARG/06	O	EURO-SA	unnamed	DQ834727	2006	not known	Corrientes	Argentina	Malirat et al., 2007
ISA/1/62	O	ISA-1	unnamed	AJ303500	1962	not known		Indonesia	Samuel and Knowles, 2001
ISA/8/83	O	ISA-1	unnamed	AJ303503	1983	cattle	East Java	Indonesia	Samuel and Knowles, 2001
ISA/9/74	O	ISA-1	unnamed	AJ303502	10/30/1974	cattle	Tegal Tugu, Gianyar, Bali	Indonesia	Samuel and Knowles, 2001
JAV/5/72	O	ISA-2	unnamed	AJ303509	1972	not known	Java	Indonesia	Samuel and Knowles, 2001
ISA/1/74	O	ISA-2	unnamed	AJ303501	1974	cattle	Bali Quanantine Station	Indonesia	Samuel and Knowles, 2001
BHU/3/2009	O	ME-SA	Ind-2001	KM921813	6/5/2009	cattle	Dorji, Punakha	Bhutan	Knowles et al., 2016a
KUW/3/97	O	ME-SA	Ind-2001	DQ164904	1997	bovine		Kuwait	Knowles *et al* ., 2005
OMN/7/2001	O	ME-SA	Ind-2001	DQ164941	2001	bovine		Oman	Knowles *et al* ., 2005
SRL/2/2013	O	ME-SA	Ind-2001	MF947473	12/26/2013	bovine	Jaffna	Sri Lanka	Abeyratne etal., 2018
SRL/1/2014	O	ME-SA	Ind-2001	MF947474	1/1/2014	bovine	Gampaha	Sri Lanka	Abeyratne etal., 2018
SRL/2/2014	O	ME-SA	Ind-2001	MF947475	1/27/2014	bovine	Puttalam	Sri Lanka	Abeyratne etal., 2018
UAE/4/2008	O	ME-SA	Ind-2001	KM921876	12/30/2008	Gazelle	not known	United Arab Emirates	Knowles et al., 2016
TUR/1/96	O	ME-SA	Iran 2011	AJ296325	31/05/1996	cattle	Kadikoy,Kesan, Edime	Turkey	Samuel and Knowles, 2001
PAK/18/2002	O	ME-SA	Iran-2001	DQ165064	2/27/2002	cattle	Karachi	Pakistan	Schumann et al., 2008
PAK/15/2002	O	ME-SA	Iran-2001	DQ165062	3/15/2002	water buffalo	Sheikhupura, Punjab	Pakistan	Schumann et al., 2008
PAK/16/2002	O	ME-SA	Iran-2001	DQ165063	3/15/2002	bovine	not known	Pakistan	Schumann et al., 2008

Virus name	Serotype	Topotype	Lineage	Accession	Collected	Species	Location	Country	Reference
PAK/16/2003	O	ME-SA	Pak-98	DQ165068	2003	not known	not known	Pakistan	Schumann et al., 2008
PAK/17/2003	O	ME-SA	Pak-98	DQ165069	2003	not known	not known	Pakistan	Schumann et al., 2008
PAK/73/2003	O	ME-SA	Pak-98	DQ165070	10/27/2003	not known	not known	Pakistan	Schumann et al., 2008
CAM/1/2004	O	ME-SA	PanAsia	HQ116171	6/23/2004	cattle	Teuk Phos	Cambodia	Abdul-Hamid *et al* ., 2011
CAM/5/2006	O	ME-SA	PanAsia	HQ116173	6/28/2006	cattle	Angkok Chie, Kampot	Cambodia	Abdul-Hamid *et al* ., 2011
CAM/4/2006	O	ME-SA	PanAsia	HQ116172	7/28/2006	cattle	not known	Cambodia	Abdul-Hamid *et al* ., 2011
CAM/1/2008	O	ME-SA	PanAsia	HQ116174	2/28/2008	cattle	Srang, Kandal	Cambodia	Abdul-Hamid *et al* ., 2011
IRQ/30/2000	O	ME-SA	PanAsia	AJ303499	4/9/2000	cattle		Iraq	Samuel and Knowles, 2001
LAO/2/2006 (2003)	O	ME-SA	PanAsia	EU667451	5/23/2003	cattle		Laos	Khounsy et al., 2009
UKG/35/2001	O	ME-SA	PanAsia	AJ539141	2/20/2001	pig	Brentwood, Essex	United Kingdom	Manson et al., 2003
KAB/AFG/L643/2009	O	ME-SA	PanAsia-2	HQ439233	1/5/2009	cattle	5th, Kabul	Afganistan	Jamal et al., 2011
KUN/AFG/L630/2009	O	ME-SA	PanAsia-2	HQ439232	1/18/2009	cattle	Khanabad, Kunduz	Afganistan	Jamal et al., 2011
BAG/AFG/L1494/2009	O	ME-SA	PanAsia-2	HQ439234	5/18/2009	cattle	Dara-i-Boom, Badgis	Afganistan	Jamal et al., 2011
IRN/8/2005	O	ME-SA	PanAsia-2	KY091281	5/25/2005	sheep	Kamyaran, Kordestan	Iran	Knowles et al., 2016b
IRN/31/2009	O	ME-SA	PanAsia-2	KY091284	4/12/2009	cattle	Khami, Estahban, Fars	Iran	Knowles et al., 2016b
IRN/88/2009	O	ME-SA	PanAsia-2	KY091282	12/21/2009	not known	Esfahan, Esfahan	Iran	Knowles et al., 2016b
IRN/18/2010	O	ME-SA	PanAsia-2	KY091283	2/16/2010	cattle	Nikroz, Mashad, Khorasan Razavi	Iran	Knowles et al., 2016b
IRN/31/2010	O	ME-SA	PanAsia-2	KR149715	2/27/2010	cattle	Laban, Qom	Iran	unpublished*
PAK/1/2010	O	ME-SA	PanAsia-2	KJ831726	2/15/2010	cattle	Karachi	Pakistan	Reeve el al.,2016
PAK/16/2010	O	ME-SA	PanAsia-2	KY091285	3/12/2010	cattle	Sarghoda	Pakistan	Knowles *et al* ., 2016
UAE/2/2010	O	ME-SA	PanAsia-2	KR149717	3/29/2010	gazelle	Al-Ain Wildlife Park & Resort	United Arab Emirates	unpublished*
SRL/1/99	O	ME-SA	Srl-97	MF947453	1999	not known	not known	Sri Lanka	Abeyratne etal., 2018
SRL/3/99	O	ME-SA	Srl-97	MF947454	1999	not known	not known	Sri Lanka	Abeyratne etal., 2018
SRL/4/99	O	ME-SA	Srl-97	MF947455	1999	not known	not known	Sri Lanka	Abeyratne etal., 2018
SRL/2/97	O	ME-SA	Srl-97	AJ303531	1/3/1997	bovine	Old Eluwankulama, Puttalam, North-Western	Sri Lanka	Samuel and Knowles, 2001
SRL/1/2009	O	ME-SA	Srl-97	MF947463	2/18/2009	water buffalo	Thambala, Lankapura Veterinary Range, Polonnaruwa, North Central	Sri Lanka	Abeyratne etal., 2018
SRL/1/2009	O	ME-SA	Srl-97	KJ831703	2/18/2009	water buffalo	Thambala, Lankapura Veterinary Range, Polonnaruwa, North Central	Sri Lanka	Reeve el al.,2016
SRL/9/2010	O	ME-SA	Srl-97	MF947467	4/21/2010	water buffalo	Kandy, Kundasale	Sri Lanka	Abeyratne etal., 2018
SRL/13/2010	O	ME-SA	Srl-97	MF947468	12/2/2010	bovine	Kandy, Kundasale	Sri Lanka	Abeyratne etal., 2018
SRL/4/2011	O	ME-SA	Srl-97	MF947470	5/3/2011	bovine	Trincomalee	Sri Lanka	Abeyratne etal., 2018
ISR/1/92	O	ME-SA	Tur-69	EU553844	5/22/1992	cattle	Tel-Dan	Israel	Valarcher et al., 2008
ITL/1/93	O	ME-SA	Tur-69	EU553845	2/22/1993	cattle	Sarconi, near Viggiano, Potenza	Italy	Valarcher et al., 2008
OMN/3/91	O	ME-SA	Tur-69	EU553846	4/21/1991	cattle	Salalah	Oman	Valarcher et al., 2008
SRL/1/91	O	ME-SA	Tur-69	MK390915	1991	water buffalo		Sri Lanka	This study
O1/Manisa/TUR/69	O	ME-SA	Tur-69	AJ251477	1/4/1969	cattle	Manisa	Turkey	Aktas and Samuel, 2000
IND/R2/75*	O	ME-SA	unnamed	AF204276	1975	not known		India	unpublished*
IND/53/79	O	ME-SA	unnamed	AJ303462	1977	cattle	Tamil Nadu	India	Samuel et al., 1995
HKN/17/82	O	SEA	May-78	AJ294918	3/19/1982	cattle	Pok Wai, San Tin, N.T.	Hong Kong	This study
LAO/2/81	O	SEA	May-78	MK390895	1981	water buffalo	Pakse	Laos	This study
MAY/3/79	O	SEA	May-78	MK390903	1979	not known	not known	Malaysia	This study
MAY/1/78	O	SEA	May-78	MK390900	10/22/1978	cattle	Kg. Kedap - Rantau Panjang, Kelantan	Malaysia	This study
MAY/19/78	O	SEA	May-78	MK390901	11/14/1978	cattle	Buntong, Ipoh, Perak	Malaysia	This study
MAY/24/78	O	SEA	May-78	MK390902	11/24/1978	cattle	Hotan Bulch, Jejawi, Perlis	Malaysia	This study
MAY/1/80	O	SEA	May-78	MK390904	8/19/1980	cattle	Kg. Lachok, Alor Star, Kedah	Malaysia	This study
MAY/9/80	O	SEA	May-78	MK390905	9/10/1980	goat	Daerah Pendang, Kedah	Malaysia	This study
MAY/11/80	O	SEA	May-78	MK390906	9/17/1980	pig	Tanah Liat, BT. Mertajam, P.W.	Malaysia	This study
MAY/3/81	O	SEA	May-78	MK390907	2/7/1981	cattle	Banggol Kenang, Serada, Kuala Trenggnu	Malaysia	This study
MAY/7/81	O	SEA	May-78	MK390908	5/20/1981	cattle	Shah Alam Abatoir, Batutiga, Selangor	Malaysia	This study
MAY/16/81	O	SEA	May-78	MK390909	7/14/1981	cattle	Jalan, Selengor, Petaling Jaya	Malaysia	This study
MAY/1/84	O	SEA	May-78	MK390910	5/23/1984	cattle	Tualang, Batu Gajah, Perak	Malaysia	This study
MAY/10/94	O	SEA	May-78	MK390914	7/8/1994	cattle	Kg. Pintu, Gerbang, Tawang	Malaysia	This study

Virus name	Serotype	Topotype	Lineage	Accession	Collected	Species	Location	Country	Reference
SRL/2/84	O	SEA	May-78	MK390920	8/17/1984	pig	Lat: 7°15' N; Long: 79°50'E	Sri Lanka	This study
SRL/3/84	O	SEA	May-78	MK390921	8/29/1984	cattle	calf experimentally inoculated with 24/84	Sri Lanka	This study
TAI/6/95	O	SEA	May-78	MK390940	1995	not known	not known	Thailand	This study
TAI/1/80	O	SEA	May-78	MK390936	6/19/1980	pig	Nakhonpathom	Thailand	This study
TAI/2/80	O	SEA	May-78	MK390937	12/11/1980	pig	Amphur Muang, Songkla	Thailand	This study
TAI/3/80	O	SEA	May-78	MK390938	12/11/1980	cattle	Amphur Muang, Songkla	Thailand	This study
TAI/2/2000	O	SEA	May-78	DQ164979	1/18/2000	cattle	Songhkla	Thailand	Knowles *et al* ., 2005
LAO/1/82	O	SEA	Bur-77	MK390896	1/23/1982	water buffalo	Sankone district, Savannakhet province	Laos	This study
LAO/5/82	O	SEA	Bur-77	MK390897	1/23/1982	cattle	Vientiane	Laos	This study
BUR/2/78	O	SEA	Bur-77	MK390888	1978	cattle	Kayan	Myanmar (Burma)	This study
BUR/8/78	O	SEA	Bur-77	MK390889	1978	cattle	Kyaunggon	Myanmar (Burma)	This study
BUR/2/77	O	SEA	Bur-77	MK390887	7/9/1977	cattle	Bassein, Kangyidaunt	Myanmar (Burma)	This study
BUR/11/78	O	SEA	Bur-77	MK390890	11/10/1978	cattle	Pyinmabin, 18 M North of Rangoon	Myanmar (Burma)	This study
CAM/1/98	O	SEA	Cam-94	AJ294908	1/5/1998	pig	Khley Taney village, Borset district, Kg. Speu	Cambodia	Knowles *et al* ., 2001
CAM/2/98	O	SEA	Cam-94	AJ294909	1/5/1998	cattle	Kg. Speu	Cambodia	Knowles *et al* ., 2001
CAM/3/98	O	SEA	Cam-94	AJ294910	1/5/1998	cattle	Kg. Speu	Cambodia	Knowles *et al* ., 2001
LAO/1/98	O	SEA	Cam-94	EU667440	Nov-98	cattle	Attapeu province	Laos	Khounsy *et al* ., 2009
LAO/2/99	O	SEA	Cam-94	EU667442	1/6/1999	cattle	Champasak province	Laos	Khounsy *et al* ., 2009
VIT/7/97	O	SEA	Cam-94	AJ296328	8/31/1997	not known	Krong Pa district, Gia Lai province	Vietnam	Samuel and Knowles, 2001
HKN/1/2010	O	SEA	Mya-98	JQ070301	2/5/2010	pig	not known	Hong Kong	Knowles *et al* ., 2012
HKN/6/2010	O	SEA	Mya-98	JQ070302	2/10/2010	pig	not known	Hong Kong	Knowles *et al* ., 2012
MAY/5/2007	O	SEA	Mya-98	HQ116209	10/4/2007	cattle	d/a stesen Kuarantin Haiwan, Padang Besar, Perlis	Malaysia	Abdul-Hamid *et al* ., 2011
MYA/1/98	O	SEA	Mya-98	AJ303521	2/22/1998	cattle	Thar Zi, Mandalay	Myanmar	Samuel and Knowles, 2001
TAI/4/99	O	SEA	Mya-98	AJ303536	Mar-99	cattle	Mae Hong Son	Thailand	Samuel and Knowles, 2001
TAI/4/2007	O	SEA	Mya-98	HQ116240	9/10/2007	cattle	Sangcom, Udonthani	Thailand	Abdul-Hamid *et al* ., 2011
TAI/5/2007	O	SEA	Mya-98	HQ116241	9/12/2007	water buffalo	Muang, Khonkaen	Thailand	Abdul-Hamid *et al* ., 2011
TAI/12/2007	O	SEA	Mya-98	HQ116247	11/20/2007	cattle	Punpin, Suratthani	Thailand	Abdul-Hamid *et al* ., 2011
VIT/6/2006	O	SEA	Mya-98	HQ116289	3/1/2006	pig	Phu Yen province	Vietnam	Abdul-Hamid *et al* ., 2011
VN/HB139/2009	O	SEA	Mya-98	HM055494	10/1/2009	water buffalo	Hoa Binh province	Vietnam	unpublished*
VN/HB162/2009	O	SEA	Mya-98	HM055498	10/1/2009	water buffalo	Hoa Binh province	Vietnam	unpublished*
LAO/1/87	O	SEA	Tai-87	MK390898	7/15/1987	water buffalo	not known	Laos	This study
LAO/1/88	O	SEA	Tai-87	MK390899	1/13/1988	cattle	Hong village	Laos	This study
MAY/1/92	O	SEA	Tai-87	MK390911	6/9/1992	cattle	Kg. Tok. Rusa, Kubang Gadong, Pasir Mas, Kelantan	Malaysia	This study
MAY/5/92	O	SEA	Tai-87	MK390912	8/13/1992	cattle	Bharu, Tumpat, Kelantan	Malaysia	This study
MAY/5/93	O	SEA	Tai-87	MK390913	5/8/1993	cattle	Kg. Laklok, Bejut, Trengganu	Malaysia	This study
MYA/5/2002	O	SEA	Tai-87	HQ116222	9/23/2002	cattle	Pyay, Bago	Myanmar	Abdul-Hamid *et al* ., 2011
TAI/189/87	O	SEA	Tai-87	KY091288	1987	cattle	Udornthani	Thailand	This study
CAM/1/89	O	SEA	unnamed	MK390891	7/8/1989	cattle	Phum Tar Tea, Khum Krabey Real, Srok Puk. Prov. Siem Reap	Cambodia	This study
CAM/3/92	O	SEA	unnamed	MK390892	3/31/1992	cattle	Hong Ray	Cambodia	This study

Virus name	Serotype	Topotype	Lineage	Accession	Collected	Species	Location	Country	Reference
TAI/1/94	O	SEA	unnamed	MK390939	1994	not known	not known	Thailand	This study
ALG/1/99	O	WA	unnamed	AJ303481	Feb-99	cattle	of Greater	Algeria	Samuel and Knowles, 2001
CIV/8/99	O	WA	unnamed	AJ303485	1999	cattle	Cote d'Ivoire	Cote d'Ivoire	Samuel and Knowles, 2001
GHA/5/93	O	WA	unnamed	AJ303488	6/1/1993	cattle	Kintampo	Ghana	Samuel and Knowles, 2001
SRL/3/87	O	WCSA-1	Srl-77	MK390922	1987	not known	Udunuwara, Kandy District	Sri Lanka	This study
SRL/5/87	O	WCSA-1	Srl-77	MK390923	1987	not known	Ibbagamuwa	Sri Lanka	This study
SRL/10/88	O	WCSA-1	Srl-77	MK390933	1988	not known		Sri Lanka	This study
SRL/1/78	O	WCSA-1	Srl-77	MK390916	12/26/1977	not known	Kilinochchi	Sri Lanka	This study
SRL/2/78	O	WCSA-1	Srl-77	MK390917	1/11/1978	not known	Puttalam	Sri Lanka	This study
SRL/3/78	O	WCSA-1	Srl-77	MK390918	2/8/1978	not known	Gannoruwa	Sri Lanka	This study
SRL/87 (VS)	O	WCSA-1	Srl-77	MK390924	1/24/1987	bovine	Mahiyangana	Sri Lanka	This study
SRL/1/88	O	WCSA-1	Srl-77	MK390925	10/30/1987	bovine	Dummalasuriya	Sri Lanka	This study
SRL/2/88	O	WCSA-1	Srl-77	MK390926	11/2/1987	bovine	Horana	Sri Lanka	This study
SRL/3/88	O	WCSA-1	Srl-77	MK390927	11/9/1987	bovine	Mirigama	Sri Lanka	This study
SRL/4/88	O	WCSA-1	Srl-77	MK390928	11/19/1987	bovine	Ibbagamuwa	Sri Lanka	This study
SRL/6/88	O	WCSA-1	Srl-77	MK390929	12/1/1987	bovine	Kegalle	Sri Lanka	This study
SRL/7/88	O	WCSA-1	Srl-77	MK390930	12/3/1987	bovine	Ibbagamuwa	Sri Lanka	This study
SRL/8/88	O	WCSA-1	Srl-77	MK390931	12/7/1987	bovine	Ramboda	Sri Lanka	This study
SRL/9/88	O	WCSA-1	Srl-77	MK390932	12/8/1987	bovine	Kekirawa	Sri Lanka	This study
SRL/1/97	O	WCSA-1	Srl-77	MK390934	1/16/1997	bovine	Dekethiopthana, Anuradhadura, North-Central	Sri Lanka	This study
Rey/IRN/66	O	WCSA-1	Syr-62	AY593834	1966	not known	Rey	Iran	Carrillo et al., 2005
SYR/18/62	O	WCSA-1	Syr-62	MK390935	1962	not known	not known	Syria	This study
738/Tajikistan/USSR/74	O	WCSA-1	Syr-62	AJ004663	5/24/1974	cattle	Kanibdam district, Tajikistan	USSR	unpublished*
1492/Russia/USSR/88	O	WCSA-1	Syr-62	AJ004679	1/27/1988	pig	Rostov district, Yaroslavl', Russia	USSR	unpublished*

Knowles NJ, He J, Shang Y, Wadsworth J, Valdazo-González B, Onosato H, Fukai K, Morioka K, Yoshida K, Cho IS, Kim SM. Southeast Asian foot-and-mouth disease viruses in Eastern Asia. Emerging infectious diseases. 2012

Knowles NJ, Davies PR, Henry T, O'Donnell V, Pacheco JM, Mason PW. Emergence in Asia of foot-and-mouth disease viruses with altered host range: characterization of alterations in the 3A protein. Journal of Virology. 2001 Feb 1;75(3):1551-6.

Khounsy S, Conlan JV, Gleeson LJ, Westbury HA, Colling A, Paton DJ, Ferris NP, Valarcher JF, Wadsworth J, Knowles NJ, Blacksell SD. Molecular epidemiology of foot-and-mouth disease viruses from South East Asia

Samuel AR, Knowles NJ. Foot-and-mouth disease type O viruses exhibit genetically and geographically distinct evolutionary lineages (topotypes). Journal of General Virology. 2001 Mar 1;82(3):609-21.

Ayelet G, Mahapatra M, Gelaye E, Egziabher BG, Rufeal T, Sahle M, Ferris NP, Wadsworth J, Hutchings GH, Knowles NJ. Genetic characterization of foot-and-mouth disease viruses, Ethiopia, 1981–2007. Emerging infectious

Kasanga CJ, Wadsworth J, Mpelumbe-Ngeleja CA, Sallu R, Kivaria F, Wambura PN, Yongolo MG, Rweyemamu MM, Knowles NJ, King DP. Molecular characterization of foot-and-mouth disease viruses collected in Tanzania

Malirat V, de Barros JJ, Bergmann IE, de Mendonça Campos R, Neitzert E, da Costa EV, da Silva EE, Falczuk AJ, Pinheiro DS, de Vergara N, Cirvera JL. Phylogenetic analysis of foot-and-mouth disease virus type O re-emerging in

Forss S, Strebel K, Beck E, Schaller H. Nucleotide sequence and genome organization of foot-and-mouth disease virus. Nucleic Acids Research. 1984 Aug 24;12(16):6587-601.

Schumann KR, Knowles NJ, Davies PR, Midgley RJ, Valarcher JF, Raoufi AQ, McKenna TS, Hurtle W, Burans JP, Martin BM, Rodriguez LL. Genetic characterization and molecular epidemiology of foot-and-mouth disease viruses

Reeve R, Borley DW, Maree FF, Upadhyaya S, Lukhwareni A, Esterhuysen JJ, Harvey WT, Blignaut B, Fry EE, Parida S, Paton DJ. Tracking the antigenic evolution of foot-and-mouth disease virus. PloS one. 2016 Jul

Jamal SM, Ferrari G, Ahmed S, Normann P, Belsham GJ. Genetic diversity of foot-and-mouth disease virus serotype O in Pakistan and Afghanistan, 1997–2009. Infection, Genetics and Evolution. 2011 Aug 1;11(6):1229-38.

Mason PW, Pacheco JM, Zhao QZ, Knowles NJ. Comparisons of the complete genomes of Asian, African and European isolates of a recent foot-and-mouth disease virus type O pandemic strain (PanAsia). Journal of general virology.

Abdul-Hamid NF, Hussein NM, Wadsworth J, Radford AD, Knowles NJ, King DP. Phylogeography of foot-and-mouth disease virus types O and A in Malaysia and surrounding countries. Infection, Genetics and Evolution. 2011 Mar

Knowles NJ, Samuel AR, Davies PR, Midgley RJ, Valarcher JF. Pandemic strain of foot-and-mouth disease virus serotype O. Emerging infectious diseases. 2005 Dec;11(12):1887.

Valarcher JF, Leforban Y, Rweyemamu M, Roeder PL, Gerbier G, Mackay DK, Sumption KJ, Paton DJ, Knowles NJ. Incursions of Foot-and-Mouth Disease Virus into Europe between 1985 and 2006. Transboundary and emerging

Knowles NJ, Bachanek-Bankowska K, Wadsworth J, Mioulet V, Valdazo-González B, Eldaghayes IM, Dayhum AS, Kammon AM, Sharif MA, Waight S, Shamia AM. Outbreaks of Foot-and-Mouth Disease in Libya and Saudi Arabia

Abeyratne SA, Amarasekera SS, Ranaweera LT, Salpadoru TB, Thilakarathne SM, Knowles NJ, Wadsworth J, Puvanendiran S, Kothalawala H, Jayathilake BK, Wijithasiri HA. Correction: The phylogenetic analysis of VP1 genomic

Samuel, A.R., Knowles, N.J., Kitching, R.P. and Hafez, S.M., 1997. Molecular analysis of foot-and-mouth disease type O viruses isolated in Saudi Arabia between 1983 and 1995. Epidemiology & Infection, 119(3), pp.381-389.

Knowles NJ, Wadsworth J, Bachanek-Bankowska K, King DP. VP1 sequencing protocol for foot and mouth disease virus molecular epidemiology. Revue scientifique et technique (International Office of Epizootics). 2016 Dec

Carrillo C, Tulman ER, Delhon G, Lu Z, Carreno A, Vagnozzi A, Kutish GF, Rock DL. Comparative genomics of foot-and-mouth disease virus. Journal of virology. 2005 May 15;79(10):6487-504.

Rojas ER, Carrillo E, Schiappacassi M, Campos R. Modification of foot-and-mouth disease virus O1 Caseros after serial passages in the presence of antiviral polyclonal sera. Journal of virology. 1992 Jun 1;66(6):3368-72.

Cheung AN, DeLamarter JO, Weiss ST, Küpper H. Comparison of the major antigenic determinants of different serotypes of foot-and-mouth disease virus. Journal of virology. 1983 Nov 1;48(2):451-9.

Krebs O, Berger HG, Marquardt O. The capsid protein-encoding sequence of foot-and-mouth disease virus O 2 Brescia. Archives of virology. 1991 Mar 1;120(1-2):135-43.

Beard CW, Mason PW. Genetic determinants of altered virulence of Taiwanese foot-and-mouth disease virus. Journal of virology. 2000 Jan 15;74(2):987-91.

Unpublished*-http://www.ncbi.nlm.nih.gov/nucleotide/

Supplementary Table S2: The data generated in FUBA and FEL analyses.

AA Site	Fast Unconstrained Bayesian Approch								Fixed Effects Likelihood Approch					
	α	β	β-α	Prob[α>β]	Prob[α<β]	BayesFactor[α<β]	PSRF	Neff	alpha(synonymous substitution rate)	beta (non-synonymous substitution rate)	synonymous substitution rate=non-synonymous substitution rate	LRT	p-value	Total branch length
1	1.079	0.108	-0.972	0.937	0.044	0.127	1.001	430.523	0.216	0	0.056	2.656	0.103	0.371
2	0.997	0.112	-0.885	0.932	0.047	0.137	1.001	399.151	0.211	0	0.056	2.631	0.105	0.362
3	2.746	0.021	-2.725	1	0	0.001	1.043	55.996	1.071	0	0.255	12.116	0	1.836
4	3.228	2.404	-0.824	0.289	0.038	0.111	1.007	207.164	1.195	0.845	0.955	0.304	0.581	5.432
5	2.883	0.026	-2.857	1	0	0	1.03	75.473	1.449	0	0.406	16.542	0	2.484
6	3.778	0.062	-3.716	1	0	0	1.01	168.146	1.98	0	0.382	15.494	0	3.394
7	2.846	0.023	-2.823	1	0	0	1.042	56.797	1.143	0	0.274	11.716	0.001	1.959
8	1.365	0.104	-1.261	0.976	0.016	0.046	1.005	253.554	0.444	0	0.166	5.862	0.015	0.761
9	2.813	0.783	-2.031	0.892	0.059	0.176	0.999	669.586	1.323	0.285	0.506	3.44	0.064	3.407
10	1.667	0.086	-1.581	0.976	0.017	0.047	1.005	252.547	0.429	0	0.131	4.697	0.03	0.736
11	1.461	0.081	-1.38	0.962	0.025	0.072	1.002	366.163	0.246	0	0.058	2.871	0.09	0.422
12	2.939	0.023	-2.916	1	0	0	1.026	82.924	1.587	0	0.445	19.373	0	2.721
13	1.002	0.68	-0.322	0.598	0.323	1.336	0.999	685.85	0.453	0.282	0.331	0.243	0.622	1.904
14	2.888	0.023	-2.865	1	0	0	1.034	67.797	1.431	0	0.347	16.055	0	2.454
15	1.261	0.092	-1.168	0.981	0.012	0.035	1.006	222.057	0.408	0	0.146	6.08	0.014	0.699
16	2.47	0.059	-2.411	0.991	0.007	0.019	1.033	68.372	0.711	0	0.15	6.104	0.013	1.22
17	1.298	0.116	-1.181	0.935	0.047	0.137	1.001	422.286	0.292	0	0.062	3.039	0.081	0.501
18	1.325	0.108	-1.217	0.941	0.042	0.121	1.001	414.259	0.294	0	0.068	2.862	0.091	0.504
19	2.692	0.031	-2.661	0.999	0	0.001	1.031	73.069	0.921	0	0.319	12.085	0.001	1.578
20	2.818	0.027	-2.791	1	0	0	1.034	67.489	1.278	0	0.354	14.507	0	2.19
21	13.346	0.465	-12.882	1	0	0	1.004	285.529	4.799	0.095	0.701	21.132	0	8.61
22	2.206	0.051	-2.156	0.994	0.004	0.011	1.018	112.222	0.476	0	0.156	6.641	0.01	0.816
23	1.713	0.148	-1.564	0.926	0.057	0.169	1.008	193.282	0.356	0	0.099	2.516	0.113	0.61
24	0.985	0.495	-0.49	0.752	0.191	0.662	1.002	352.385	0.438	0.159	0.233	0.944	0.331	1.387
25	2.138	0.8	-1.338	0.749	0.17	0.572	1	460.332	0.847	0.294	0.433	0.999	0.318	2.629
26	3.631	0.041	-3.59	1	0	0	1.01	164.574	1.766	0	0.344	18.666	0	3.027
27	2.853	0.027	-2.826	1	0	0	1.033	68.693	1.309	0	0.373	14.256	0	2.243
28	6.681	1.029	-5.651	0.942	0.002	0.005	0.998	753.648	2.362	0.409	0.901	5.854	0.016	5.69
29	2.066	0.08	-1.986	0.98	0.014	0.04	1.016	124.954	0.633	0	0.148	5.567	0.018	1.085
30	2.781	0.025	-2.755	1	0	0.001	1.042	57.031	1.013	0	0.236	11.305	0.001	1.737
31	2.63	0.049	-2.581	0.996	0.003	0.008	1.034	68.342	1.087	0	0.213	9.031	0.003	1.864
32	1.496	0.323	-1.173	0.941	0.041	0.119	1.049	49.611	0.672	0.077	0.228	4.146	0.042	1.459
33	4.901	0.783	-4.118	0.987	0.002	0.006	0.998	875.086	1.591	0.385	0.731	4.608	0.032	4.268
34	5.408	0.055	-5.353	1	0	0	1.003	298.018	4.108	0	0.424	30.993	0	7.043
35	2.334	0.03	-2.305	0.994	0.004	0.011	1.023	92.174	0.474	0	0.047	4.618	0.032	0.813
36	3.165	0.035	-3.13	1	0	0	1.014	137.96	1.893	0	0.714	20.041	0	3.245
37	2.011	0.083	-1.928	0.978	0.015	0.044	1.014	137.315	0.608	0	0.139	5.68	0.017	1.042
38	2.68	0.028	-2.652	0.999	0.001	0.001	1.039	59.936	0.765	0	0.207	10.251	0.001	1.312
39	0.668	0.089	-0.579	0.935	0.04	0.116	1.002	353.858	0.161	0	0.045	2.549	0.11	0.276
40	1.416	0.083	-1.333	0.96	0.026	0.076	1.001	390.596	0.234	0	0.057	2.794	0.095	0.401
41	1.154	0.395	-0.759	0.849	0.113	0.355	1.016	120.61	0.433	0.08	0.175	1.996	0.158	1.064
42	5.827	0.491	-5.335	1	0	0	1.004	268.103	2.223	0.155	0.65	14.725	0	4.432
43	1.963	0.975	-0.988	0.724	0.166	0.556	1.003	311.403	0.641	0.436	0.508	0.28	0.597	2.845

AA Site	Fast Unconstrained Bayesian Approch								Fixed Effects Likelihood Approch					
	α	β	β-α	Prob[α>β]	Prob[α<β]	BayesFactor[α<β]	PSRF	Neff	alpha(synonymous substitution rate)	beta (non-synonymous substitution rate)	synonymous substitution rate=non-synonymous substitution rate	LRT	p-value	Total branch length
44	0.91	0.374	-0.536	0.829	0.128	0.411	1.004	265.283	0.306	0.095	0.176	0.969	0.325	0.904
45	3.193	2.65	-0.543	0.181	0.041	0.12	1.006	231.082	1.231	0.835	0.956	0.386	0.534	5.456
46	1.647	3.196	1.549	0.011	0.586	3.955	1.007	204.147	0.546	1.165	0.988	0.945	0.331	5.602
47	3.084	5.832	2.748	0.323	0.582	3.891	1.01	170.541	0	2.186	1.567	0.653	0.419	8.754
48	0.407	0.533	0.126	0.341	0.597	4.151	0.996	2821.859	0	0.128	0.078	0.997	0.318	0.514
49	2.157	0.698	-1.459	0.908	0.058	0.171	0.998	808.037	0.969	0.268	0.448	3.387	0.066	2.735
50	0.939	0.55	-0.388	0.731	0.205	0.721	1.001	447.064	0.487	0.16	0.267	1.471	0.225	1.474
51	9.982	0.511	-9.471	1	0	0	1.003	330.835	3.777	0.16	0.853	23.216	0	7.115
52	2.933	0.029	-2.904	1	0	0	1.025	87.418	1.492	0	0.523	15.191	0	2.558
53	1.26	0.131	-1.129	0.923	0.056	0.167	1.001	434.966	0.29	0	0.069	2.817	0.093	0.497
54	1.043	0.137	-0.906	0.942	0.041	0.12	1.004	270.582	0.293	0	0.123	3.424	0.064	0.502
55	3.141	0.04	-3.101	0.971	0.018	0.05	1.005	251.476	0	0	0	0	1	0
56	2.677	0.07	-2.608	0.994	0.004	0.012	1.033	69.073	0.958	0	0.283	7.214	0.007	1.643
57	0.373	0.755	0.382	0.154	0.802	11.348	0.995	5718.031	0	0.325	0.236	2.531	0.112	1.303
58	2.983	0.027	-2.956	1	0	0	1.024	89.368	1.682	0	0.525	17.77	0	2.884
59	7.152	0.393	-6.759	1	0	0	1.004	288.879	2.742	0.091	0.81	19.697	0	5.066
60	0.806	0.428	-0.379	0.703	0.239	0.879	1.001	435.858	0.307	0.1	0.15	0.583	0.445	0.926
61	2.602	0.031	-2.571	0.999	0.001	0.002	1.035	65.632	0.878	0	0.226	10.431	0.001	1.505
62	2.876	0.027	-2.849	1	0	0	1.026	83.031	1.416	0	0.525	14.966	0	2.428
63	2.827	0.023	-2.805	1	0	0	1.042	56.249	1.062	0	0.239	11.796	0.001	1.821
64	2.735	0.03	-2.705	0.999	0	0.001	1.036	63.95	0.855	0	0.283	10.771	0.001	1.466
65	7.727	0.151	-7.576	1	0	0	1.011	163.139	3.2	0	0.709	28.449	0	5.486
66	1.836	0.064	-1.773	0.986	0.009	0.027	1.009	186.684	0.459	0	0.119	5.304	0.021	0.787
67	4.251	0.056	-4.195	1	0	0	1.003	304.643	2.435	0	0.691	25.669	0	4.175
68	2.48	0.043	-2.436	0.997	0.002	0.005	1.026	82.908	0.783	0	0.252	9.25	0.002	1.342
69	2.154	0.987	-1.168	0.778	0.11	0.347	1.004	284.094	0.941	0.438	0.601	1.314	0.252	3.369
70	2.649	0.033	-2.616	0.999	0.001	0.002	1.036	64.239	0.929	0	0.252	9.895	0.002	1.593
71	0.569	0.133	-0.436	0.877	0.085	0.26	1.003	331.181	0.13	0	0.048	1.973	0.16	0.222
72	0.599	0.151	-0.448	0.747	0.184	0.632	1.001	422.157	0	0	0	0	1	0
73	2.944	0.027	-2.917	1	0	0	1.03	75.201	1.753	0	0.448	17.593	0	3.005
74	2.285	0.033	-2.252	0.996	0.002	0.007	1.024	88.195	0.696	0	0.105	7.278	0.007	1.193
75	1.781	0.355	-1.426	0.963	0.023	0.066	1.06	41.814	0.764	0.088	0.338	5.324	0.021	1.662
76	2.891	0.032	-2.859	1	0	0	1.031	73.811	1.588	0	0.414	15.956	0	2.722
77	2.819	0.026	-2.794	1	0	0	1.027	82.344	1.184	0	0.531	14.357	0	2.03
78	1.374	0.125	-1.249	0.933	0.049	0.143	1.002	384.109	0.262	0	0.068	2.729	0.099	0.45
79	0.799	0.351	-0.448	0.772	0.176	0.598	1.002	384.582	0.228	0.083	0.122	0.474	0.491	0.725
80	2.769	0.028	-2.74	0.999	0	0.001	1.041	57.773	0.941	0	0.259	10.201	0.001	1.614
81	2.714	0.026	-2.688	0.999	0.001	0.002	1.045	53.866	0.947	0	0.188	9.83	0.002	1.623
82	2.931	0.388	-2.543	0.983	0.011	0.03	1.075	34.884	1.049	0.082	0.312	6.71	0.01	2.126
83	2.044	0.081	-1.962	0.979	0.015	0.042	1.015	129.607	0.622	0	0.147	5.52	0.019	1.067
84	1.028	0.61	-0.418	0.613	0.321	1.321	0.999	579.117	0.343	0.199	0.231	0.177	0.674	1.386
85	3.6	0.047	-3.553	1	0	0	1.01	171.668	1.797	0	0.446	18.793	0	3.08
86	2.036	0.57	-1.466	0.879	0.086	0.262	1.004	266.593	0.975	0.165	0.326	3.487	0.062	2.332

AA Site	Fast Unconstrained Bayesian Approch								Fixed Effects Likelihood Approch					
	α	β	β-α	Prob[α>β]	Prob[α<β]	BayesFactor[α<β]	PSRF	Neff	alpha(synonymous substitution rate)	beta (non-synonymous substitution rate)	synonymous substitution rate=non-synonymous substitution rate	LRT	p-value	Total branch length
87	1.043	0.101	-0.942	0.941	0.04	0.116	1.001	426.21	0.211	0	0.058	2.554	0.11	0.362
88	2.553	0.036	-2.517	0.997	0.002	0.005	1.035	65.821	0.827	0	0.176	8.69	0.003	1.418
89	3.342	0.092	-3.25	0.939	0.045	0.131	1.007	218.383	0	0	0	0	1	0
90	2.996	0.023	-2.973	1	0	0	1.03	74.451	1.542	0	0.343	16.411	0	2.644
91	2.948	0.026	-2.922	1	0	0	1.031	73.37	1.244	0	0.393	13.498	0	2.133
92	2.952	0.03	-2.923	1	0	0	1.03	75.25	1.781	0	0.377	18.037	0	3.053
93	2.483	0.044	-2.438	0.996	0.003	0.008	1.032	70.6	0.644	0	0.182	7.489	0.006	1.105
94	3.696	0.047	-3.649	1	0	0	1.007	206.618	1.855	0	0.576	20.789	0	3.181
95	2.557	0.036	-2.521	0.998	0.001	0.003	1.027	80.316	0.8	0	0.295	9.606	0.002	1.371
96	2.499	0.059	-2.439	0.991	0.006	0.018	1.033	68.65	0.758	0	0.15	6.184	0.013	1.3
97	2.602	0.816	-1.786	0.916	0.04	0.117	1.001	435.299	0.927	0.342	0.53	2.066	0.151	2.957
98	2.837	0.027	-2.81	1	0	0	1.037	62.864	1.088	0	0.311	11.913	0.001	1.865
99	1.5	0.108	-1.392	0.974	0.018	0.05	1.004	284.569	0.429	0	0.182	5.041	0.025	0.736
100	2.195	1.001	-1.194	0.69	0.188	0.65	1.001	394.633	1.016	0.405	0.537	1.146	0.284	3.363
101	1.365	0.112	-1.252	0.939	0.044	0.128	1.001	393.625	0.304	0	0.063	3.099	0.078	0.522
102	1.472	0.652	-0.82	0.779	0.162	0.542	0.999	624.619	0.689	0.241	0.361	1.529	0.216	2.148
103	1.03	0.11	-0.92	0.933	0.046	0.134	1.001	416.806	0.211	0	0.056	2.625	0.105	0.362
104	3.462	0.044	-3.418	1	0	0	1.015	129.93	2.64	0	0.377	19.823	0	4.525
105	0.891	0.128	-0.762	0.915	0.06	0.177	1.002	355.353	0.154	0	0.058	1.936	0.164	0.265
106	2.483	0.037	-2.446	0.997	0.002	0.005	1.033	68.624	0.618	0	0.168	7.69	0.006	1.06
107	2.743	0.03	-2.713	0.999	0	0.001	1.04	59.387	0.913	0	0.254	9.92	0.002	1.565
108	2.588	0.041	-2.547	0.997	0.002	0.006	1.034	66.998	0.998	0	0.206	8.795	0.003	1.71
109	0.582	0.177	-0.405	0.717	0.214	0.763	1.001	393.085	0	0	0	0	1	0
110	4.367	0.069	-4.298	1	0	0	1.004	271.459	2.541	0	0.356	18.38	0	4.356
111	2.54	0.366	-2.173	0.987	0.008	0.022	1.089	30.327	0.834	0.091	0.356	5.633	0.018	1.795
112	2.231	0.364	-1.867	0.979	0.013	0.037	1.083	32.185	0.833	0.097	0.369	5.288	0.021	1.814
113	1.948	0.059	-1.889	0.988	0.008	0.023	1.012	150.78	0.494	0	0.121	5.5	0.019	0.847
114	5.592	0.095	-5.497	1	0	0	1.004	265.475	2.884	0	0.634	29.182	0	4.943
115	2.987	0.033	-2.954	1	0	0	1.028	80.016	1.291	0	0.413	13.355	0	2.214
116	0.877	0.11	-0.766	0.956	0.03	0.085	1.007	214.613	0.289	0	0.104	4.045	0.044	0.496
117	4.749	0.078	-4.671	1	0	0	1.002	336.041	2.275	0	0.603	22.797	0	3.901
118	2.751	0.031	-2.72	1	0	0.001	1.033	69.27	0.982	0	0.383	10.715	0.001	1.684
119	7.233	0.151	-7.082	1	0	0	1.009	176.884	3.321	0	0.901	30.552	0	5.693
120	1.414	0.103	-1.311	0.947	0.038	0.109	1.002	370.46	0.322	0	0.07	3.003	0.083	0.552
121	2.509	0.036	-2.474	0.997	0.002	0.005	1.035	66.004	0.641	0	0.17	7.864	0.005	1.099
122	3.592	0.044	-3.548	1	0	0	1.009	179.142	1.722	0	0.51	18.918	0	2.951
123	2.589	0.035	-2.554	0.998	0.001	0.003	1.034	67.721	0.647	0	0.239	7.846	0.005	1.109
124	1.243	0.132	-1.111	0.922	0.057	0.169	1.001	439.217	0.286	0	0.073	2.691	0.101	0.491
125	0.617	0.15	-0.468	0.87	0.093	0.288	1.003	320.514	0.141	0	0.054	1.894	0.169	0.241
126	2.784	0.021	-2.763	1	0	0	1.04	58.718	1.156	0	0.293	13.792	0	1.982
127	2.683	0.033	-2.651	0.999	0	0.001	1.031	73.755	0.871	0	0.365	10.114	0.001	1.492
128	2.974	0.026	-2.947	1	0	0	1.024	88.983	1.478	0	0.479	17.129	0	2.534
129	2.489	0.037	-2.452	0.997	0.002	0.005	1.033	68.371	0.8	0	0.177	8.704	0.003	1.371

AA Site	Fast Unconstrained Bayesian Approch								Fixed Effects Likelihood Approch					
	α	β	β-α	Prob[α>β]	Prob[α<β]	BayesFactor[α<β]	PSRF	Neff	alpha(synonymous substitution rate)	beta (non-synonymous substitution rate)	synonymous substitution rate=non-synonymous substitution rate	LRT	p-value	Total branch length
130	6.89	0.506	-6.383	1	0	0	1.002	355.904	2.708	0.161	0.795	18.295	0	5.287
131	3.014	0.034	-2.98	0.999	0	0.001	1.033	69.073	1.744	0	0.284	12.756	0	2.991
132	0.593	0.16	-0.433	0.737	0.195	0.677	1.001	410.932	0	0	0	0	1	0
133	2.956	0.03	-2.926	1	0	0	1.033	69.789	1.233	0	0.309	13.201	0	2.114
134	6.185	0.395	-5.79	1	0	0	1.015	129.081	2.972	0.079	0.506	18.057	0	5.411
135	0.784	0.331	-0.454	0.787	0.163	0.545	1.001	421.509	0.293	0.073	0.116	0.881	0.348	0.794
136	5.676	0.578	-5.098	0.99	0.005	0.015	1	505.808	2.212	0.17	0.442	8.192	0.004	4.472
137	3.703	0.047	-3.656	1	0	0	1.011	154.622	2.996	0	0.419	19.028	0	5.136
138	2.367	0.706	-1.661	0.898	0.064	0.193	0.999	668.347	1.008	0.269	0.462	2.784	0.095	2.805
139	2.294	3.865	1.571	0.02	0.389	1.784	1.005	243.399	0.942	1.476	1.314	0.456	0.5	7.525
140	2.715	2.866	0.152	0.044	0.146	0.479	1.005	258.351	1.112	1.171	1.156	0.007	0.935	6.599
141	3.179	2.643	-0.536	0.177	0.029	0.083	1.006	231.461	1.233	1.069	1.129	0.061	0.805	6.394
142	5.798	2.019	-3.779	0.739	0	0.001	0.999	698.961	2.35	0.811	1.268	4.067	0.044	7.276
143	4.206	0.959	-3.246	0.943	0.006	0.016	1	554.725	1.817	0.412	0.73	5.434	0.02	4.763
144	12.98	0.855	-12.126	0.998	0	0	0.999	665.571	6.181	0.337	0.953	17.461	0	11.946
145	1.994	0.484	-1.51	0.919	0.056	0.167	1.015	128.917	0.753	0.155	0.296	2.872	0.09	1.913
146	2.629	0.032	-2.596	0.998	0.001	0.003	1.04	59.549	0.81	0	0.17	9.237	0.002	1.388
147	0.923	0.133	-0.789	0.942	0.041	0.119	1.006	237.667	0.299	0	0.107	4.088	0.043	0.512
148	2.986	0.041	-2.945	0.999	0	0.001	1.032	71.411	1.928	0	0.306	13.68	0	3.306
149	4.787	0.068	-4.718	1	0	0	1.002	337.197	2.627	0	0.615	26.28	0	4.504
150	2.22	0.104	-2.116	0.973	0.019	0.055	1.021	100.457	0.574	0	0.182	4.488	0.034	0.984
151	2.649	0.028	-2.621	0.999	0.001	0.002	1.042	56.833	0.826	0	0.177	8.913	0.003	1.415
152	2.796	0.027	-2.769	1	0	0	1.031	72.044	1.111	0	0.44	12.366	0	1.905
153	4.36	0.7	-3.66	0.992	0.001	0.002	1	495.517	1.764	0.275	0.723	7.842	0.005	4.126
154	1.037	0.532	-0.505	0.677	0.263	0.999	1.004	276.975	0.341	0.143	0.201	0.357	0.55	1.157
155	1.077	0.571	-0.506	0.655	0.283	1.103	1	473.482	0.389	0.171	0.211	0.387	0.534	1.353
156	2.997	0.029	-2.968	1	0	0	1.032	71.189	1.314	0	0.332	13.339	0	2.253
157	0.826	0.384	-0.441	0.752	0.195	0.678	1.002	345.46	0.247	0.091	0.133	0.469	0.493	0.788
158	3.923	0.345	-3.578	0.999	0.001	0.002	1.079	33.369	1.295	0.072	0.378	10.76	0.001	2.509
159	1.697	1.861	0.164	0.25	0.434	2.146	1.011	158.238	0.569	0.785	0.721	0.152	0.696	4.12
160	0.571	0.416	-0.155	0.611	0.32	1.314	0.996	2373.566	0.141	0.108	0.122	0.036	0.849	0.673
161	2.899	0.024	-2.874	1	0	0	1.027	81.089	1.554	0	0.496	16.585	0	2.665
162	2.934	0.025	-2.908	1	0	0	1.035	66.671	1.415	0	0.295	14.386	0	2.426
163	3.125	0.024	-3.101	1	0	0	1.021	99.556	1.839	0	0.455	20.419	0	3.152
164	2.644	0.022	-2.622	0.999	0	0.001	1.04	58.887	1.106	0	0.156	10.804	0.001	1.896
165	2.538	0.044	-2.494	0.996	0.003	0.007	1.033	68.487	0.942	0	0.189	9.298	0.002	1.616
166	19.278	0.567	-18.711	1	0	0	1.007	216.599	8.16	0.18	1.15	31.881	0	14.711
167	0.558	0.153	-0.405	0.853	0.105	0.33	1.002	336.547	0.127	0	0.05	1.847	0.174	0.217
168	1.7	0.084	-1.616	0.976	0.016	0.046	1.006	238.989	0.429	0	0.124	4.865	0.027	0.736
169	2.809	0.021	-2.788	1	0	0	1.041	57.545	1.236	0	0.211	13.252	0	2.119
170	2.94	0.035	-2.905	0.999	0	0.001	1.035	65.673	1.246	0	0.258	11.973	0.001	2.136
171	2.609	0.034	-2.575	0.998	0.001	0.003	1.035	66.042	0.876	0	0.251	9.73	0.002	1.503
172	14.259	2.988	-11.271	0.974	0	0	0.996	3607.227	4.939	1.179	2.127	7.173	0.007	13.19

AA Site	Fast Unconstrained Bayesian Approch								Fixed Effects Likelihood Approch					
	α	β	β-α	Prob[α>β]	Prob[α<β]	BayesFactor[α<β]	PSRF	Neff	alpha(synonymous substitution rate)	beta (non-synonymous substitution rate)	synonymous substitution rate=non-synonymous substitution rate	LRT	p-value	Total branch length
173	0.771	1.159	0.388	0.229	0.682	6.004	1	531.299	0.198	0.572	0.442	0.913	0.339	2.628
174	2.856	0.023	-2.833	1	0	0	1.041	57.761	1.187	0	0.242	12.275	0	2.035
175	0.261	0.528	0.267	0.193	0.753	8.557	0.996	2908.925	0	0.159	0.097	1.963	0.161	0.638
176	2.833	0.038	-2.795	0.999	0.001	0.002	1.037	63.049	1.047	0	0.27	10.498	0.001	1.794
177	2.756	0.03	-2.726	1	0	0.001	1.033	68.699	0.993	0	0.386	10.823	0.001	1.702
178	2.848	0.019	-2.829	1	0	0	1.033	69.465	1.428	0	0.445	17.832	0	2.449
179	0.599	0.151	-0.448	0.747	0.184	0.632	1.001	422.157	0	0	0	0	1	0
180	1.646	0.135	-1.511	0.902	0.085	0.262	1.011	161.214	0.459	0	0.128	3.597	0.058	0.787
181	3.141	0.04	-3.101	0.971	0.018	0.05	1.005	251.476	0	0	0	0	1	0
182	4.219	0.068	-4.151	1	0	0	1.004	287.316	2.298	0	0.405	19.785	0	3.94
183	2.311	0.047	-2.264	0.993	0.005	0.014	1.028	78.353	0.534	0	0.113	6.128	0.013	0.916
184	2.804	0.024	-2.78	1	0	0	1.033	69.913	1.185	0	0.406	14.354	0	2.032
185	2.616	0.051	-2.565	0.994	0.004	0.011	1.04	58.969	0.981	0	0.192	8.322	0.004	1.681
186	4.975	0.525	-4.45	0.999	0	0.001	1.006	238.977	1.721	0.163	0.545	9.972	0.002	3.605
187	1.41	0.103	-1.306	0.946	0.038	0.111	1.002	373.575	0.317	0	0.069	2.974	0.085	0.544
188	2.869	0.019	-2.85	1	0	0	1.036	65.135	1.53	0	0.326	17.596	0	2.622
189	2.949	0.026	-2.924	1	0	0	1.025	85.809	1.599	0	0.506	17.022	0	2.74
190	3.201	0.037	-3.164	1	0	0	1.021	98.314	1.48	0	0.405	15.148	0	2.537
191	1.694	0.085	-1.609	0.983	0.011	0.032	1.005	258.463	0.484	0	0.178	5.878	0.015	0.83
192	2.488	0.034	-2.454	0.999	0.001	0.002	1.024	89.789	0.746	0	0.264	10.046	0.002	1.28
193	2.68	0.033	-2.647	0.998	0.001	0.003	1.044	54.283	0.917	0	0.369	9.295	0.002	1.573
194	2.705	0.029	-2.676	0.999	0	0.001	1.036	64.202	0.995	0	0.306	11.455	0.001	1.705
195	5.932	0.948	-4.985	0.978	0	0.001	0.999	610.122	2.357	0.452	0.832	7.407	0.006	5.85
196	0.41	0.768	0.358	0.184	0.768	9.242	0.996	4443.422	0	0.33	0.233	2.099	0.147	1.322
197	2.821	0.028	-2.792	1	0	0.001	1.04	58.498	1.03	0	0.278	10.273	0.001	1.766
198	3.181	1.49	-1.692	0.675	0.053	0.155	1.008	197.735	1.569	0.518	0.701	2.646	0.104	4.766
199	2.93	0.829	-2.101	0.863	0.076	0.23	0.998	790.868	1.091	0.306	0.481	2.163	0.141	3.096
200	1.41	0.368	-1.042	0.914	0.06	0.179	1.034	67.663	0.701	0.089	0.266	3.868	0.049	1.558
201	0.986	0.114	-0.872	0.931	0.048	0.141	1.001	388.315	0.169	0	0.05	2.43	0.119	0.29
202	0.582	0.177	-0.405	0.717	0.214	0.763	1.001	393.085	0	0	0	0	1	0
203	6.852	0.121	-6.731	1	0	0	1.004	275.838	3.198	0	0.475	23.427	0	5.482
204	2.329	0.096	-2.233	0.978	0.016	0.045	1.025	85.566	0.663	0	0.196	4.895	0.027	1.136
205	2.984	0.392	-2.592	0.97	0.02	0.058	1.065	39.378	1.066	0.084	0.272	5.563	0.018	2.165
206	0.917	0.093	-0.824	0.967	0.021	0.06	1.007	215.108	0.287	0	0.089	4.662	0.031	0.493
207	2.2	0.047	-2.153	0.992	0.005	0.014	1.023	93.992	0.497	0	0.116	5.719	0.017	0.852
208	1.439	0.099	-1.34	0.969	0.021	0.061	1.003	311.528	0.296	0	0.11	3.924	0.048	0.507
209	2.453	0.042	-2.41	0.998	0.001	0.004	1.022	97.576	0.734	0	0.287	9.164	0.002	1.258
210	2.663	2.552	-0.111	0.143	0.129	0.413	1.008	191.869	0.847	0.932	0.904	0.023	0.881	5.183
211	6.217	0.404	-5.813	1	0	0	1.012	151.076	2.098	0.081	0.5	14.56	0	3.922
212	6.55	0.167	-6.383	1	0	0	1.002	354.679	3.338	0	0.611	16.543	0	5.723
213	3.247	2.489	-0.759	0.255	0.024	0.068	1.006	225.257	1.499	0.923	1.094	0.702	0.402	6.266
214	2.663	0.394	-2.269	0.992	0.004	0.011	1.08	33.123	1.108	0.109	0.51	6.955	0.008	2.335

Supplementary Table S3: The deduced amino acid sequences.

Topotype	Liniage	Isolate	1	2	3	4	5	6	7	8	9	10	11	12	13	14	15	16	17	18	19	20	21	22	23	24	25	26	27	28	29	30	31	32	33	34	35	36	37	38	39	40	41	42	43	44	45	46	47	48	49	50	51	52	53	54	55	56	57	58	59	60	61	62	63	64	65	66	67	68	69	70	71	72	73	74	75	76	77	78	79	80	81	82	83	84	85	86	87	88	89	90	91	92	93	94	95	96
Reference Sequnce		NC 004004 ref seq	T	T	S	A	G	E	S	A	D	P	V	T	A	T	V	E	N	Y	G	G	E	T	Q	V	Q	R	R	Q	H	T	D	I	A	F	I	L	D	R	F	V	K	V	K	P	K	-	E	Q	V	N	V	L	D	L	M	Q	I	P	A	H	T	L	V	G	A	L	L	R	T	A	T	Y	Y	F	S	D	L	E	L	A	V	K	H	E	G	D	L	T	W	V	P	N	G	A	P	E
SEA	May-78	O/MAY/1/1980	-	-	-	T	-	-	-	-	-	-	-	-	T	-	-	-	-	-	-	-	-	-	-	-	-	-	-	-	-	-	-	V	S	-	-	-	-	-	-	-	-	-	T	-	-	-	D	-	I	-	-	-	-	-	-	-	T	-	-	-	-	-	-	-	-	-	-	-	-	-	-	-	-	-	A	-	-	-	V	-	-	-	-	-	-	N	-	-	-	-	-	-	-	-	-	-
		O/TAI/2/1980	-	-	-	T	-	-	-	-	-	-	-	-	T	-	-	-	-	-	-	-	-	-	-	-	-	-	-	-	-	-	-	V	S	-	-	-	-	-	-	-	-	-	T	-	-	-	D	-	I	-	-	-	-	-	-	-	T	-	-	-	-	-	-	-	-	-	-	-	-	-	-	-	-	-	A	-	-	-	V	-	-	-	-	-	-	N	-	-	-	-	-	-	-	-	-	-
		O/MAY/9/1980	-	-	-	T	-	-	-	-	-	-	-	-	T	-	-	-	-	-	-	-	-	-	-	-	-	-	-	-	-	-	-	V	S	-	-	-	-	-	-	-	-	-	T	-	-	-	D	-	I	-	-	-	-	-	-	-	T	-	-	-	-	-	-	-	-	-	-	-	-	-	-	-	-	-	A	-	-	-	V	-	-	-	-	-	-	N	-	-	-	-	-	-	-	-	-	-
		O/TAI/3/1980	-	-	-	T	-	-	-	-	-	-	-	-	T	-	-	-	-	-	-	-	-	-	-	-	-	-	-	-	-	-	-	V	S	-	-	-	-	-	-	-	-	-	T	-	-	-	D	-	I	-	-	-	-	-	-	-	T	-	-	-	-	-	-	-	-	-	-	-	A	-	-	-	-	-	A	-	-	-	V	-	-	-	-	-	-	N	-	-	-	-	-	-	-	-	-	-
		O/MAY/11/1980	-	-	-	-	-	-	-	-	-	-	-	-	T	-	-	-	-	-	-	-	-	-	-	-	-	-	-	-	-	-	-	V	S	-	-	-	-	-	-	-	-	-	T	-	-	-	D	-	I	-	-	-	-	-	-	-	T	-	-	-	-	-	-	-	-	-	-	-	-	-	-	-	-	-	A	-	-	-	V	-	-	-	-	-	-	N	-	-	-	-	-	-	-	-	-	-
		O/TAI/1/1980	-	-	-	T	-	-	-	-	-	-	-	-	T	-	-	-	-	-	-	-	-	-	-	-	-	-	-	-	-	-	-	V	S	-	-	-	-	-	-	-	-	-	T	-	-	-	D	-	I	-	-	-	-	-	-	-	T	-	-	-	-	-	-	-	-	-	-	-	-	-	-	-	-	-	A	-	-	-	V	-	-	-	-	-	-	N	-	-	-	-	-	-	-	-	-	-
		O/MAY/7/1981	-	-	-	T	-	-	-	-	-	-	-	-	T	-	-	-	-	-	-	-	-	-	-	A	-	-	-	-	-	-	-	V	S	-	-	-	-	-	-	-	-	-	T	-	-	-	D	-	I	-	-	-	-	-	-	-	T	-	-	-	-	-	-	-	-	-	-	-	-	-	-	-	-	-	A	-	-	-	V	-	-	-	-	-	-	N	-	-	-	-	-	-	-	-	-	-
		O/MAY/16/1981	-	-	-	T	-	-	-	-	-	-	-	-	T	-	-	-	-	-	-	-	-	-	-	A	-	-	-	-	-	-	-	V	S	-	-	-	-	-	-	-	-	-	T	-	-	-	D	-	I	-	-	-	-	-	-	-	T	-	-	-	-	-	-	-	-	-	-	-	-	-	-	-	-	-	A	-	-	-	V	-	-	-	-	-	-	N	-	-	-	-	-	-	-	-	-	-
		O/LAO/2/1981	-	-	-	T	-	-	-	-	-	-	-	-	T	-	-	-	-	-	-	-	-	-	-	-	-	-	-	-	-	-	-	V	S	-	-	-	-	-	-	-	-	-	T	-	-	-	D	-	I	-	-	-	-	-	-	-	T	-	-	-	-	-	-	-	-	-	-	-	-	-	-	-	-	-	A	-	-	-	V	-	-	-	-	-	-	N	-	-	-	-	-	-	-	-	-	-
		O/HKN/17/82/AJ294918/1982	-	-	-	T	-	-	-	-	-	-	-	-	T	-	-	-	-	-	-	-	-	-	-	-	-	-	-	-	-	-	-	V	S	-	-	-	-	-	-	-	-	-	T	-	-	-	D	-	I	-	-	-	-	-	-	-	T	-	-	-	-	-	-	-	-	-	-	-	-	-	-	-	-	-	A	-	-	-	V	-	-	-	-	-	-	N	-	-	-	-	-	-	-	-	-	-
		O/MAY/19/1978	-	-	-	T	-	-	-	-	-	-	-	-	-	-	-	-	-	-	-	-	-	-	-	-	-	-	-	-	-	-	-	V	S	-	-	-	-	-	-	-	-	-	T	-	-	-	D	-	I	-	-	-	-	-	-	-	T	-	-	-	-	-	-	-	-	-	-	-	-	-	-	-	-	-	A	-	-	-	V	-	-	-	-	-	-	N	-	-	-	-	-	-	-	-	-	-
		O/MAY/24/1978	-	-	-	T	-	-	-	-	-	-	-	-	-	-	-	-	-	-	-	-	-	-	-	-	-	-	-	-	-	-	-	V	S	-	-	-	-	-	-	-	-	-	T	-	-	-	D	-	I	-	-	-	-	-	-	-	T	-	-	-	-	-	-	-	-	-	-	-	-	-	-	-	-	-	A	-	-	-	V	-	-	-	-	-	-	N	-	-	-	-	-	-	-	-	-	-
		O/MAY/1/1978	-	-	-	T	-	-	-	-	-	-	-	-	-	-	-	-	-	-	-	-	-	-	-	-	-	-	-	-	-	-	-	V	S	-	-	-	-	-	-	-	-	-	T	-	-	-	D	-	I	-	-	-	-	-	-	-	T	-	-	-	-	-	-	-	-	-	-	-	-	-	-	-	-	-	A	-	-	-	V	-	-	-	-	-	-	N	-	-	-	-	-	-	-	-	-	-
		O/MAY/3/1979	-	-	-	T	-	-	-	-	-	-	-	-	-	-	-	-	-	-	-	-	-	-	-	-	-	-	-	-	-	-	-	V	S	-	-	-	-	-	-	-	-	-	T	-	-	-	D	-	I	-	-	-	-	-	-	-	T	-	-	-	-	-	-	-	-	-	-	-	-	-	-	-	-	-	A	-	-	-	V	-	-	-	-	-	-	N	-	-	-	-	-	-	-	-	-	-
		O/MAY/1/1984	-	-	-	T	-	-	-	-	-	-	-	-	T	-	-	-	-	-	-	-	-	-	-	-	-	-	-	-	-	-	-	V	S	-	-	-	-	-	-	-	-	-	T	S	-	-	D	-	I	-	-	-	-	-	-	-	T	-	-	-	-	-	-	-	-	-	-	-	-	-	-	-	-	-	A	-	-	-	V	-	-	-	-	-	-	N	-	-	-	-	-	-	-	-	-	-
		O/SRL/3/1984	-	-	-	T	-	-	-	-	-	-	-	-	-	-	-	-	-	-	-	-	-	-	-	-	-	-	-	-	-	-	-	V	S	-	-	-	-	-	-	-	-	-	T	-	-	-	D	-	I	-	-	-	-	-	-	-	T	-	-	-	-	-	-	-	-	-	-	-	-	-	-	-	-	-	A	-	-	-	V	-	-	-	-	-	-	N	-	-	-	-	-	-	-	-	-	-
		O/SRL/2/1984	-	-	-	T	-	-	-	-	-	-	-	-	-	-	-	-	-	-	-	-	-	-	-	-	-	-	-	-	-	-	-	V	S	-	-	-	-	-	-	-	-	-	T	-	-	-	D	-	I	-	-	-	-	-	-	-	T	-	-	-	-	-	-	-	-	-	-	-	-	-	-	-	-	-	A	-	-	-	V	-	-	-	-	-	-	N	-	-	-	-	-	-	-	-	-	-
		O/MAY/3/1981	-	-	-	T	-	-	-	-	V	-	-	-	-	-	-	-	-	-	-	-	-	-	-	-	-	-	-	-	-	-	-	V	S	-	-	-	-	-	-	-	-	-	T	-	-	-	D	-	I	-	-	-	-	-	-	-	A	-	-	-	-	-	-	-	-	-	-	-	-	-	-	-	-	-	A	-	-	-	V	-	-	-	-	-	-	N	-	-	-	-	-	-	-	-	-	-
		O/MAY/10/1994	-	-	-	T	-	-	-	-	-	-	-	-	-	-	-	-	-	-	-	-	-	-	-	-	-	-	-	-	-	-	-	V	S	-	-	-	-	-	-	-	-	-	T	-	Q	-	S	-	I	-	-	-	-	-	-	-	T	-	-	-	-	-	-	-	-	-	-	-	-	-	-	-	-	-	A	-	-	-	V	-	-	-	-	-	-	N	-	-	-	-	-	-	-	-	-	-
		O/TAI/6/1995	-	-	-	T	-	-	-	-	-	-	-	-	-	-	-	-	-	-	-	-	-	-	-	-	-	-	-	-	-	-	-	V	S	-	-	-	-	-	-	-	-	-	T	-	Q	-	N	-	I	-	-	-	-	-	-	-	T	-	-	-	-	-	-	-	-	-	-	-	-	-	-	-	-	-	A	-	-	-	V	-	-	-	-	-	-	N	-	-	-	-	-	-	-	-	-	-
		O/TAI/2/2000/DQ164979	-	-	-	T	-	-	-	-	-	-	-	-	-	-	-	-	-	-	-	-	-	-	-	-	-	-	-	-	-	-	-	V	S	-	-	-	-	-	-	-	-	-	T	-	Q	-	D	-	T	-	-	-	-	-	-	-	T	-	-	-	-	-	-	-	-	-	-	-	A	-	-	-	-	-	A	-	-	-	V	-	-	-	-	-	-	N	-	-	-	-	-	-	-	-	-	-
	Bur-77	O/BUR/2/1977	-	-	-	-	-	-	-	-	-	-	-	-	-	-	-	-	-	-	-	-	-	-	-	-	-	-	-	-	-	-	-	V	S	-	-	-	-	-	-	-	-	I	T	-	-	-	D	-	I	-	-	-	-	-	-	-	T	-	-	-	-	-	-	-	-	-	-	-	-	-	-	-	-	-	A	-	-	-	V	-	-	-	-	-	-	N	-	-	-	-	-	-	-	-	-	-
		O/BUR/8/1978	-	-	-	-	-	-	-	-	-	-	-	-	-	-	-	-	-	-	-	-	-	-	-	-	-	-	-	-	-	-	-	V	S	-	-	-	-	-	-	-	-	I	T	-	-	-	D	-	I	-	-	-	-	-	-	-	T	-	-	-	-	-	-	-	-	-	-	-	-	-	-	-	-	-	A	-	-	-	V	-	-	-	-	-	-	N	-	-	-	-	-	-	-	-	-	-
		O/BUR/2/1978	-	-	-	T	-	-	-	-	-	-	-	-	-	-	-	-	-	-	-	-	-	-	-	-	-	-	-	-	-	-	-	V	S	-	-	-	-	-	-	-	-	-	T	-	-	-	D	-	I	-	-	-	-	-	-	-	T	-	-	-	-	-	-	-	-	-	-	-	-	-	-	-	-	-	A	-	-	-	V	-	-	-	-	-	-	N	-	-	-	-	-	-	-	-	-	-
		O/LAO/1/1982	-	-	-	T	-	-	-	-	-	-	-	-	-	-	-	-	-	-	-	-	-	-	-	-	-	-	-	-	-	-	-	V	-	-	-	-	-	-	-	-	-	-	T	-	-	-	D	-	I	-	-	-	-	-	-	-	T	-	-	-	-	-	-	-	-	-	-	-	-	-	-	-	-	-	A	-	-	-	V	-	-	-	-	-	-	N	-	-	-	-	-	-	-	-	-	-
		O/LAO/5/1982	-	-	-	T	-	-	-	-	-	-	-	-	-	-	-	-	-	-	-	-	-	-	-	-	-	-	-	-	-	-	-	V	-	-	-	-	-	-	-	-	-	-	T	-	-	-	D	-	I	-	-	-	-	-	-	-	T	-	-	-	-	-	-	-	-	-	-	-	-	-	-	-	-	-	A	-	-	-	V	-	-	-	-	-	-	N	-	-	-	-	-	-	-	-	-	-
		O/BUR/11/1978	-	-	-	T	-	-	-	-	-	-	-	-	-	-	-	-	-	-	-	-	-	-	-	-	-	-	-	-	-	-	-	V	-	-	-	-	-	-	-	-	-	-	T	-	N	-	D	-	I	-	-	-	-	-	-	-	T	-	-	-	-	-	-	-	-	-	-	-	-	-	-	-	-	-	A	-	-	-	V	-	-	-	-	-	-	N	-	-	-	-	-	-	-	-	-	-
	Tai-87	O/MAY/1/1992	-	-	-	T	-	-	-	-	-	-	-	-	-	-	-	-	-	-	-	-	-	-	-	-	-	-	-	H	-	-	-	V	S	-	-	-	-	-	-	-	Q	-	T	-	-	-	D	-	I	-	-	-	-	-	-	-	T	-	-	-	-	-	-	-	-	-	-	-	-	-	-	-	-	-	A	-	-	-	V	-	-	-	-	-	-	N	-	-	-	-	-	-	-	-	-	-
		O/MAY/5/1992	-	-	-	T	-	-	-	-	-	-	-	-	-	-	-	-	-	-	-	-	-	-	-	-	-	-	-	H	-	-	-	V	S	-	-	-	-	-	-	-	Q	-	T	-	-	-	D	-	I	-	-	-	-	-	-	-	T	-	-	-	-	-	-	-	-	-	-	-	-	-	-	-	-	-	A	-	-	-	V	-	-	-	-	-	-	N	-	-	-	-	-	-	-	-	-	-
		O/MAY/5/1993	-	-	-	T	-	-	-	-	-	-	-	-	-	-	-	-	-	-	-	-	-	-	-	-	-	-	-	H	-	-	-	V	S	-	-	-	-	-	-	-	Q	-	T	-	-	-	D	-	I	-	-	-	-	-	-	-	T	-	-	-	-	-	-	-	-	-	-	-	-	-	-	-	-	-	A	-	-	-	V	-	-	-	-	-	-	N	-	-	-	-	-	-	-	-	-	-
		O/LAO/1/1987	-	-	-	T	-	-	-	-	-	-	-	-	-	-	-	-	-	-	-	-	-	-	-	-	-	-	-	H	-	-	-	V	S	-	-	-	-	-	-	-	-	-	T	-	-	-	D	-	I	-	-	-	-	-	-	-	T	-	-	-	-	-	-	-	-	-	-	-	-	-	-	-	-	-	A	-	-	-	V	-	-	-	-	-	-	N	-	-	-	-	-	-	-	-	-	-
		O/LAO/1/1988	-	-	-	T	-	-	-	-	-	-	-	-	-	-	-	-	-	-	-	-	-	-	-	-	-	-	-	H	-	-	-	V	S	-	-	-	-	-	-	-	-	-	T	-	-	-	D	-	I	-	-	-	-	-	-	-	T	-	-	-	-	-	-	-	-	-	-	-	-	-	-	-	-	-	A	-	-	-	V	-	-	-	-	-	-	N	-	-	-	-	-	-	-	-	-	-
		KY091288/O/TAI/189/1987	-	-	-	T	-	-	-	-	-	-	-	-	-	-	-	-	-	-	-	-	-	-	-	-	-	-	-	H	-	-	-	V	S	-	-	-	-	-	-	-	-	-	T	-	-	-	D	-	I	-	-	-	-	-	-	-	T	-	-	-	-	-	-	-	-	-	-	-	-	-	-	-	-	-	A	-	-	-	V	-	-	-	-	K	-	N	-	-	-	-	-	-	-	-	-	-
		HQ116222/O/MYA/5/2002	-	-	-	T	-	-	-	-	-	-	-	-	-	-	-	-	-	-	-	-	-	-	-	-	-	-	-	H	-	-	-	V	S	-	-	-	-	-	-	-	-	-	A	-	-	-	D	-	I	-	-	-	-	-	-	-	T	-	-	-	-	-	-	-	-	-	-	-	-	-	-	-	-	-	A	-	-	-	V	-	-	T	-	-	-	N	-	-	-	-	-	-	-	-	-	-
		O/CAM/1/1989	-	-	-	T	-	-	-	-	-	-	-	-	-	-	-	-	-	-	-	-	-	-	-	G	-	-	-	-	-	-	-	V	T	-	-	-	-	-	-	-	-	-	T	-	Q	-	D	-	I	-	T	-	-	-	-	-	T	-	-	-	-	-	-	-	-	-	-	-	-	-	-	-	-	-	A	-	-	-	V	-	-	-	-	-	-	-	-	-	-	-	-	-	-	-	-	-
		O/CAM/3/1992	-	-	-	T	-	-	-	-	-	-	-	-	-	-	-	-	-	-	-	-	-	-	-	-	-	-	-	H	-	-	-	V	T	-	-	-	-	-	-	-	-	-	T	-	R	-	D	-	I	-	-	-	-	-	-	-	T	-	-	-	-	-	-	-	-	-	-	-	-	-	-	-	-	-	A	-	-	-	V	-	-	-	-	-	-	-	-	-	-	-	-	-	-	-	-	-
		O/TAI/1/1994	-	-	-	T	-	-	-	-	-	-	-	-	-	-	-	-	-	-	-	-	-	-	-	-	-	-	-	-	-	-	-	V	S	-	-	-	-	-	-	-	-	-	T	-	E	-	D	-	M	-	-	-	-	-	-	-	T	-	-	-	-	-	-	-	-	-	-	-	-	-	-	-	-	-	A	-	-	-	V	-	-	-	-	-	-	N	-	-	-	-	-	-	-	-	-	-
	Mya-98	SEA/AJ303536/TAI/4/1999	-	-	-	T	-	-	-	-	-	-	-	-	-	-	-	-	-	-	-	-	-	-	-	-	-	-	-	H	-	-	-	V	S	-	-	-	-	-	-	-	-	-	T	-	-	-	D	-	I	-	-	-	-	-	-	-	T	-	P	-	-	-	-	-	-	-	-	-	-	-	-	-	-	-	A	-	-	-	V	-	-	-	-	-	-	-	-	-	-	-	-	-	-	-	-	-
		SEA/AJ303521/MYA/1/1998	-	-	-	?	-	-	-	-	-	-	-	-	-	?	-	-	-	-	-	-	-	-	-	?	-	-	-	H	-	-	-	V	S	-	-	-	-	-	-	-	-	-	T	-	-	-	D	-	I	-	-	?	-	-	-	-	T	-	P	-	-	-	-	-	-	-	-	-	-	-	-	-	-	-	A	-	-	-	V	-	-	-	-	-	-	-	-	-	-	-	-	-	-	?	-	-
		HQ116240/O/TAI/4/2007	-	-	-	T	-	-	-	-	-	-	-	-	-	-	-	-	-	-	-	-	-	-	-	-	-	-	-	H	-	-	-	V	S	-	-	-	-	-	-	-	-	-	T	-	-	-	D	-	I	-	-	-	-	-	-	-	T	-	S	-	-	-	-	-	-	-	-	-	-	-	-	-	-	-	A	-	-	-	V	-	-	-	-	-	-	-	-	-	-	-	-	-	-	-	-	-
		HQ116241/O/TAI/5/2007	-	-	-	T	-	-	-	-	-	-	-	-	-	-	-	-	-	-	-	-	-	-	-	-	-	-	-	H	-	-	-	V	S	-	-	-	-	-	-	-	-	-	T	-	-	-	D	-	I	-	-	-	-	-	-	-	T	-	S	-	-	-	-	-	-	-	-	-	-	-	-	-	-	-	A	-	-	-	V	-	-	-	-	-	-	-	-	-	-	-	-	-	-	-	-	-
		JQ070302/O/HKN/6/2010	-	-	-	T	-	-	-	-	-	-	-	-	-	-	-	-	-	-	-	-	-	-	-	-	-	-	-	H	-	-	-	V	S	-	-	-	-	-	-	-	-	-	T	-	-	-	D	S	I	-	-	-	-	-	-	-	A	-	P	-	-	-	-	-	-	-	-	-	A	-	-	-	-	-	A	-	-	-	V	-	-	-	-	-	-	-	-	-	-	-	-	-	-	-	-	-
		JQ070301/O/HKN/1/2010	-	-	-	T	-	-	-	-	-	-	-	-	-	-	-	-	-	-	-	-	-	-	-	-	-	-	-	H	-	-	-	V	S	-	-	-	-	-	-	-	-	-	T	-	-	-	D	S	I	-	-	-	-	-	-	-	A	-	P	-	-	-	-	-	-	-	-	-	A	-	-	-	-	-	A	-	-	-	V	-	-	-	-	-	-	-	-	-	-	-	-	-	-	-	-	-
		HQ116209/O/MAY/5/2007	-	-	-	T	-	-	-	-	-	-	-	-	-	-	-	-	-	-	-	-	-	-	-	-	-	-	-	H	-	-	-	V	S	-	-	-	-	-	-	-	-	-	T	-	Q	-	N	-	I	-	-	-	-	-	-	-	T	-	P	-	-	-	-	-	-	-	-	-	-	-	-	-	-	-	A	-	-	-	V	-	-	-	-	-	-	-	-	-	-	-	-	-	-	-	-	-
		HQ116247/O/TAI/12/2007	-	-	-	T	-	-	-	-	-	-	-	-	-	-	-	-	-	-	-	-	-	-	-	-	-	-	-	H	-	-	-	V	S	-	-	-	-	-	-	-	-	-	T	-	Q	-	N	-	I	-	-	-	-	-	-	-	T	-	P	-	-	-	-	-	-	-	-	-	-	-	-	-	-	-	A	-	-	-	V	-	-	-	-	-	-	-	-	-	-	-	-	-	-	-	-	-
		HM055494/O/VN/HB139/2009	-	-	-	T	-	-	-	G	-	-	-	-	-	-	-	-	-	-	-	-	-	-	-	-	-	-	-	H	-	-	-	V	S	-	-	-	-	-	-	-	-	-	T	-	Q	-	D	-	I	-	-	-	-	-	-	-	T	-	P	-	-	-	-	-	-	-	-	-	-	-	-	-	-	-	A	-	-	-	V	-	-	-	-	-	-	-	-	-	-	-	-	-	-	-	-	-
		HM055498/O/VN/HB162/2009	-	-	-	T	-	-	-	-	-	-	-	-	-	-	-	-	-	-	-	-	-	-	-	-	-	-	-	H	-	-	-	V	S	-	-	-	-	-	-	-	-	-	T	-	Q	-	D	-	I	-	-	-	-	-	-	-	T	-	P	-	-	-	-	-	-	-	-	-	-	-	-	-	-	-	A	-	-	-	V	-	-	-	-	-	-	-	-	-	-	-	-	-	-	-	-	-
		HQ116289/O/VIT/6/2006	-	-	M	T	-	-	-	-	-	-	-	-	-	-	-	-	-	-	C	-	-	-	-	-	-	-	-	H	-	-	-	V	S	-	-	-	-	-	-	-	-	-	T	-	R	-	D	-	I	-	-	-	Y	M	-	-	T	-	P	-	-	V	-	-	-	-	-	-	-	-	-	-	-	-	A	-	-	-	V	-	-	-	-	-	-	N	-	-	-	-	-	-	-	-	-	-
	Cam-94	AJ294908/O/CAM/1/1998	-	-	A	V	-	-	-	-	-	-	-	-	-	-	-	-	-	-	-	-	-	-	-	-	-	-	-	-	-	-	-	V	-	-	-	-	-	-	-	-	-	-	T	-	-	-	D	-	I	-	-	-	-	-	-	-	T	-	S	Y	-	-	-	-	-	-	-	-	-	-	-	-	-	-	A	-	-	-	V	-	-	-	-	-	-	N	-	-	-	-	-	-	-	-	-	V
		AJ294909/O/CAM/2/1998	-	-	A	V	-	-	-	-	-	-	-	-	-	-	-	-	-	-	-	-	-	-	-	-	-	-	-	-	-	-	-	V	-	-	-	-	-	-	-	-	-	-	T	-	-	-	D	-	I	-	-	-	-	-	-	-	T	-	S	Y	-	-	-	-	-	-	-	-	-	-	-	-	-	-	A	-	-	-	V	-	-	-	-	-	-	N	-	-	-	-	-	-	-	-	-	V
		AJ294910/O/CAM/3/1998	-	-	A	V	-	-	-	-	-	-	-	-	-	-	-	-	-	-	-	-	-	-	-	-	-	-	-	-	-	-	-	V	-	-	-	-	-	-	-	-	-	-	T	-	-	-	D	-	I	-	-	-	-	-	-	-	T	-	S	Y	-	-	-	-	-	-	-	-	-	-	-	-	-	-	A	-	-	-	V	-	-	-	-	-	-	N	-	-	-	-	-	-	-	-	-	V
		SA/AJ296328/VIT/7/1997	-	-	-	V	-	-	-	-	-	-	-	-	-	-	-	-	-	-	-	-	-	-	-	-	-	-	-	-	-	-	-	V	-	-	-	-	-	-	-	-	-	-	T	-	-	-	G	-	T	-	-	-	-	-	-	-	T	-	S	-	-	-	-	-	-	-	-	-	-	-	-	-	-	-	A	-	-	-	V	-	I	-	-	-	-	N	-	-	-	-	-	-	-	-	-	-
		EU667442/O/LAO/2/1999	-	-	-	-	-	-	-	-	-	-	-	-	-	-	-	-	-	-	-	-	-	-	-	-	-	-	-	-	-	-	-	V	-	-	-	-	-	-	-	-	-	-	T	-	-	-	D	-	I	-	-	-	-	-	-	-	T	-	S	-	-	-	-	-	-	-	-	-	-	-	-	-	-	-	A	-	-	-	V	-	-	-	-	-	-	N	-	-	-	-	-	-	-	-	-	-
		EU667440/O/LAO/1/1998	-	-	-	-	-	-	-	-	-	-	-	-	-	-	-	-	-	-	-	-	-	-	-	-	-	-	-	-	-	-	-	V	-	-	-	-	-	-	-	-	-	-	T	-	-	-	D	-	I	-	-	-	-	-	-	-	T	-	S	-	-	-	-	-	-	-	-	-	-	-	-	-	-	-	A	-	-	-	V	-	-	-	-	-	-	N	-	-	-	-	-	-	-	-	-	-
ME-SA	PanAsia-2	ME-SA/PanAsia-2/ANT-10/KY091282/O/IRN/88/2009	-	-	-	T	-	-	-	-	-	-	-	-	-	-	-	-	-	-	-	-	-	-	-	-	-	-	-	-	-	-	-	V	S	-	-	-	-	-	-	-	-	-	T	-	-	-	D	-	I	-	-	-	-	-	-	-	T	-	-	-	-	-	-	-	-	-	-	-	-	-	-	-	-	-	A	-	-	-	V	-	-	-	-	-	-	N	-	-	-	-	-	-	-	-	-	-
		PA2/KJ831726/PAK/1/2010	-	-	-	T	-	-	-	-	-	-	-	-	-	-	-	-	-	-	-	-	-	-	-	-	-	-	-	-	-	-	-	V	S	-	-	-	-	-	-	-	-	-	T	-	-	-	D	-	I	-	-	-	-	-	-	-	T	-	-	-	-	-	-	-	-	-	-	-	-	-	-	-	-	-	A	-	-	-	V	-	-	-	-	-	-	N	-	-	-	-	-	-	-	-	-	-
		PA2/KR149715/O/IRN/31/2010	-	-	-	T	-	-	-	-	-	-	-	-	-	-	-	-	-	-	-	-	-	-	-	-	-	-	-	-	-	-	-	V	S	-	-	-	-	-	-	-	-	-	T	-	-	-	D	-	I	-	-	-	-	-	-	-	T	-	-	-	-	-	-	-	-	-	-	-	-	-	-	-	-	-	A	-	-	-	V	-	-	-	-	-	-	N	-	-	-	-	-	-	-	-	-	-
		PA2/KR149717/O/UAE/2/2010	-	-	-	T	-	-	-	-	-	-	-	-	-	-	-	-	-	-	-	-	-	-	-	-	-	-	-	-	-	-	-	V	S	-	-	-	-	-	-	-	-	-	T	-	-	-	D	-	I	-	A	-	-	-	-	-	T	-	-	-	-	-	-	-	-	-	-	-	-	-	-	-	-	-	A	-	-	-	V	-	-	-	-	-	-	N	-	-	-	-	-	-	-	-	-	-
		ME-SA/PanAsia-2/KY091281/O/IRN/8/2005	-	-	-	T	-	-	-	-	-	-	-	-	-	-	-	-	-	-	-	-	-	-	-	-	-	-	-	-	-	-	-	V	S	-	-	-	-	-	-	-	-	-	T	-	-	-	D	-	I	-	-	-	-	-	-	-	T	-	-	-	-	-	-	-	-	-	-	-	-	-	-	-	-	-	A	-	-	-	V	-	-	-	-	-	-	N	-	-	-	-	-	-	-	-	-	-
		ME-SA/PanAsia-2/FAR-09/KY091284/O/IRN/31/2009	-	-	-	T	-	-	-	-	-	-	-	-	-	-	-	-	-	-	-	-	-	-	-	-	-	-	-	-	-	-	-	V	S	-	-	-	-	-	-	-	-	-	T	-	-	-	D	-	I	-	-	-	-	-	-	-	T	-	-	-	-	-	-	-	-	-	-	-	-	-	-	-	-	-	A	-	-	-	V	-	-	-	-	-	-	N	-	-	-	-	-	-	-	-	-	-
		ME-SA/PanAsia-2/BAL-09/KY091283/O/IRN/18/2010	-	-	-	T	-	-	-	-	-	-	-	-	-	-	-	-	-	-	-	-	-	-	-	-	-	-	-	-	-	-	-	V	S	-	-	-	-	-	-	-	-	-	T	-	Q	-	D	-	I	-	-	-	-	-	-	-	T	-	-	-	-	-	-	-	-	-	-	-	-	-	-	-	-	-	A	-	-	-	V	-	-	-	-	-	-	N	-	-	-	-	-	-	-	-	-	-
		ME-SA/PanAsia-2/PUN-10/KY091285/O/PAK/16/2010	-	-	-	T	-	-	-	-	-	-	-	-	-	-	-	-	-	-	-	-	-	-	-	-	-	-	-	-	-	-	-	V	S	-	-	-	-	-	-	-	-	-	T	-	-	-	D	-	I	-	-	-	-	-	-	-	T	-	-	-	-	-	-	-	-	-	-	-	-	-	-	-	-	-	A	-	-	-	V	-	-	-	-	-	-	N	-	-	-	-	-	-	-	-	-	-
		PA2/HQ439233/O/KAB/AFG/L643/2009	-	-	-	T	-	-	-	-	-	-	-	-	-	-	-	-	-	-	-	-	-	-	-	-	-	-	-	-	-	-	-	V	S	-	-	-	-	-	-	-	-	-	T	-	-	-	D	-	I	-	-	-	-	-	-	-	T	-	-	-	-	-	-	-	-	-	-	-	-	-	-	-	-	-	A	-	-	-	V	-	-	-	-	-	-	N	-	-	-	-	-	-	-	-	-	-
		PA2/HQ439234/O/BAG/AFG/L1494/2009	-	-	-	T	-	-	-	-	-	-	-	-	-	-	-	-	-	-	-	-	-	-	-	-	-	-	-	-	-	-	-	V	S	-	-	-	-	-	-	-	-	-	T	-	-	-	D	-	I	-	-	-	-	-	-	-	T	-	-	-	-	-	-	-	-	-	-	-	-	-	-	-	-	-	A	-	-	-	V	-	-	-	-	-	-	N	-	-	-	-	-	-	-	-	-	-
		PA2/HQ439232/O/KUN/AFG/L630/2009	-	-	-	T	-	-	-	-	-	-	-	-	-	-	-	-	-	-	-	-	-	-	-	-	-	-	-	-	-	-	-	V	S	-	-	-	-	-	-	-	-	-	T	-	-	-	D	-	I	-	-	-	-	-	-	-	T	-	-	-	-	-	-	-	-	-	-	-	-	-	-	-	-	-	A	-	-	-	V	-	-	-	-	-	-	N	-	-	-	-	-	-	-	-	-	-
	PanAsia	ME-SA/PanAsia/AJ539141/O/UKG/35/2001	-	-	-	-	-	-	-	-	-	-	-	-	-	-	-	-	-	-	-	-	-	-	-	-	-	-	-	-	-	-	-	V	S	-	-	-	-	-	-	-	-	-	T	-	-	-	D	-	I	-	-	-	-	-	-	-	T	-	-	-	-	-	-	-	-	-	-	-	-	-	-	-	-	-	A	-	-	-	V	-	-	-	-	-	-	N	-	-	-	-	-	-	-	-	-	-
		MESA/AJ303499/IRQ/30/2000	-	-	-	T	-	-	-	-	-	-	-	-	-	-	-	-	-	-	-	-	-	-	-	-	-	-	-	-	-	-	-	V	S	-	-	-	-	-	-	-	-	-	T	-	-	-	D	-	I	-	-	-	-	-	-	-	T	-	-	-	-	-	-	-	-	-	-	-	-	-	-	-	-	-	A	-	-	-	V	-	-	-	-	-	-	N	-	-	-	-	-	-	-	-	-	-
		PA/HQ116172/O/CAM/4/2006	-	-	-	T	-	-	-	-	-	-	-	-	-	-	-	-	-	-	-	-	-	-	-	-	-	-	-	-	-	-	-	V	S	-	-	-	-	-	-	-	-	-	T	-	-	-	D	-	I	-	-	-	-	-	-	-	T	-	-	-	-	-	-	-	-	-	-	-	-	-	-	-	-	-	A	-	-	-	V	-	-	-	-	-	-	N	-	-	-	-	-	-	-	-	-	-
		PA/HQ116173/O/CAM/5/2006	-	-	-	T	-	-	-	-	-	-	-	-	-	-	-	-	-	-	-	-	-	-	-	-	-	-	-	-	-	-	-	-	S	-	-	-	-	-	-	-	-	-	T	-	-	-	D	-	I	-	-	-	-	-	-	-	T	-	-	-	-	-	-	-	-	-	-	-	-	-	-	-	-	-	A	-	-	-	V	-	-	-	-	-	-	N	-	-	-	-	-	-	-	-	-	-
		PA/HQ116171/O/CAM/1/2004	-	-	-	T	-	-	-	-	-	-	-	-	-	-	-	-	-	-	-	-	-	-	-	A	-	-	-	-	-	-	-	V	S	-	-	-	-	-	-	-	-	-	T	-	-	-	D	-	I	-	-	-	-	-	-	-	T	-	-	-	-	-	-	-	-	-	-	-	-	-	-	-	-	-	A	-	-	-	V	-	-	-	-	-	-	N	-	-	-	-	-	-	-	-	-	V
		PA/HQ116174/O/CAM/1/2008	-	-	-	T	-	-	-	-	-	-	-	-	-	-	-	-	-	-	-	-	-	-	-	-	-	-	-	-	-	-	-	V	S	-	-	-	-	-	-	-	-	-	T	-	-	-	D	-	I	?	-	-	-	-	-	-	T	-	-	-	-	-	-	-	-	-	-	-	-	-	-	-	-	-	A	-	-	-	V	-	-	-	-	-	-	N	-	-	-	-	-	-	-	-	-	-
		ME-SA/PanAsia/EU667451/O/LAO/2/2003	-	-	-	-	-	-	-	-	-	-	-	-	-	-	-	-	-	-	-	-	-	-	-	-	-	-	-	-	-	-	-	V	S	-	-	-	-	-	-	-	-	-	T	-	-	-	D	-	I	-	-	-	-	-	-	-	T	-	-	-	-	-	-	-	-	-	-	-	-	-	-	-	-	-	A	-	-	-	V	-	-	-	-	-	-	N	-	-	-	-	-	-	-	-	-	-
	Irn-2001	Irn2001/DQ165062/O/PAK/15/2002	-	-	-	-	-	-	-	-	-	-	-	-	-	-	-	-	-	-	-	-	-	-	-	-	-	-	-	-	-	-	-	V	-	-	-	-	-	-	-	-	-	-	T	-	-	-	D	-	I	-	-	-	-	-	-	-	T	-	-	-	-	-	-	-	-	-	-	-	-	-	-	-	-	-	A	-	I	-	V	-	-	-	-	-	-	N	-	-	-	-	-	-	-	-	-	-
		Irn2001/DQ165063/O/PAK/16/2002	-	-	-	-	-	-	-	-	-	-	-	-	-	-	-	-	-	-	-	-	-	-	-	-	-	-	-	-	-	-	-	V	-	-	-	-	-	-	-	-	-	-	T	-	-	-	D	-	I	-	-	-	-	-	-	-	T	-	-	-	-	-	-	-	-	-	-	-	-	-	-	-	-	-	A	-	I	-	V	-	-	-	-	-	-	N	-	-	-	-	-	-	-	-	-	-
		Irn2001/DQ165064/O/PAK/18/2002	-	-	-	-	-	-	-	-	-	-	-	-	-	-	-	-	-	-	-	-	-	-	-	-	-	-	-	-	-	-	-	V	-	-	-	-	-	-	-	-	-	-	T	-	-	-	D	-	I	-	-	-	-	-	-	-	T	-	-	-	-	-	-	-	-	-	-	-	-	-	-	-	-	-	A	-	I	-	V	-	-	-	-	-	-	N	-	-	-	-	-	-	-	-	-	-
		AJ296325/TUR/1/1996	-	-	-	T	-	-	-	-	-	-	-	-	-	-	-	-	-	-	-	-	-	-	-	-	-	-	-	-	-	-	-	V	S	-	-	-	-	-	-	-	-	-	T	-	-	-	D	P	I	T	-	-	-	-	-	-	T	-	-	-	-	-	-	-	?	-	-	-	-	-	-	-	-	-	A	-	-	-	V	-	-	-	-	-	-	N	-	-	-	-	-	-	-	-	-	-
	Ind-2001	ME-SA/Ind-2001a/DQ164904/O/KUW/3/1997	-	-	-	T	-	-	-	-	-	-	-	-	T	-	-	-	-	-	-	-	-	-	-	-	-	-	-	-	-	-	-	V	S	-	-	-	-	-	-	-	-	-	T	-	-	-	D	-	I	-	-	-	-	-	-	-	T	-	-	-	-	-	-	-	-	-	-	-	-	-	-	-	-	-	A	-	-	-	V	-	-	-	-	-	-	N	-	-	-	-	-	-	-	-	-	-
		ME-SA/Ind-2001b/DQ164941/O/OMN/7/2001	-	-	-	T	-	-	-	-	-	-	-	-	T	-	-	-	-	-	-	-	-	-	-	-	-	-	-	-	-	-	-	V	S	-	-	-	-	-	-	-	-	-	T	-	-	-	D	-	I	-	-	-	-	-	-	K	T	-	-	-	-	-	-	-	-	-	-	-	A	-	-	-	-	-	A	-	-	-	V	-	-	-	-	-	-	N	-	-	-	-	-	-	-	-	-	-
		ME-SA/Ind-2001c/KM921876/O/UAE/4/2008	-	-	-	T	-	-	-	-	-	-	-	-	S	-	-	-	-	-	-	-	-	-	-	-	-	-	-	-	-	-	-	V	S	-	-	-	-	-	-	-	-	-	T	-	-	-	D	-	-	-	-	-	-	-	-	-	T	-	-	-	-	-	-	-	-	-	-	-	-	-	-	-	-	-	A	-	-	-	V	-	-	-	-	-	-	N	-	-	-	-	-	-	-	-	-	-
		MF947475/SRL/2/2014	-	-	-	T	-	-	-	-	-	-	-	-	T	-	-	-	-	-	-	-	-	-	-	-	-	-	-	-	-	-	-	V	S	-	-	-	-	-	-	-	-	-	T	-	-	-	D	-	I	-	-	-	-	-	-	-	T	-	-	-	-	-	-	-	-	-	-	-	-	-	-	-	-	-	A	-	-	-	V	-	-	-	-	-	-	N	-	-	-	-	-	-	-	-	-	-
		MF947474/SRL/1/2014	-	-	-	T	-	-	-	-	-	-	-	-	T	-	-	-	-	-	-	-	-	-	-	-	-	-	-	-	-	-	-	V	S	-	-	-	-	-	-	-	-	-	T	-	-	-	D	-	I	-	-	-	-	-	-	-	T	-	-	-	-	-	-	-	-	-	-	-	-	-	-	-	-	-	A	-	-	-	V	-	-	-	-	-	-	N	-	-	-	-	-	-	-	-	-	-
		MF947473/SRL/2/2013	-	-	-	T	-	-	-	-	-	-	-	-	T	-	-	-	-	-	-	-	-	-	-	-	-	-	-	-	-	-	-	V	S	-	-	-	-	-	-	-	-	-	T	-	-	-	D	-	I	-	-	-	-	-	-	-	T	-	-	-	-	-	-	-	-	-	-	-	-	-	-	-	-	-	A	-	-	-	V	-	-	-	-	-	-	N	-	-	-	-	-	-	-	-	-	-
		ME-SA/Ind-2001d/KM921814/O/BHU/3/2009	-	-	-	T	-	-	-	-	-	-	-	-	T	-	-	-	-	-	-	-	-	-	-	-	-	-	-	-	-	-	-	V	S	-	-	-	-	-	-	-	-	-	T	-	-	-	D	-	I	-	-	-	-	-	-	-	T	-	-	-	-	-	-	-	-	-	-	-	-	-	-	-	-	-	A	-	-	-	V	-	-	-	-	-	-	N	-	-	-	-	-	-	-	-	-	-
	Srl-97	MF947468/SRL/13/2010	-	-	-	-	-	-	-	-	-	-	-	-	-	-	-	-	-	-	-	-	-	-	-	-	-	-	-	-	-	-	-	V	S	-	-	-	-	-	-	-	-	-	T	-	-	-	D	-	I	-	-	-	-	-	-	-	T	-	-	-	-	-	-	-	-	-	-	-	-	-	-	-	-	-	A	-	-	-	V	-	-	-	-	-	-	N	-	-	-	-	-	-	-	-	-	-
		MF947467/SRL/9/2010	-	-	-	-	-	-	-	-	-	-	-	-	-	-	-	-	-	-	-	-	-	-	-	-	-	-	-	-	-	-	-	V	S	-	-	-	-	-	-	-	-	-	T	-	-	-	D	-	I	-	-	-	-	-	-	-	T	-	-	-	-	-	-	-	-	-	-	-	-	-	-	-	-	-	A	-	-	-	V	-	-	-	-	-	-	N	-	-	-	-	-	-	-	-	-	-
		KJ831703/SRL/1/2009	-	-	-	-	-	-	-	-	-	-	-	-	-	-	-	-	-	-	-	-	-	-	-	-	-	-	-	-	-	-	-	V	S	-	-	-	-	-	-	-	-	-	T	-	-	-	D	-	I	-	-	-	-	-	-	-	T	-	-	-	-	-	-	-	-	-	-	-	-	-	-	-	-	-	A	-	-	-	V	-	-	-	-	-	-	N	-	-	-	-	-	-	-	-	-	-
		MF947463/SRL/1/2009	-	-	-	-	-	-	-	-	-	-	-	-	-	-	-	-	-	-	-	-	-	-	-	-	-	-	-	-	-	-	-	V	S	-	-	-	-	-	-	-	-	-	T	-	-	-	D	-	I	-	-	-	-	-	-	-	T	-	-	-	-	-	-	-	-	-	-	-	-	-	-	-	-	-	A	-	-	-	V	-	-	-	-	-	-	N	-	-	-	-	-	-	-	-	-	-
		MF947455/SRL/4/1999	-	-	-	T	-	-	-	-	-	-	-	-	-	-	-	-	-	-	-	-	-	-	-	-	-	-	-	-	-	-	-	V	S	-	-	-	-	-	-	-	-	-	T	-	-	-	D	-	-	-	-	-	-	-	-	-	T	-	-	-	-	-	-	-	-	-	-	-	-	-	-	-	-	-	A	-	-	-	V	-	-	-	-	-	-	N	-	-	-	-	-	-	-	-	-	-
		MF947453/SRL/1/1999	-	-	-	T	-	-	-	-	-	-	-	-	-	-	-	-	-	-	-	-	-	-	-	-	-	-	-	-	-	-	-	V	S	-	-	-	-	-	-	-	-	-	T	-	-	-	D	-	-	-	-	-	-	-	-	-	T	-	-	-	-	-	-	-	-	-	-	-	-	-	-	-	-	-	A	-	-	-	V	-	-	-	-	-	-	N	-	-	-	-	-	-	-	-	-	-
		MF947454/SRL/3/1999	-	-	-	T	-	-	-	-	-	-	-	-	-	-	-	-	-	-	-	-	-	-	-	-	-	-	-	-	-	-	-	V	S	-	-	-	-	-	-	-	-	-	T	-	-	-	D	-	-	-	-	-	-	-	-	-	T	-	-	-	-	-	-	-	-	-	-	-	-	-	-	-	-	-	A	-	-	-	V	-	-	-	-	-	-	N	-	-	-	-	-	-	-	-	-	-
		AJ303531/SRL/2/1997	-	-	-	T	-	-	-	-	-	-	-	-	-	-	-	-	-	-	-	-	-	-	-	-	-	-	-	-	-	-	-	V	S	-	-	-	-	-	-	-	-	-	T	-	-	-	D	-	-	-	-	-	-	-	-	-	T	-	-	-	-	-	-	-	-	-	-	-	-	-	-	-	-	-	A	-	-	-	V	-	-	-	-	-	-	N	-	-	-	-	-	-	-	-	-	-
		ME-SA/Srl-97/AJ303531/O/SRL/2/1997	-	-	-	T	-	-	-	-	-	-	-	-	-	-	-	-	-	-	-	-	-	-	-	-	-	-	-	-	-	-	-	V	S	-	-	-	-	-	-	-	-	-	T	-	-	-	D	-	-	-	-	-	-	-	-	-	T	-	-	-	-	-	-	-	-	-	-	-	-	-	-	-	-	-	A	-	-	-	V	-	-	-	-	-	-	N	-	-	-	-	-	-	-	-	-	-
		MF947470/SRL/4/2011	-	-	-	-	-	-	-	-	-	-	-	-	-	-	-	-	-	-	-	-	-	-	-	-	-	-	-	-	-	-	-	V	S	-	-	-	-	-	-	-	-	-	T	-	-	-	D	-	-	-	-	-	-	-	-	-	T	-	-	-	-	-	-	-	-	-	-	-	-	-	-	-	-	-	A	-	-	-	V	-	-	Q	-	K	-	N	-	-	-	-	-	-	-	-	-	-
	Pak-98	Pak98/DQ165068/O/PAK/16/2003	-	-	-	T	-	-	-	-	-	-	-	-	-	-	-	-	-	-	-	-	-	-	-	-	-	-	-	-	-	-	-	V	S	-	-	-	-	-	-	-	-	-	T	-	Q	-	-	-	I	-	-	-	-	-	-	-	T	-	-	-	-	-	-	-	-	-	-	-	-	-	-	-	-	-	A	-	-	-	V	-	-	-	-	-	-	N	-	-	-	-	-	-	-	-	-	-
		Pak98/DQ165070/O/PAK/73/2003	-	-	-	T	-	-	-	-	-	-	-	-	-	-	-	-	-	-	-	-	-	-	-	-	-	-	-	-	-	-	-	V	S	-	-	-	-	-	-	-	-	-	T	-	Q	-	-	-	I	-	-	-	-	-	-	-	T	-	-	-	-	-	-	-	-	-	-	-	-	-	-	-	-	-	A	-	-	-	V	-	-	-	-	-	-	N	-	-	-	-	-	-	-	-	-	-
		Pak98/DQ165069/O/PAK/17/2003	-	-	-	T	-	-	-	-	-	-	-	-	-	-	-	-	-	-	-	-	-	-	-	-	-	-	-	-	-	-	-	V	S	-	-	-	-	-	-	-	-	-	T	-	Q	-	-	-	I	-	-	-	-	-	-	-	T	-	-	-	-	-	-	-	-	-	-	-	-	-	-	-	-	-	A	-	-	-	V	-	-	-	-	-	-	N	-	-	-	-	-	-	-	-	-	?
	Unnamed	ME-SA/unnamed/AF204276/O/IND/R2/1975	-	-	-	T	-	-	-	-	-	-	-	-	-	-	-	-	-	-	-	-	-	-	-	-	-	-	-	-	-	-	-	V	S	-	-	-	-	-	-	-	-	-	T	-	-	-	D	-	I	-	-	-	-	-	-	-	T	-	-	-	-	-	-	-	-	-	-	-	-	-	-	-	-	-	A	-	-	-	V	-	-	-	Y	-	-	N	-	-	-	-	-	-	-	-	-	-
		ME-SA/unnamed/AF292107/O/IND/53/1979	-	-	-	-	-	-	-	-	-	-	-	-	-	-	-	-	-	-	-	-	-	-	-	-	-	-	-	-	-	-	-	V	S	-	-	-	-	-	-	-	-	-	T	-	E	-	D	-	I	-	-	-	-	-	-	-	T	-	-	-	-	-	-	-	-	-	-	-	-	-	-	-	-	-	A	-	-	-	V	-	-	-	-	-	-	N	-	-	-	-	-	-	-	-	-	-
	Tur-69	EU553844/O/ISR/1/1992	-	-	-	V	-	-	-	-	-	-	-	-	-	-	-	-	-	-	-	-	-	-	-	-	-	-	-	-	-	-	-	V	S	-	-	-	-	-	-	-	-	-	T	-	-	-	D	-	I	-	-	-	-	-	-	-	A	-	-	-	-	-	-	-	-	-	-	-	-	-	-	-	-	-	A	-	-	-	V	-	-	-	-	-	-	N	-	-	-	-	-	-	-	-	-	-
		EU553845/O/ITL/1/1993	-	-	-	V	-	-	-	-	-	-	-	-	-	-	-	-	-	-	-	-	-	-	-	-	-	-	-	-	-	-	-	V	S	-	-	-	-	-	-	-	-	-	T	-	-	-	D	-	T	-	-	-	-	-	-	-	A	-	-	-	-	-	-	-	-	-	-	-	-	-	-	-	-	-	A	-	-	-	V	-	-	-	-	-	-	N	-	-	-	-	-	-	-	-	-	-
		EU553846/O/OMN/3/1991	-	-	-	V	-	-	-	-	-	-	-	-	-	-	-	-	-	-	-	-	-	-	-	-	-	-	-	-	-	-	-	V	S	-	-	-	-	-	-	-	-	-	T	-	-	-	D	-	I	-	-	-	-	-	-	-	A	-	-	-	-	-	-	-	-	-	-	-	-	-	-	-	-	-	A	-	-	-	V	-	-	-	-	-	-	N	-	-	-	-	-	-	-	-	-	-
		O/SRL/1/1991	-	-	-	-	-	-	-	-	N	-	-	-	-	-	-	-	-	-	-	-	-	-	-	-	R	-	-	-	-	-	-	V	S	-	-	-	-	-	-	-	-	-	T	-	-	-	D	-	I	-	-	-	-	-	-	-	A	-	-	-	-	-	-	-	-	-	-	-	-	-	-	-	-	-	A	-	-	-	V	-	-	-	-	-	-	N	-	-	-	-	-	-	-	-	-	-

Topotype	Liniage	Isolate	1	2	3	4	5	6	7	8	9	10	11	12	13	14	15	16	17	18	19	20	21	22	23	24	25	26	27	28	29	30	31	32	33	34	35	36	37	38	39	40	41	42	43	44	45	46	47	48	49	50	51	52	53	54	55	56	57	58	59	60	61	62	63	64	65	66	67	68	69	70	71	72	73	74	75	76	77	78	79	80	81	82	83	84	85	86	87	88	89	90	91	92	93	94	95	96
Reference Sequnce		NC 004004 ref seq	T	T	S	A	G	E	S	A	D	P	V	T	A	T	V	E	N	Y	G	G	E	T	Q	V	Q	R	R	Q	H	T	D	I	A	F	I	L	D	R	F	V	K	V	K	P	K	-	E	Q	V	N	V	L	D	L	M	Q	I	P	A	H	T	L	V	G	A	L	L	R	T	A	T	Y	Y	F	S	D	L	E	L	A	V	K	H	E	G	D	L	T	W	V	P	N	G	A	P	E
		ME-SA/unnamed/AY593823/O1/Manisa/TUR/1969	-	-	-	-	-	-	-	-	-	-	-	-	-	-	-	-	-	-	-	-	-	-	-	-	-	-	-	-	-	-	-	V	S	-	-	-	-	-	-	-	-	-	T	-	-	-	D	-	I	-	-	-	-	-	-	-	T	-	-	-	-	-	-	-	-	-	-	-	-	-	-	-	-	-	A	-	-	-	V	-	-	-	-	-	-	N	-	-	-	-	-	-	-	-	-	-
WCSA-1	Srl-77	O/SRL/7/88/1987	-	-	-	S	-	-	-	-	-	-	-	-	-	-	-	-	-	-	-	-	-	-	-	-	-	-	-	-	-	-	-	V	S	-	-	-	-	-	-	-	-	-	E	-	Q	-	T	-	I	T	-	-	-	-	-	-	-	-	-	-	-	-	-	-	-	-	-	-	A	-	-	-	-	-	A	-	-	-	V	-	-	-	-	-	-	N	-	-	-	-	-	-	-	-	-	-
		O/SRL/5/1987	-	-	-	S	-	-	-	-	-	-	-	-	-	-	-	-	-	-	-	-	-	-	-	-	-	-	-	-	-	-	-	V	S	-	-	-	-	-	-	-	-	-	E	-	Q	-	T	-	I	T	-	-	-	-	-	-	-	-	-	-	-	-	-	-	-	-	-	-	A	-	-	-	-	-	A	-	-	-	V	-	-	-	-	-	-	N	-	-	-	-	-	-	-	-	-	-
		O/SRL/4/88/1987	-	-	-	S	-	-	-	-	-	-	-	-	-	-	-	-	-	-	-	-	-	-	-	-	-	-	-	-	-	-	-	V	S	-	-	-	-	-	-	-	-	-	E	-	Q	-	T	-	I	T	-	-	-	-	-	-	-	-	-	-	-	-	-	-	-	-	-	-	A	-	-	-	-	-	A	-	-	-	V	-	-	-	-	-	-	N	-	-	-	-	-	-	-	-	-	-
		O/SRL/3/88/1987	-	-	-	S	-	-	-	-	-	-	-	-	-	-	-	-	-	-	-	-	-	-	-	-	-	-	-	-	-	-	-	V	S	-	-	-	-	-	-	-	-	-	E	-	Q	-	T	-	I	T	-	-	-	-	-	-	-	-	-	-	-	-	-	-	-	-	-	-	A	-	-	-	-	-	A	-	-	-	V	-	-	-	-	-	-	N	-	-	-	-	-	-	-	-	-	-
		O/SRL/6/88/1987	-	-	-	S	-	-	-	-	-	-	-	-	-	-	-	-	-	-	-	-	-	-	-	-	-	-	-	-	-	-	-	V	S	-	-	-	-	-	-	-	-	-	E	-	Q	-	T	-	I	T	-	-	-	-	-	-	-	-	-	-	-	-	-	-	-	-	-	-	A	-	-	-	-	-	A	-	-	-	V	-	-	-	-	-	-	N	-	-	-	-	-	-	-	-	-	-
		O/SRL/9/88/1987	-	-	-	S	-	-	-	-	-	-	-	-	-	-	-	-	-	-	-	-	-	-	-	-	-	-	-	-	-	-	-	V	S	-	-	-	-	-	-	-	-	-	E	-	Q	-	T	-	I	T	-	-	-	-	-	-	-	-	-	-	-	-	-	-	-	-	-	-	A	-	-	-	-	-	A	-	-	-	V	-	-	-	-	-	-	N	-	-	-	-	-	-	-	-	-	-
		O/SRL/2/88/1987	-	-	-	S	-	-	-	-	-	-	-	-	-	-	-	-	-	-	-	-	-	-	-	-	-	-	-	-	-	-	-	V	S	-	-	-	-	-	-	-	-	-	E	-	Q	-	T	-	I	T	-	-	-	-	-	-	-	-	-	-	-	-	-	-	-	-	-	-	A	-	-	-	-	-	A	-	-	-	V	-	-	-	-	-	-	N	-	-	-	-	-	-	-	-	-	-
		O/SRL/1/88/1987	-	-	-	S	-	-	-	-	-	-	-	-	-	-	-	-	-	-	-	-	-	-	-	-	-	-	-	-	-	-	-	V	S	-	-	-	-	-	-	-	-	-	E	-	Q	-	T	-	I	T	-	-	-	-	-	-	-	-	-	-	-	-	-	-	-	-	-	-	A	-	-	-	-	-	A	-	-	-	V	-	-	-	-	-	-	N	-	-	-	-	-	-	-	-	-	-
		O/SRL/8/88/1987	-	-	-	S	-	-	-	-	-	-	-	-	-	-	-	-	-	-	-	-	-	-	-	-	-	-	-	-	-	-	-	V	S	-	-	-	-	-	-	-	-	-	E	-	Q	-	T	-	I	T	-	-	-	-	-	-	-	-	-	-	-	-	-	-	-	-	-	-	A	-	-	-	-	-	A	-	-	-	V	-	-	-	-	-	-	N	-	-	-	-	-	-	-	-	-	-
		O/SRL/10/1988	-	-	-	S	-	-	-	-	-	-	-	-	T	-	-	-	-	-	-	-	-	-	-	-	-	-	-	-	-	-	-	V	S	-	-	-	-	-	-	-	-	-	E	-	Q	-	T	-	I	T	-	-	-	-	-	-	-	-	-	-	-	-	-	-	-	-	-	-	A	-	-	-	-	-	A	-	-	-	V	-	-	-	-	-	-	N	-	-	-	-	-	-	-	-	-	-
		O/SRL/1/1997	-	-	-	S	-	-	-	-	-	-	-	-	-	-	-	-	-	-	-	-	A	-	-	-	-	-	-	-	-	-	-	V	S	-	-	-	-	-	-	-	-	A	V	-	Q	-	P	-	I	T	-	-	-	-	-	-	-	-	-	Q	-	-	-	-	-	-	-	-	-	-	-	-	-	-	A	-	-	-	V	-	-	-	-	-	-	N	-	-	-	-	-	-	-	-	-	-
		O/SRL/1/78/1977	-	-	-	P	-	-	-	-	-	-	-	-	-	-	-	-	-	-	-	-	-	-	-	-	-	-	-	-	-	-	-	V	S	-	-	-	-	-	-	-	-	-	T	-	Q	-	-	-	I	-	-	-	-	-	-	-	-	-	-	-	-	-	-	-	-	-	-	-	-	-	-	-	-	-	A	-	-	-	V	-	-	-	-	-	-	N	-	-	-	-	-	-	-	-	-	-
		O/SRL/3/1978	-	-	-	P	-	-	-	-	-	-	-	-	-	-	-	-	-	-	-	-	-	-	-	-	-	-	-	-	-	-	-	V	S	-	-	-	-	-	-	-	-	-	T	-	Q	-	-	-	I	-	-	-	-	-	-	-	-	-	-	-	-	-	-	-	-	-	-	-	-	-	-	-	-	-	A	-	-	-	V	-	-	-	-	-	-	N	-	-	-	-	-	-	-	-	-	-
		O/SRL/2/1978	-	-	-	P	-	-	-	-	-	-	-	-	-	-	-	-	-	-	-	-	-	-	-	-	-	-	-	-	-	-	-	V	S	-	-	-	-	-	-	-	-	-	T	-	Q	-	-	-	I	-	-	-	-	-	-	-	-	-	-	-	-	-	-	-	-	-	-	-	-	-	-	-	-	-	A	-	-	-	V	-	-	-	-	-	-	N	-	-	-	-	-	-	-	-	-	-
		O/SRL/87/VS/1987	-	-	-	L	-	-	-	-	-	-	-	-	-	-	-	-	-	-	-	-	-	-	-	-	-	-	-	-	-	-	-	V	S	-	-	-	-	-	-	-	-	-	T	-	Q	-	-	-	I	-	-	-	-	-	-	-	-	-	-	-	-	-	-	-	-	-	-	-	-	-	-	-	-	-	A	-	-	-	V	-	-	-	-	K	-	N	-	-	-	-	-	-	-	-	-	-
		O/SRL/3/1987	-	-	-	P	-	-	-	-	-	-	-	-	-	-	-	-	-	-	-	-	-	-	-	-	R	-	-	-	-	-	-	V	S	-	-	-	-	-	-	-	-	-	T	-	Q	-	-	-	I	-	-	-	-	-	-	-	-	-	-	-	-	-	-	-	-	-	-	-	-	-	-	-	-	-	A	-	-	-	V	-	-	-	-	-	-	N	-	-	-	-	-	-	-	-	-	-
	Syr-62	AJ004679/O/1492/Russia/USSR/88/1988	-	-	-	P	-	-	-	-	-	-	-	-	-	-	-	-	-	-	-	-	-	-	-	-	-	-	-	-	-	-	-	V	S	-	-	-	-	-	-	-	-	-	T	-	Q	-	N	-	I	-	-	-	-	-	-	-	T	-	-	-	-	-	-	-	-	-	-	-	-	-	-	-	-	-	A	-	-	-	V	-	-	-	-	-	-	N	-	-	-	-	-	-	-	-	-	-
		O/SYR/18/1962	-	-	-	Q	-	-	-	-	G	-	-	-	S	-	-	-	-	-	-	-	-	-	-	-	-	-	-	H	-	-	-	V	S	-	-	-	-	-	-	-	-	-	T	-	-	-	D	-	I	S	-	-	-	-	-	-	T	-	-	-	-	-	-	-	-	-	-	-	-	-	-	-	-	-	A	-	-	-	V	-	-	-	-	-	-	N	-	-	-	-	-	-	-	-	-	-
		AY593834/O/Rey/IRN/66/1966	-	-	-	P	-	-	-	-	-	-	-	-	-	-	-	-	-	-	-	-	-	-	-	-	-	-	-	-	-	-	-	V	S	-	-	-	-	-	-	-	-	-	T	-	Q	-	D	-	I	-	-	-	-	-	-	-	T	-	-	-	-	-	-	-	-	-	-	-	-	-	-	-	-	-	A	-	-	-	V	-	-	-	-	-	-	N	-	-	-	-	-	-	-	-	-	-
		AJ004663/O/738/Tajikistan/USSR/74/1974	-	-	-	P	-	-	-	-	-	-	-	-	-	-	-	-	-	-	-	-	-	-	-	-	-	-	-	-	-	-	-	V	S	-	-	-	-	-	-	-	-	-	T	-	Q	-	-	K	I	-	-	-	-	-	-	-	T	-	-	-	-	-	-	-	-	-	-	-	A	-	-	-	-	-	A	-	-	-	V	-	-	-	-	-	-	N	-	-	-	-	-	-	-	-	-	-
EA-3		EA-3/unnamed/FJ798109/O/ETH/3/2004	-	-	-	P	-	-	-	-	-	-	-	-	-	-	-	-	-	-	-	-	-	-	-	-	-	-	-	-	-	-	-	V	S	-	-	-	-	-	-	-	-	-	T	-	T	-	N	-	I	-	-	-	-	-	-	-	T	-	-	-	-	-	-	-	-	-	-	-	-	-	-	-	-	-	A	-	-	-	V	-	-	-	-	-	-	N	-	-	-	-	-	-	-	-	-	-
		EA-3/unnamed/FJ798137/O/ETH/1/2007	-	-	-	P	-	-	-	-	-	-	-	-	-	-	-	-	-	-	-	-	-	-	-	-	-	-	-	-	-	-	-	V	S	-	-	-	-	-	-	-	-	-	T	-	Q	-	D	-	I	-	-	-	-	-	-	-	T	-	-	-	-	-	-	-	-	-	-	-	-	S	-	-	-	-	A	-	-	-	V	-	-	-	-	-	-	N	-	-	-	-	-	-	-	-	-	-
		EA-3/unnamed/DQ165075/O/SUD/2/1986	-	-	-	P	-	-	-	-	-	-	-	-	-	-	-	-	-	-	-	-	-	-	-	-	-	-	-	-	-	-	-	V	-	-	-	-	-	-	-	-	-	-	T	-	-	-	D	-	I	-	-	-	-	-	-	-	T	-	-	-	-	-	-	-	-	-	-	-	-	-	-	-	-	-	A	-	-	-	V	-	-	-	-	-	-	N	-	-	-	-	-	-	-	-	-	-
		EA-3/unnamed/FJ798127/O/ETH/2/2006	-	-	-	P	-	-	-	-	-	-	-	-	-	-	-	-	-	-	-	-	-	-	-	-	-	-	-	-	-	-	-	V	S	-	-	-	-	-	-	-	-	-	T	-	-	-	D	-	I	-	-	-	-	-	-	-	-	-	-	-	-	-	-	-	-	-	-	-	-	-	-	-	-	-	A	-	-	-	V	-	-	-	-	D	-	N	-	-	-	-	-	-	-	-	-	-
EA-1		EA/AJ303511/KEN/83/1979	-	-	-	P	-	-	-	-	-	-	-	-	-	-	-	-	-	-	-	-	-	-	-	-	-	-	-	-	-	-	-	V	S	-	-	-	-	-	-	-	-	-	T	-	-	-	D	-	I	-	-	-	-	-	-	-	T	-	-	-	-	-	-	-	T	-	-	-	-	-	-	-	-	-	A	-	I	-	V	-	-	-	-	-	-	N	-	-	-	-	-	-	-	-	-	-
		EA/AJ303514/KEN/2/95/K10/1995	-	-	-	P	-	-	-	-	-	-	-	-	-	-	-	-	-	-	-	-	-	-	-	?	?	-	-	-	-	-	-	?	?	?	?	-	-	-	-	-	-	-	T	-	?	-	D	-	I	-	-	?	-	-	-	-	T	-	-	-	-	-	-	-	-	-	-	?	-	?	-	-	-	-	A	-	I	?	V	-	-	-	-	-	-	N	-	?	G	A	-	-	-	-	-	-
		EA/AJ296327/UGA/5/1996	-	-	-	L	-	-	-	-	-	-	-	-	-	-	-	-	-	-	-	-	-	-	-	-	-	-	-	-	-	-	-	V	S	-	-	-	-	-	-	-	-	-	T	-	L	-	-	G	T	-	-	-	-	-	-	-	T	-	-	-	-	-	-	-	-	-	-	-	-	-	-	-	-	-	A	-	-	-	V	-	-	V	-	D	-	E	-	-	-	-	-	-	-	-	-	Q
EA-2		EA-2/unnamed/DQ165073/O/KEN/5/2002	-	-	-	P	-	-	-	-	-	-	-	-	-	-	-	-	-	-	-	-	A	-	-	-	-	-	-	-	-	-	-	V	S	-	-	-	-	-	-	-	-	-	T	-	Q	-	D	-	I	-	-	-	-	-	-	-	-	-	-	-	-	-	-	-	-	-	-	-	A	S	-	-	-	-	A	-	-	-	V	-	-	-	-	-	-	N	-	-	-	-	-	-	-	-	-	-
		EA-2/unnamed/DQ165077/O/UGA/3/2002	-	-	-	P	-	-	-	-	-	-	-	-	-	-	-	-	-	-	-	-	A	-	-	-	-	-	-	-	-	-	-	V	S	-	-	-	-	-	-	-	-	-	T	-	Q	-	D	-	I	-	-	-	-	-	-	-	-	-	-	-	-	-	-	-	-	-	-	-	A	S	-	-	-	-	A	-	-	-	V	-	-	-	-	-	-	N	-	-	-	-	-	-	-	-	-	-
		EA-2/unnamed/KF561679/O/TAN/2/2004	-	-	-	P	-	-	-	-	-	-	-	-	-	-	-	-	-	-	-	-	V	-	-	N	-	-	-	-	-	-	-	V	S	-	-	-	-	-	-	-	-	-	T	-	Q	-	D	-	-	-	-	-	-	-	-	-	-	-	-	-	-	-	-	-	-	-	-	-	A	S	-	-	-	-	A	-	-	-	V	-	-	-	-	-	-	N	-	-	-	-	-	-	-	-	-	-
		EA-2/unnamed/DQ165074/O/MAL/1/1998	-	-	-	L	-	-	-	-	-	-	-	-	-	-	-	-	-	-	-	-	A	-	-	-	-	-	-	-	-	-	-	V	S	-	-	-	-	-	-	-	-	I	T	-	Q	-	D	-	I	-	-	-	-	-	L	-	-	-	-	-	-	-	-	-	-	-	-	-	A	S	-	-	-	-	A	-	-	-	V	-	-	-	-	-	-	N	-	-	-	-	-	-	-	-	-	-
EA-4		EA-4/unnamed/FJ798141/O/ETH/58/2005	-	-	-	S	-	-	-	-	-	-	-	-	-	-	-	-	-	-	-	-	-	-	-	-	-	-	-	-	-	-	-	V	S	-	-	-	-	-	-	-	-	-	T	-	R	E	N	-	I	-	-	-	-	-	-	-	T	-	-	-	-	-	-	-	-	-	-	-	-	-	-	-	-	-	A	-	-	-	V	-	-	-	-	-	-	N	-	-	-	-	-	-	-	-	-	-
		EA-4/unnamed/HM211075/O/UGA/17/1998	-	-	-	P	-	-	-	-	-	-	-	-	-	-	-	-	-	-	-	-	-	-	-	-	-	-	-	-	-	-	-	V	S	-	-	-	-	-	-	-	-	-	A	-	R	E	N	-	I	-	-	-	-	-	-	-	T	-	-	-	-	-	-	-	-	-	-	-	-	-	-	-	-	-	A	-	-	-	V	-	-	-	-	-	-	N	-	-	-	-	-	-	-	-	-	-
WA		WA/AJ303481/ALG/1/1999	?	-	-	?	-	-	-	-	-	-	-	-	-	-	-	-	-	-	-	-	-	-	-	I	-	-	-	-	-	-	-	V	S	-	-	-	-	-	-	-	?	-	T	-	Q	-	-	-	I	-	-	-	-	-	-	-	T	-	V	-	-	-	-	-	?	-	-	-	-	-	-	-	-	-	A	-	-	-	V	-	-	-	-	-	-	-	-	-	-	-	-	-	-	-	-	-
		WA/AJ303485/CIV/8/1999	-	-	-	P	-	-	-	-	-	-	-	-	-	-	-	-	-	-	-	-	-	-	-	I	-	-	-	-	-	-	-	V	S	-	-	-	-	-	-	-	-	-	T	-	Q	-	-	-	I	-	-	-	-	-	-	-	T	-	-	-	-	-	-	-	-	-	-	-	-	-	-	-	-	-	A	-	-	-	V	-	-	-	-	-	-	-	-	-	-	-	-	-	-	-	-	-
		WA/AJ303488/GHA/5/1993	-	-	-	P	-	-	-	-	-	-	-	-	-	-	-	-	-	-	-	-	-	-	-	T	-	-	-	-	-	-	-	V	S	-	-	-	-	-	-	-	-	-	T	-	Q	-	-	-	I	-	-	-	-	-	-	-	A	-	-	-	-	-	-	-	-	-	-	-	-	-	-	-	-	-	A	-	-	-	V	-	-	-	-	-	-	-	-	-	-	-	-	-	-	-	-	-
Cathay		CATHAY/unnamed/AF308157/O/Yunlin/TAW/1997	-	-	-	-	-	-	-	-	-	-	-	-	-	-	-	-	-	-	-	-	-	-	-	-	-	-	-	-	-	-	-	-	-	-	-	-	-	-	-	-	-	-	-	-	-	-	-	-	-	-	-	-	-	-	-	-	-	-	-	-	-	-	-	-	-	-	-	-	-	-	-	-	-	-	-	-	-	-	-	-	-	-	-	-	-	-	-	-	-	-	-	-	-	-	-	-
		Cathey/AJ296321/TAW/81/1997	-	-	-	-	-	-	-	-	-	-	-	-	-	-	-	-	-	-	-	-	-	-	-	-	-	-	-	-	-	-	-	-	-	-	-	-	-	-	-	-	-	-	-	-	-	-	-	-	-	-	-	-	-	-	-	-	-	-	-	-	-	-	-	-	-	-	-	-	?	-	-	-	-	-	-	-	-	-	-	?	-	-	-	-	-	-	-	-	-	-	-	-	-	-	-	-
		1997/AJ296322/TAW/83/1997	-	-	-	-	-	-	-	-	-	-	-	-	-	-	-	-	-	-	-	-	-	-	-	-	-	-	-	-	-	-	-	-	-	-	-	-	-	-	?	?	-	-	-	-	-	-	-	-	-	-	-	-	-	-	-	-	-	-	-	-	-	-	-	-	-	-	-	-	?	?	-	-	-	-	-	-	-	-	-	-	-	-	-	-	-	-	-	-	-	-	-	-	-	-	-	-
		AJ294923/O/HKN/16/1996	-	-	-	-	-	-	-	-	-	-	-	-	-	-	-	-	-	-	-	-	-	-	-	-	-	-	-	-	-	-	-	-	-	-	-	-	-	-	-	-	-	-	-	-	-	-	-	-	-	-	-	?	-	-	-	-	-	-	-	-	-	-	-	-	-	-	-	-	-	-	-	-	-	-	-	-	-	-	-	-	-	-	-	-	-	?	-	-	-	-	-	-	-	-	-	-
		Cathey/AJ004680/O/1685/Moscow/Russia/1995	-	-	-	-	-	-	-	-	-	-	-	-	-	-	-	-	-	-	-	-	-	-	-	-	-	-	-	-	-	-	-	-	-	-	-	-	-	-	-	-	-	-	-	-	-	-	-	-	-	-	-	-	-	-	-	-	-	-	-	-	-	-	-	-	-	-	-	-	-	-	-	-	-	-	-	-	-	-	-	-	-	-	-	-	-	-	-	-	-	-	-	-	-	-	-	-
		Cathey/AJ294926/O/PHI/7/1996	-	-	-	-	-	-	-	-	-	-	-	-	T	-	-	-	-	-	-	-	-	-	-	-	-	-	-	-	-	-	-	-	-	-	-	-	-	-	-	-	-	-	-	-	-	-	-	-	-	-	-	-	-	-	-	-	-	-	-	-	-	-	-	-	-	-	-	-	-	-	-	-	-	-	-	-	-	-	-	-	-	-	-	-	-	-	-	-	-	-	-	-	-	-	-	-
		CATHAY/unnamed/AJ294919/O/HKN/6/1983	-	-	-	-	-	-	-	-	-	-	-	-	-	-	-	-	-	-	-	-	-	-	-	-	-	-	-	-	-	-	-	-	-	-	-	-	-	-	-	-	-	-	T	-	-	-	D	-	I	-	-	-	-	-	-	-	-	-	-	-	-	-	-	-	-	-	-	-	-	-	-	-	-	-	-	-	-	?	I	-	-	-	-	-	-	-	-	-	-	-	-	-	-	-	-	-
		Cathey/AJ131468/GD/China/1986	-	-	-	-	-	-	-	-	-	-	-	-	-	-	-	-	-	-	-	-	-	-	-	-	-	-	-	-	-	-	-	-	S	-	-	-	-	-	-	-	-	-	T	-	-	-	D	-	I	-	-	-	-	-	-	-	-	-	-	-	-	-	-	R	-	-	-	-	-	-	-	-	-	-	-	-	-	-	-	-	-	-	-	K	-	-	-	-	-	-	-	-	-	-	-	-
		Cathey/AJ294917/O/HKN/14/1982	-	-	-	-	-	-	-	-	-	-	-	-	-	-	-	-	-	-	-	-	-	-	-	-	-	-	-	-	-	-	-	-	S	-	-	-	-	-	-	-	-	-	T	-	-	-	D	-	T	-	-	-	-	-	-	-	-	-	-	-	-	-	-	-	-	-	-	-	-	-	-	-	-	-	-	-	-	-	-	-	-	-	-	-	-	-	-	-	-	-	-	-	-	P	-	-
		CATHAY/unnamed/AJ294911/O/HKN/21/1970	-	-	-	G	-	-	-	-	-	-	-	-	-	-	-	-	-	-	-	-	-	-	-	-	-	-	-	-	-	-	-	V	S	-	-	-	-	-	-	-	-	-	T	-	-	-	D	-	I	-	-	-	-	-	-	-	-	-	-	-	-	-	-	-	-	-	-	-	?	-	-	-	-	-	-	-	-	-	-	-	-	-	-	-	-	-	-	-	-	-	-	-	-	-	-	-
EURO-SA		EURO-SA/O1/AY593815/O1/BFS 1860/UK/1867	-	-	-	-	-	-	-	-	-	-	-	-	T	-	-	-	-	-	-	-	-	-	-	I	-	-	-	-	-	-	-	V	S	-	-	M	-	-	-	-	-	-	T	-	Q	-	N	-	I	-	I	-	-	-	-	-	V	-	S	-	-	-	-	-	-	-	-	-	A	S	-	-	-	-	-	-	-	-	I	-	-	-	-	-	-	-	-	-	-	-	-	-	-	-	-	-
		EuroSA/K01201/O1Campos/Brazil/1971	-	-	-	-	-	-	-	-	-	-	-	-	-	-	-	-	-	-	-	-	-	-	-	I	-	-	-	-	-	-	-	V	S	-	-	M	-	-	-	-	-	-	T	-	Q	-	N	-	I	-	I	-	-	-	-	-	-	-	S	-	-	-	-	-	-	-	-	-	A	S	-	-	-	-	-	-	-	-	I	-	-	-	-	-	-	-	-	-	-	-	-	-	-	-	-	-
		EuroSA/X00871/O1/Kaufbeuren/FRG/1966	-	-	-	-	-	-	-	-	-	-	-	-	T	-	-	-	-	-	-	-	-	-	-	I	-	-	-	-	-	-	-	V	S	-	-	M	-	-	-	-	-	-	T	-	Q	-	N	-	I	-	I	-	-	-	-	-	-	-	S	-	-	-	-	-	-	-	-	-	A	S	-	-	-	-	-	-	-	-	I	-	-	-	-	-	-	-	-	-	-	-	-	-	-	-	-	-
		EuroSA/Z21862/O/Yrigoyen/ARG/1982	-	-	-	M	-	-	-	-	-	-	-	-	-	-	-	-	-	-	-	-	D	-	-	-	-	-	-	-	-	-	-	V	S	-	-	M	-	-	-	-	-	-	T	-	Q	-	S	S	I	-	T	-	-	-	-	-	-	-	S	-	-	-	-	-	-	-	-	-	A	S	-	-	-	-	-	-	-	-	I	V	-	-	-	-	-	-	-	-	-	-	-	-	-	-	-	V
		EuroSA/M89900/O1/Caseros/ARG/1967	?	?	?	?	?	?	-	T	?	-	-	-	-	-	-	-	-	-	-	-	D	-	-	-	-	-	-	-	-	-	-	V	S	-	-	M	-	-	-	-	-	-	T	-	Q	-	S	S	I	-	T	-	-	-	-	-	-	Q	S	-	-	-	-	?	?	?	?	?	A	S	-	-	-	-	-	-	-	-	I	V	-	-	-	-	-	-	-	-	-	-	R	-	-	-	?	?
		Eurosa/M58601/O1/Lombardy/ITL/1946	-	-	-	-	-	-	-	-	-	-	-	A	-	-	-	-	-	-	-	-	-	-	-	-	-	-	-	-	-	-	-	V	?	-	-	M	-	-	-	-	-	-	T	-	-	-	D	-	T	-	-	-	-	-	-	-	-	-	S	Y	-	-	-	-	-	-	-	-	A	S	-	-	-	-	-	-	-	-	I	-	-	-	-	-	-	Y	-	-	-	-	-	-	-	-	-	-
		EuroSA/M55287/O2/Brescia/ITL/1947	-	-	-	-	-	-	-	-	-	-	-	-	-	-	-	-	S	-	-	-	-	-	-	-	-	-	-	-	-	-	-	V	S	-	-	M	-	-	-	-	-	-	T	-	T	-	D	-	T	-	-	-	N	-	-	-	-	-	S	-	-	-	-	-	-	-	-	-	A	S	-	-	-	-	-	-	-	-	I	-	-	-	-	-	-	-	-	-	-	-	-	-	-	-	-	-
		EuroSA/AJ004645/O3/Venezuela/1951	-	-	-	-	-	-	-	-	-	-	-	-	S	-	-	-	-	-	-	-	-	-	-	-	-	-	-	-	-	-	-	V	S	-	-	M	-	-	-	-	-	-	T	-	E	-	D	-	I	-	-	-	-	-	-	-	-	-	G	-	-	-	-	-	-	-	-	-	A	S	-	-	-	-	-	-	-	-	I	-	-	-	-	-	-	-	-	-	-	-	-	-	-	-	-	-
		EURO-SA/unnamed/DQ834727/O/Corrientes/ARG/2006	-	-	-	-	-	-	-	-	-	-	-	-	S	-	-	-	-	-	-	-	-	-	-	-	-	-	-	H	-	-	-	V	-	-	-	-	-	-	-	A	-	-	T	-	-	-	D	-	I	-	-	-	-	-	-	-	-	-	P	-	-	-	-	-	-	-	-	-	A	S	-	-	-	-	-	-	M	-	I	V	-	-	-	-	-	N	-	-	-	-	-	-	-	-	-	-
CEY-1		O/CEY/1/71/1962	-	-	-	-	-	-	-	-	-	-	-	-	-	-	-	-	-	-	-	-	-	-	-	-	-	-	-	-	-	-	-	V	-	-	-	-	-	-	-	-	-	-	T	-	T	E	G	-	I	-	-	-	-	-	-	-	-	-	S	-	-	-	-	-	-	-	-	-	A	-	-	-	-	-	-	-	-	-	-	-	-	-	-	-	-	N	-	-	-	-	-	-	-	-	-	-
		O/SRL/1/76/1962	-	-	-	-	-	-	-	-	-	-	-	-	-	-	-	-	-	-	-	-	-	-	-	-	-	-	-	-	-	-	-	V	-	-	-	-	-	-	-	-	-	-	T	-	T	E	G	-	I	-	-	-	-	-	-	-	-	-	S	-	-	-	-	-	-	-	-	-	A	-	-	-	-	-	-	-	-	-	-	-	-	-	-	-	-	N	-	-	-	-	-	-	-	-	-	-
		O/SRL/1/1982	-	-	-	-	-	-	-	-	-	-	-	-	-	-	-	-	-	-	-	-	-	-	-	-	-	-	-	-	-	-	-	V	S	-	-	-	-	-	-	-	-	-	T	-	-	Q	D	-	I	-	-	-	-	-	-	-	-	-	S	-	-	-	-	-	-	-	-	-	A	-	-	-	-	-	-	-	-	-	-	-	-	-	-	-	-	N	-	-	-	-	-	-	-	-	-	-
ISA-2		ISA2/AJ303501/ISA/1/1974	-	-	-	-	-	-	-	-	-	-	-	-	-	-	-	-	-	H	-	-	-	-	-	-	R	-	-	-	-	-	-	V	S	-	-	-	-	-	-	-	-	-	T	-	-	A	D	-	I	-	-	-	-	-	-	-	-	-	-	-	-	-	-	-	G	-	V	-	A	-	-	-	-	-	-	-	-	-	I	-	-	-	-	-	-	-	-	-	-	-	-	-	-	-	-	-
		ISA2/AJ303509/JAV/5/1972	-	-	-	-	-	-	-	-	-	-	-	-	-	-	-	-	-	-	-	-	-	-	-	-	R	-	-	-	-	-	-	V	S	-	-	-	-	-	-	-	-	-	T	-	-	A	D	-	I	-	-	-	-	-	-	-	-	-	-	-	-	-	-	-	G	-	V	-	A	-	-	-	-	-	-	-	-	-	-	-	-	-	-	-	-	N	-	-	-	-	-	-	-	-	-	-
ISA-1		ISA1/AJ303503/ISA/8/1983	-	-	-	-	-	-	-	-	-	-	-	-	-	-	-	-	-	-	-	-	-	-	-	-	-	-	-	-	-	-	-	V	S	-	-	-	-	-	-	-	-	-	T	-	T	N	N	-	I	-	-	-	-	-	-	-	-	-	-	-	-	-	-	-	-	-	-	-	A	-	-	-	-	-	-	-	-	-	-	-	-	V	-	-	-	N	-	-	-	-	-	-	-	-	-	-
		ISA1/AJ303502/ISA/9/1974	-	-	-	T	-	-	-	-	-	-	-	-	-	-	-	-	-	-	-	-	-	-	-	-	-	-	-	-	-	-	-	V	S	-	-	-	-	-	-	-	-	-	T	-	N	T	N	-	I	-	-	-	-	-	-	-	-	-	-	-	-	-	-	-	-	-	-	-	-	-	-	-	-	-	-	-	-	-	-	-	-	A	-	-	-	N	-	-	-	-	-	-	-	-	-	-
		ISA1/AJ303500/ISA/1/1962	-	-	-	T	-	-	-	-	-	-	-	-	-	-	-	-	-	-	-	-	-	-	-	-	-	-	-	-	-	-	-	V	S	-	-	-	-	-	-	-	-	-	T	-	Q	N	D	-	I	-	-	-	-	-	-	-	-	-	-	-	-	-	-	-	-	-	-	-	-	-	-	-	-	-	-	-	-	-	-	-	-	A	-	-	-	N	-	-	-	-	-	-	-	-	-	-

Topotype	Liniage	Isolate	97	98	99	100	101	102	103	104	105	106	107	108	109	110	111	112	113	114	115	116	117	118	119	120	121	122	123	124	125	126	127	128	129	130	131	132	133	134	135	136	137	138	139	140	141	142	143	144	145	146	147	148	149	150	151	152	153	154	155	156	157	158	159	160	161	162	163	164	165	166	167	168	169
Reference Sequnce		NC 004004 ref seq	T	A	L	D	N	T	T	N	P	T	A	Y	H	K	E	P	L	T	R	L	A	L	P	Y	T	A	P	H	R	V	L	A	T	V	Y	N	G	S	S	K	Y	G	D	T	S	T	N	N	V	R	G	D	L	Q	V	L	A	Q	K	A	E	R	T	L	P	T	S	F	N	F	G	A	I
SEA	May-78	O/MAY/1/1980	-	-	-	-	-	-	-	-	-	-	-	-	-	-	A	-	-	-	-	-	-	-	-	-	-	-	-	-	-	-	-	-	-	-	-	-	-	N	C	-	-	A	E	G	-	L	T	-	-	-	-	-	-	-	-	-	-	-	-	-	A	-	P	-	-	-	-	-	-	Y	-	-	-
		O/TAI/2/1980	-	-	-	-	-	-	-	-	-	-	-	-	-	-	A	-	-	-	-	-	-	-	-	-	-	-	-	-	-	-	-	-	-	-	-	-	-	N	C	-	-	A	E	G	-	L	T	-	-	-	-	-	-	-	-	-	-	-	-	-	A	-	P	-	-	-	-	-	-	Y	-	-	-
		O/MAY/9/1980	-	-	-	-	-	-	-	-	-	-	-	-	-	-	A	-	-	-	-	-	-	-	-	-	-	-	-	-	-	-	-	-	-	-	-	-	-	N	C	-	-	A	E	G	-	L	T	-	-	-	-	-	-	-	-	-	-	-	-	-	A	-	P	-	-	-	-	-	-	Y	-	-	-
		O/TAI/3/1980	-	-	-	-	-	-	-	-	-	-	-	-	-	-	A	-	-	-	-	-	-	-	-	-	-	-	-	-	-	-	-	-	-	-	-	-	-	N	C	-	-	A	E	G	-	L	T	-	-	-	-	-	-	-	-	-	-	-	-	-	A	-	P	-	-	-	-	-	-	Y	-	-	-
		O/MAY/11/1980	-	-	-	-	-	-	-	-	-	-	-	-	-	-	A	-	-	-	-	-	-	-	-	-	-	-	-	-	-	-	-	-	-	-	-	-	-	N	C	-	-	A	E	G	-	L	T	-	-	-	-	-	-	-	-	-	-	-	-	-	A	-	P	-	-	-	-	-	-	Y	-	-	-
		O/TAI/1/1980	-	-	-	-	-	-	-	-	-	-	-	-	-	-	A	-	-	-	-	-	-	-	-	-	-	-	-	-	-	-	-	-	-	-	-	-	-	N	C	-	-	A	E	G	-	L	T	-	-	-	-	-	-	-	-	-	-	-	-	-	A	-	P	-	-	-	-	-	-	Y	-	-	-
		O/MAY/7/1981	-	-	-	-	-	-	-	-	-	-	-	-	-	-	A	-	-	-	-	-	-	-	-	-	-	-	-	-	-	-	-	-	-	-	-	-	-	N	C	-	-	A	E	G	-	L	T	-	-	-	-	-	-	-	-	-	-	-	-	-	A	-	P	-	-	-	-	-	-	Y	-	-	-
		O/MAY/16/1981	-	-	-	-	-	-	-	-	-	-	-	-	-	-	A	-	-	-	-	-	-	-	-	-	-	-	-	-	-	-	-	-	-	-	-	-	-	N	C	-	-	A	E	G	-	L	T	-	-	-	-	-	-	-	-	-	-	-	-	-	A	-	P	-	-	-	-	-	-	Y	-	-	-
		O/LAO/2/1981	-	-	-	-	-	-	-	-	-	-	-	-	-	-	A	-	-	-	-	-	-	-	-	-	-	-	-	-	-	-	-	-	-	-	-	-	-	N	C	-	-	A	E	G	-	L	T	-	-	-	-	-	-	-	-	-	-	-	-	-	A	-	P	-	-	-	-	-	-	Y	-	-	-
		O/HKN/17/82/AJ294918/1982	-	-	-	-	-	-	-	-	-	-	-	-	-	-	A	Q	-	-	-	-	-	-	-	-	-	-	-	-	-	-	-	-	-	-	-	-	-	N	C	-	-	A	E	G	-	L	T	-	-	-	-	-	-	-	-	-	-	-	-	-	A	-	P	-	-	-	-	-	-	Y	-	-	-
		O/MAY/19/1978	-	-	-	-	-	-	-	-	-	-	-	-	-	-	A	-	-	-	-	-	-	-	-	-	-	-	-	-	-	-	-	-	-	-	-	-	-	N	C	-	-	A	E	G	-	L	T	-	-	-	-	-	-	-	-	-	-	-	-	-	A	-	P	-	-	-	-	-	-	Y	-	-	-
		O/MAY/24/1978	-	-	-	-	-	-	-	-	-	-	-	-	-	-	A	-	-	-	-	-	-	-	-	-	-	-	-	-	-	-	-	-	-	-	-	-	-	N	C	-	-	A	E	G	-	L	T	-	-	-	-	-	-	-	-	-	-	-	-	-	A	-	P	-	-	-	-	-	-	Y	-	-	-
		O/MAY/1/1978	-	-	-	-	-	-	-	-	-	-	-	-	-	-	A	-	-	-	-	-	-	-	-	-	-	-	-	-	-	-	-	-	-	-	-	-	-	N	C	-	-	A	E	G	-	L	T	S	-	-	-	-	-	-	-	-	-	-	-	-	A	-	P	-	-	-	-	-	-	Y	-	-	-
		O/MAY/3/1979	-	-	-	-	-	-	-	-	-	-	-	-	-	-	A	-	-	-	-	-	-	-	-	-	-	-	-	-	-	-	-	-	-	-	-	-	-	N	C	-	-	A	E	G	-	L	T	-	-	-	-	-	-	-	-	-	-	-	-	-	A	-	P	-	-	-	-	-	-	Y	-	-	-
		O/MAY/1/1984	-	-	-	-	-	-	-	-	-	-	-	-	-	-	A	-	-	-	-	-	-	-	-	-	-	-	-	-	-	-	-	-	-	-	-	-	-	N	C	-	-	A	E	G	-	L	T	H	-	-	-	-	-	-	-	-	-	R	-	-	A	-	P	-	-	-	-	-	-	Y	-	-	-
		O/SRL/3/1984	-	-	-	-	-	-	-	-	-	-	-	-	-	-	A	-	-	-	-	-	-	-	-	-	-	-	-	-	-	-	-	-	-	-	-	-	-	N	C	-	-	A	V	G	-	L	T	-	-	-	-	-	-	-	-	-	-	-	-	-	A	-	P	-	-	-	-	-	-	Y	-	-	-
		O/SRL/2/1984	-	-	-	-	-	-	-	-	-	-	-	-	-	-	A	-	-	-	-	-	-	-	-	-	-	-	-	-	-	-	-	-	-	-	-	-	-	N	C	-	-	A	E	G	-	L	T	-	-	-	-	-	-	-	-	-	-	-	-	-	A	-	P	-	-	-	-	-	-	Y	-	-	-
		O/MAY/3/1981	-	-	-	-	-	-	-	-	-	-	-	-	-	-	A	-	-	-	-	-	-	-	-	-	-	-	-	-	-	-	-	-	-	I	-	-	-	N	C	-	-	A	E	G	P	S	T	G	-	-	-	-	-	-	-	-	-	-	-	-	A	-	P	-	-	-	-	-	-	Y	-	-	-
		O/MAY/10/1994	-	-	-	E	-	-	-	-	-	-	-	-	-	-	A	-	-	-	-	-	-	-	-	-	-	-	-	-	-	-	-	-	-	-	-	-	-	N	C	-	-	A	E	G	-	P	T	-	-	-	-	-	-	-	-	-	-	-	-	-	A	-	P	-	-	-	-	-	-	Y	-	-	-
		O/TAI/6/1995	-	-	-	-	-	-	-	-	-	-	-	-	-	-	A	-	-	-	-	-	-	-	-	-	-	-	-	-	-	-	-	-	-	-	-	-	-	N	C	-	-	A	E	S	-	P	T	-	-	-	-	-	-	-	-	-	-	-	-	-	A	-	P	-	-	-	-	-	-	Y	-	-	-
		O/TAI/2/2000/DQ164979	-	-	?	-	-	-	-	-	-	-	-	-	-	-	A	?	-	-	-	-	-	-	-	-	-	-	-	-	-	-	-	-	-	-	-	-	-	N	C	-	-	A	E	S	-	R	T	-	-	-	-	-	-	-	-	-	-	-	R	-	A	-	S	-	-	-	-	-	-	Y	-	-	-
	Bur-77	O/BUR/2/1977	-	-	-	-	-	-	-	-	-	-	-	-	-	-	A	-	-	-	-	-	-	-	-	-	-	-	-	-	-	-	-	-	-	-	-	-	-	N	C	-	-	A	E	G	P	L	T	-	-	-	-	-	-	-	-	-	-	-	-	-	A	-	P	-	-	-	-	-	-	Y	-	-	-
		O/BUR/8/1978	-	-	-	-	-	-	-	-	-	-	-	-	-	-	A	-	-	-	-	-	-	-	-	-	-	-	-	-	-	-	-	-	-	-	-	-	-	N	C	-	-	A	E	G	-	L	T	-	-	-	-	-	-	-	-	-	-	-	-	-	A	-	P	-	-	-	-	-	-	Y	-	-	-
		O/BUR/2/1978	-	-	-	-	-	-	-	-	-	-	-	-	-	-	A	-	-	-	-	-	-	-	-	-	-	-	-	-	-	-	-	-	-	-	-	-	-	N	C	-	-	A	A	G	-	L	T	-	-	-	-	-	-	-	-	-	-	-	-	-	A	-	A	-	-	-	-	-	-	Y	-	-	-
		O/LAO/1/1982	A	-	-	-	-	-	-	-	-	-	-	-	-	-	A	-	-	-	-	-	-	-	-	-	-	-	-	-	-	-	-	-	-	-	-	-	-	N	C	-	-	A	Q	S	P	P	A	-	-	-	-	-	-	-	-	-	-	-	-	-	A	-	P	-	-	-	-	-	-	Y	-	-	-
		O/LAO/5/1982	A	-	-	-	-	-	-	-	-	-	-	-	-	-	A	-	-	-	-	-	-	-	-	-	-	-	-	-	-	-	-	-	-	-	-	-	-	N	C	-	-	A	Q	S	P	P	A	-	-	-	-	-	-	-	-	-	-	-	-	-	A	-	P	-	-	-	-	-	-	Y	-	-	-
		O/BUR/11/1978	-	-	-	-	-	-	-	-	-	-	-	-	-	-	A	-	-	-	-	-	-	-	-	-	-	-	-	-	-	-	-	-	-	-	-	-	-	N	C	-	-	A	Q	G	P	L	A	-	-	-	-	-	-	-	-	-	-	-	-	-	A	K	A	-	-	-	-	-	-	Y	-	-	-
	Tai-87	O/MAY/1/1992	-	-	-	-	-	A	-	-	-	-	-	-	-	-	A	-	-	-	-	-	-	-	-	-	-	-	-	-	-	-	-	-	-	-	-	-	-	N	C	-	-	A	E	R	-	L	T	-	-	-	-	-	-	-	-	-	-	-	-	-	A	-	P	-	-	-	-	-	-	Y	-	-	-
		O/MAY/5/1992	-	-	-	-	-	A	-	-	-	-	-	-	-	-	A	-	-	-	-	-	-	-	-	-	-	-	-	-	-	-	-	-	-	-	-	-	-	N	C	-	-	A	E	R	-	L	T	-	-	-	-	-	-	-	-	-	-	-	-	-	A	-	P	-	-	-	-	-	-	Y	-	-	-
		O/MAY/5/1993	-	-	-	-	-	A	-	-	-	-	-	-	-	-	A	-	-	-	-	-	-	-	-	-	-	-	-	-	-	-	-	-	-	-	-	-	-	N	C	-	-	A	E	R	-	L	T	-	-	-	-	-	-	-	-	-	-	-	-	-	A	-	P	-	-	-	-	-	-	Y	-	-	-
		O/LAO/1/1987	-	-	-	-	-	A	-	-	-	-	-	-	-	-	A	-	-	-	-	-	-	-	-	-	-	-	-	-	-	-	-	-	-	-	-	-	-	N	C	-	-	A	E	G	-	L	T	-	-	-	-	-	-	-	-	-	-	-	-	-	A	-	P	-	-	-	-	-	-	Y	-	-	-
		O/LAO/1/1988	-	-	-	-	-	A	-	-	-	-	-	-	-	-	A	-	-	-	-	-	-	-	-	-	-	-	-	-	-	-	-	-	-	-	-	-	-	N	C	-	-	A	E	G	-	L	T	-	-	-	-	-	-	-	-	-	-	-	-	-	A	-	P	-	-	-	-	-	-	Y	-	-	-
		KY091288/O/TAI/189/1987	-	-	-	-	-	A	-	-	-	-	-	-	-	-	A	-	-	-	-	-	-	-	-	-	-	-	-	-	-	-	-	-	-	-	-	-	-	N	C	-	-	A	E	G	-	L	T	-	-	-	-	-	-	-	-	-	-	-	-	-	A	-	P	-	-	-	-	-	-	Y	-	-	-
		HQ116222/O/MYA/5/2002	-	-	-	-	-	A	-	-	-	-	-	-	-	-	A	-	-	-	-	-	-	-	-	-	-	-	-	-	-	-	-	-	-	-	-	-	-	N	C	-	-	A	E	R	-	P	T	-	-	-	-	-	-	-	-	-	-	-	-	-	A	-	P	-	-	-	-	-	-	Y	-	-	-
		O/CAM/1/1989	A	-	-	-	-	-	-	-	-	-	-	-	-	-	A	-	-	-	-	-	-	-	-	-	-	-	-	-	-	-	-	-	-	-	-	-	-	N	C	-	-	A	E	G	-	L	T	-	-	-	-	-	-	-	-	-	-	-	-	-	A	-	P	-	-	-	-	-	-	Y	-	-	-
		O/CAM/3/1992	A	-	-	-	-	-	-	-	-	-	-	-	-	-	A	-	-	-	-	-	-	-	-	-	-	-	-	-	-	-	-	-	-	-	-	-	-	N	C	-	-	A	E	G	-	L	T	-	-	-	-	-	-	-	-	-	-	-	-	-	A	-	S	-	-	-	-	-	-	Y	-	-	-
		O/TAI/1/1994	-	-	-	-	-	A	-	-	-	-	-	-	-	-	A	-	-	-	-	-	-	-	-	-	-	-	-	-	-	-	-	-	-	-	-	-	-	N	C	-	-	T	N	E	P	L	T	-	-	-	-	-	-	-	-	-	-	-	-	-	A	-	P	-	-	-	-	-	-	Y	-	-	-
	Mya-98	SEA/AJ303536/TAI/4/1999	A	-	-	N	-	-	-	-	-	-	-	-	-	-	A	-	-	-	-	-	-	-	-	-	-	-	-	-	-	-	-	-	-	-	-	-	-	N	C	-	-	A	E	G	-	L	T	-	-	-	-	-	-	-	-	-	-	-	-	-	A	-	P	-	-	-	-	-	-	Y	-	-	-
		SEA/AJ303521/MYA/1/1998	A	-	-	N	-	-	-	-	-	-	-	-	-	-	A	?	-	-	-	-	-	-	-	-	-	-	-	-	-	-	-	-	-	-	-	?	-	N	C	?	-	A	E	G	-	L	T	-	-	-	-	-	-	-	-	-	-	-	-	-	A	-	P	-	-	-	-	-	-	Y	-	-	-
		HQ116240/O/TAI/4/2007	-	-	-	-	-	-	-	-	-	-	-	-	-	-	A	-	-	-	-	-	-	-	-	-	-	-	-	-	-	-	-	-	-	-	-	-	-	N	C	-	-	A	E	G	-	L	A	-	-	-	-	-	-	-	-	-	-	-	-	-	A	-	P	-	-	-	-	-	-	Y	-	-	-
		HQ116241/O/TAI/5/2007	-	-	-	-	-	-	-	-	-	-	-	-	-	-	A	-	-	-	-	-	-	-	-	-	-	-	-	-	-	-	-	-	-	-	-	-	-	N	C	-	-	A	E	G	-	L	A	-	-	-	-	-	-	-	-	-	-	-	-	-	A	-	P	-	-	-	-	-	-	Y	-	-	-
		JQ070302/O/HKN/6/2010	A	-	-	-	-	-	-	-	-	-	-	-	-	-	A	-	-	-	-	-	-	-	-	-	-	-	-	-	-	-	-	-	-	-	-	-	-	N	Y	-	-	A	G	G	-	L	T	-	-	-	-	-	-	-	-	-	-	-	-	-	A	-	P	-	-	-	-	-	-	Y	-	-	-
		JQ070301/O/HKN/1/2010	A	-	-	-	-	-	-	-	-	-	-	-	-	-	A	-	-	-	-	-	-	-	-	-	-	-	-	-	-	-	-	-	-	-	-	-	-	N	Y	-	-	A	G	G	-	L	T	-	-	-	-	-	-	-	-	-	-	-	-	-	A	-	P	-	-	-	-	-	-	Y	-	-	-
		HQ116209/O/MAY/5/2007	A	-	-	G	-	-	-	-	-	-	-	-	-	-	A	-	-	-	-	-	-	-	-	-	-	-	-	-	-	-	-	-	-	A	-	-	-	N	C	-	-	A	G	G	P	L	T	-	-	-	-	-	-	-	-	-	-	-	-	-	A	-	P	-	-	-	-	-	-	Y	-	-	-
		HQ116247/O/TAI/12/2007	A	-	-	-	-	-	-	-	-	-	-	-	-	-	A	-	-	-	-	-	-	-	-	-	-	-	-	-	-	-	-	-	-	-	-	-	-	N	C	-	-	A	G	G	P	L	T	-	-	-	-	-	-	-	-	-	-	-	-	-	A	-	P	-	-	-	-	-	-	Y	-	-	-
		HM055494/O/VN/HB139/2009	A	-	-	G	-	-	-	-	-	-	-	-	-	-	A	-	-	-	-	-	-	-	-	-	-	-	-	-	-	-	-	-	-	-	-	-	-	N	C	-	-	A	G	G	P	L	T	-	-	-	-	-	-	-	-	-	-	-	-	-	A	-	P	-	-	-	-	-	-	Y	-	-	-
		HM055498/O/VN/HB162/2009	A	-	-	G	-	-	-	-	-	-	-	-	-	-	A	-	-	-	-	-	-	-	-	-	-	-	-	-	A	-	-	-	-	-	-	-	-	N	C	-	-	A	G	G	P	L	T	-	-	-	-	-	-	-	-	-	-	-	-	-	A	-	P	-	-	-	-	-	-	Y	-	-	-
		HQ116289/O/VIT/6/2006	A	-	-	E	H	-	-	-	-	-	-	-	-	-	A	-	-	-	-	-	-	-	-	-	-	-	-	-	-	-	-	-	-	-	-	-	-	N	C	-	-	A	E	G	P	L	T	-	-	-	-	-	-	-	-	-	-	-	E	-	A	-	P	-	-	-	-	-	-	Y	-	-	-
	Cam-94	AJ294908/O/CAM/1/1998	-	-	-	-	-	-	-	-	-	-	-	-	-	-	A	?	-	-	-	-	-	-	-	-	-	-	-	-	-	-	-	-	-	-	-	-	-	D	C	-	-	A	Q	G	-	P	T	-	-	-	-	-	-	-	-	-	-	-	-	-	A	-	P	-	-	-	-	-	-	Y	-	-	-
		AJ294909/O/CAM/2/1998	-	-	-	-	-	-	-	-	-	-	-	-	-	-	A	?	-	-	-	-	-	-	-	-	-	-	-	-	-	-	-	-	-	-	-	-	-	D	C	-	-	A	Q	G	-	P	T	-	-	-	-	-	-	-	-	-	-	-	-	-	A	-	P	-	-	-	-	-	-	Y	-	-	-
		AJ294910/O/CAM/3/1998	-	-	-	-	-	-	-	-	-	-	-	-	-	-	A	L	-	-	-	-	-	-	-	-	-	-	-	-	-	-	-	-	-	-	-	-	-	N	C	-	-	A	Q	G	-	P	T	-	-	-	-	-	-	-	-	-	-	-	-	-	A	-	P	-	-	-	-	-	-	Y	-	-	-
		SA/AJ296328/VIT/7/1997	-	-	-	-	-	-	-	-	-	-	-	-	-	-	A	-	-	-	-	-	-	-	-	-	-	-	-	-	-	-	-	-	-	-	-	-	-	N	C	-	-	A	Q	G	P	L	A	-	-	-	-	-	-	-	-	-	-	-	-	-	A	-	P	-	-	-	-	-	-	Y	-	-	-
		EU667442/O/LAO/2/1999	A	-	-	-	-	-	-	-	-	-	-	-	-	-	A	-	-	-	-	-	-	-	-	-	-	-	-	-	-	-	-	-	-	-	-	-	-	N	C	-	-	A	Q	G	P	L	A	-	-	-	-	-	-	-	-	-	-	-	-	-	A	-	P	-	-	-	-	-	-	Y	-	-	-
		EU667440/O/LAO/1/1998	A	-	-	-	-	-	-	-	-	-	-	-	-	-	A	-	-	-	-	-	-	-	-	-	-	-	-	-	-	-	-	-	-	-	-	-	-	N	C	-	-	A	Q	G	P	L	A	-	-	-	-	-	-	-	-	-	-	-	-	-	A	-	P	-	-	-	-	-	-	Y	-	-	-
ME-SA	PanAsia-2	ME-SA/PanAsia-2/ANT-10/KY091282/O/IRN/88/2009	-	-	-	-	-	-	-	-	-	-	-	-	-	-	A	-	-	-	-	-	-	-	-	-	-	-	-	-	-	-	-	-	-	-	-	-	-	N	C	-	-	-	E	S	-	-	T	-	-	-	-	-	-	-	-	-	-	-	-	-	A	-	-	-	-	-	-	-	-	Y	-	-	-
		PA2/KJ831726/PAK/1/2010	-	-	-	-	-	-	-	-	-	-	-	-	-	-	A	-	-	-	-	-	-	-	-	-	-	-	-	-	-	-	-	-	-	-	-	-	-	N	C	-	-	-	E	S	H	-	T	-	-	-	-	-	-	-	-	-	-	-	-	-	A	-	-	-	-	-	-	-	-	Y	-	-	-
		PA2/KR149715/O/IRN/31/2010	-	-	-	-	-	-	-	-	-	-	-	-	-	-	A	-	-	-	-	-	-	-	-	-	-	-	-	-	-	-	-	-	-	-	-	-	-	N	C	-	-	-	E	S	R	-	T	-	-	-	-	-	-	-	-	-	-	-	-	-	A	-	-	-	-	-	-	-	-	Y	-	-	-
		PA2/KR149717/O/UAE/2/2010	-	-	-	-	-	-	-	-	-	-	-	-	-	-	A	-	-	-	-	-	-	-	-	-	-	-	-	-	-	-	-	-	-	-	-	-	-	N	C	-	-	-	E	S	-	-	T	-	-	-	-	-	-	-	-	-	-	-	-	-	A	-	-	-	-	-	-	-	-	Y	-	-	-
		ME-SA/PanAsia-2/KY091281/O/IRN/8/2005	-	-	-	-	-	-	-	-	-	-	-	-	-	-	A	-	-	-	-	-	-	-	-	-	-	-	-	-	-	-	-	-	-	-	-	-	-	N	-	-	-	-	E	S	P	-	T	-	-	-	-	-	-	-	-	-	-	-	-	-	A	-	-	-	-	-	-	-	-	Y	-	-	-
		ME-SA/PanAsia-2/FAR-09/KY091284/O/IRN/31/2009	-	-	-	-	-	-	-	-	-	-	-	-	-	-	A	-	-	-	-	-	-	-	-	-	-	-	-	-	-	-	-	-	-	-	-	-	-	-	C	-	-	-	E	S	H	-	T	-	-	-	-	-	-	-	-	-	-	-	-	-	A	-	-	-	-	-	-	-	-	Y	-	-	-
		ME-SA/PanAsia-2/BAL-09/KY091283/O/IRN/18/2010	K	-	-	-	-	-	-	-	-	-	-	-	-	-	A	-	-	-	-	-	-	-	-	-	-	-	-	-	-	-	-	-	-	-	-	-	-	N	C	-	-	-	E	S	H	A	T	-	-	-	-	-	-	-	-	-	-	-	-	-	A	-	-	-	-	-	-	-	-	Y	-	-	-
		ME-SA/PanAsia-2/PUN-10/KY091285/O/PAK/16/2010	-	-	-	-	-	-	-	-	-	-	-	-	-	-	A	-	-	-	-	-	-	-	-	-	-	-	-	-	-	-	-	-	-	-	-	-	-	-	C	R	-	-	E	S	Q	-	-	-	-	-	-	-	-	-	-	-	-	-	R	-	-	A	-	-	-	-	-	-	-	-	Y	-	-	-
		PA2/HQ439233/O/KAB/AFG/L643/2009	-	-	-	-	-	-	-	-	-	-	-	-	-	-	A	-	-	-	-	-	-	-	-	-	-	-	-	-	-	-	-	-	-	-	-	-	-	N	C	-	-	-	E	S	-	-	T	-	-	-	-	-	-	-	-	-	-	-	-	-	A	-	V	-	-	-	-	-	-	Y	-	-	-
		PA2/HQ439234/O/BAG/AFG/L1494/2009	-	-	-	-	-	-	-	-	-	-	-	-	-	-	A	-	-	-	-	-	-	-	-	-	-	-	-	-	-	-	-	-	-	-	-	-	-	D	C	-	-	-	E	S	-	-	T	-	-	-	-	-	-	-	-	-	-	-	-	-	A	-	V	-	-	-	-	-	-	Y	-	-	-
		PA2/HQ439232/O/KUN/AFG/L630/2009	-	-	-	-	-	-	-	-	-	-	-	-	-	-	A	-	-	-	-	-	-	-	-	-	-	-	-	-	-	-	-	-	-	-	-	-	-	D	C	-	-	-	E	S	-	-	T	-	-	-	-	-	-	-	-	-	-	-	-	-	A	-	V	-	-	-	-	-	-	Y	-	-	-
	PanAsia	ME-SA/PanAsia/AJ539141/O/UKG/35/2001	-	-	-	-	-	-	-	-	-	-	-	-	-	-	A	-	-	-	-	-	-	-	-	-	-	-	-	-	-	-	-	-	-	-	-	-	-	N	C	-	-	-	E	S	P	V	T	-	-	-	-	-	-	-	-	-	-	-	-	-	A	-	-	-	-	-	-	-	-	Y	-	-	-
		MESA/AJ303499/IRQ/30/2000	-	-	-	-	-	-	-	-	-	-	-	-	-	-	A	-	-	-	-	-	-	-	-	-	-	-	-	-	-	-	-	-	-	-	-	-	-	N	C	-	-	-	E	S	P	V	T	-	-	-	-	-	-	-	-	-	-	-	-	-	A	-	-	-	-	-	-	-	-	Y	-	-	-
		PA/HQ116172/O/CAM/4/2006	-	-	-	-	-	-	-	-	-	-	-	-	-	-	A	-	-	-	-	-	-	-	-	-	-	-	-	-	-	-	-	-	-	-	-	-	-	N	C	-	-	-	E	R	P	V	T	-	-	-	-	-	-	-	-	-	-	-	-	-	A	-	-	-	-	-	-	-	-	Y	-	-	-
		PA/HQ116173/O/CAM/5/2006	-	-	-	-	-	-	-	-	-	-	-	-	-	-	A	-	-	-	-	-	-	-	-	-	-	-	-	-	-	-	-	-	-	-	-	-	-	N	C	-	-	-	E	R	P	V	A	-	-	-	-	-	-	-	-	-	-	-	-	-	A	-	-	-	-	-	-	-	-	Y	-	-	-
		PA/HQ116171/O/CAM/1/2004	A	-	-	-	-	-	-	-	-	-	-	-	-	-	A	-	-	-	-	-	-	-	-	-	-	-	-	-	-	-	-	-	-	-	-	-	-	N	C	-	-	-	E	S	P	V	T	-	-	-	-	-	-	-	-	-	-	-	-	-	A	-	-	-	-	-	-	-	-	Y	-	-	-
		PA/HQ116174/O/CAM/1/2008	A	-	-	-	-	-	-	-	-	-	-	-	-	-	A	-	-	-	-	-	-	-	-	-	-	-	-	-	-	-	-	-	-	-	-	-	-	N	C	R	-	-	E	S	P	V	T	-	-	-	-	-	-	-	-	-	-	-	-	-	A	-	-	-	-	-	-	-	-	Y	-	-	-
		ME-SA/PanAsia/EU667451/O/LAO/2/2003	-	-	-	-	-	-	-	-	-	-	-	-	-	-	A	-	-	-	-	-	-	-	-	-	-	-	-	-	-	-	-	-	-	-	-	-	-	N	C	R	-	-	E	G	P	V	T	S	-	-	-	-	-	-	-	-	-	-	-	-	A	-	-	-	-	-	-	-	-	Y	-	-	-
	Irn-2001	Irn2001/DQ165062/O/PAK/15/2002	I	-	-	-	-	-	-	-	-	-	-	-	-	-	A	-	-	-	-	-	-	-	-	-	-	-	-	-	-	-	-	-	-	-	-	-	-	N	C	-	-	-	-	S	P	V	T	-	-	-	-	-	-	-	-	-	-	-	-	-	A	-	-	-	-	-	-	-	-	Y	-	-	-
		Irn2001/DQ165063/O/PAK/16/2002	I	-	-	-	-	-	-	-	-	-	-	-	-	-	A	-	-	-	-	-	-	-	-	-	-	-	-	-	-	-	-	-	-	-	-	-	-	N	C	-	-	-	-	S	P	V	T	-	-	-	-	-	-	-	-	-	-	-	-	-	A	-	-	-	-	-	-	-	-	Y	-	-	-
		Irn2001/DQ165064/O/PAK/18/2002	I	-	-	-	-	-	-	-	-	-	-	-	-	-	A	-	-	-	-	-	-	-	-	-	-	-	-	-	-	-	-	-	-	-	-	-	-	N	C	-	-	-	-	S	P	V	T	-	-	-	-	-	-	-	-	-	-	-	-	-	A	-	-	-	-	-	-	-	-	Y	-	-	-
		AJ296325/TUR/1/1996	-	-	-	-	-	-	-	-	-	-	-	-	-	-	A	?	-	-	-	-	E	-	-	-	-	-	-	-	-	-	-	-	-	-	-	-	-	N	C	-	-	-	E	S	-	V	T	-	-	-	-	-	-	-	-	-	-	-	-	-	A	-	-	-	-	-	-	-	-	Y	-	-	-
	Ind-2001	ME-SA/Ind-2001a/DQ164904/O/KUW/3/1997	-	-	-	-	-	-	-	-	-	-	-	-	-	-	A	-	-	-	-	-	-	-	-	-	-	-	-	-	-	-	-	-	-	-	-	-	-	D	C	-	-	-	E	S	A	V	T	-	-	-	-	-	-	-	-	-	-	-	-	-	A	-	-	-	-	-	-	-	-	Y	-	-	-
		ME-SA/Ind-2001b/DQ164941/O/OMN/7/2001	K	-	-	-	-	-	-	-	-	-	-	-	-	-	A	-	-	-	-	-	-	-	-	-	-	-	-	-	-	-	-	-	-	-	-	-	-	N	C	-	-	-	E	G	A	V	T	-	-	-	-	-	-	-	-	-	-	-	-	-	A	-	-	-	-	-	-	-	-	Y	-	-	-
		ME-SA/Ind-2001c/KM921876/O/UAE/4/2008	-	-	-	-	-	-	-	-	-	-	-	-	-	-	A	-	-	-	-	-	-	-	-	-	-	-	-	-	-	-	-	-	-	-	-	-	-	N	C	R	-	-	-	V	-	V	T	-	-	-	-	-	-	-	-	-	-	-	-	-	A	-	A	-	-	-	-	-	-	Y	-	-	-
		MF947475/SRL/2/2014	-	-	-	-	-	-	-	-	-	-	-	-	-	-	A	-	-	-	-	-	-	-	-	-	-	-	-	-	-	-	-	-	-	-	-	-	-	N	C	-	-	-	E	S	N	V	P	-	-	-	-	-	-	-	-	-	-	-	-	-	A	-	P	-	-	-	-	-	-	Y	-	-	-
		MF947474/SRL/1/2014	-	-	-	-	-	-	-	-	-	-	-	-	-	-	A	-	-	-	-	-	-	-	-	-	-	-	-	-	-	-	-	-	-	-	-	-	-	N	C	-	-	-	E	S	N	V	P	-	-	-	-	-	-	-	-	-	-	-	-	-	A	-	P	-	-	-	-	-	-	Y	-	-	-
		MF947473/SRL/2/2013	A	-	-	-	-	-	-	-	-	-	-	-	-	-	A	-	-	-	-	-	-	-	-	-	-	-	-	-	-	-	-	-	-	-	-	-	-	N	C	-	-	-	E	S	N	V	P	-	-	-	-	-	-	-	-	-	-	-	-	-	A	-	P	-	-	-	-	-	-	Y	-	-	-
		ME-SA/Ind-2001d/KM921814/O/BHU/3/2009	A	-	-	-	-	-	-	-	-	-	-	-	-	-	A	-	-	-	-	-	-	-	-	-	-	-	-	-	-	-	-	-	-	-	-	-	-	N	C	-	-	-	E	S	D	V	T	-	-	-	-	-	-	E	-	-	-	-	-	-	A	-	-	-	-	-	-	-	-	Y	-	-	-
	Srl-97	MF947468/SRL/13/2010	-	-	-	-	-	-	-	-	-	-	-	-	-	-	A	-	-	-	-	I	-	-	-	-	-	-	-	-	-	-	-	-	-	-	-	-	-	-	C	R	-	-	E	S	H	E	T	-	-	-	-	-	-	-	-	-	-	-	-	-	A	-	A	-	-	-	-	-	-	Y	-	-	-
		MF947467/SRL/9/2010	-	-	-	-	-	-	-	-	-	-	-	-	-	-	A	-	-	-	-	-	-	-	-	-	-	-	-	-	-	-	-	-	-	-	-	-	-	-	C	R	-	-	E	S	H	E	T	-	-	-	-	-	-	-	-	-	-	-	-	-	A	-	A	-	-	-	-	-	-	Y	-	-	-
		KJ831703/SRL/1/2009	-	-	-	-	-	-	-	-	-	-	-	-	-	-	A	-	-	-	-	-	-	-	-	-	-	-	-	-	-	-	-	-	-	-	-	-	-	-	C	R	-	-	E	S	H	E	T	-	-	-	-	-	-	-	-	-	-	-	R	-	-	A	-	A	-	-	-	-	-	-	Y	-	-	-
		MF947463/SRL/1/2009	-	-	-	-	-	-	-	-	-	-	-	-	-	-	A	-	-	-	-	-	-	-	-	-	-	-	-	-	-	-	-	-	-	-	-	-	-	-	C	R	-	-	E	S	H	E	T	-	-	-	-	-	-	-	-	-	-	-	R	-	-	A	-	A	-	-	-	-	-	-	Y	-	-	-
		MF947455/SRL/4/1999	-	-	-	-	-	-	-	-	-	-	-	-	-	-	A	-	-	-	-	-	-	-	-	-	-	-	-	-	-	-	-	-	-	-	-	-	-	N	C	-	-	-	E	S	-	E	T	-	-	-	-	-	-	-	-	-	-	-	-	-	T	-	A	-	-	-	-	-	-	Y	-	-	-
		MF947453/SRL/1/1999	-	-	-	-	-	-	-	-	-	-	-	-	-	-	A	-	-	-	-	-	-	-	-	-	-	-	-	-	-	-	-	-	-	-	-	-	-	N	C	-	-	-	E	S	-	E	T	-	-	-	-	-	-	-	-	-	-	-	-	-	T	-	A	-	-	-	-	-	-	Y	-	-	-
		MF947454/SRL/3/1999	-	-	-	-	-	-	-	-	-	-	-	-	-	-	A	-	-	-	-	-	-	-	-	-	-	-	-	-	-	-	-	-	-	-	-	-	-	N	C	-	-	-	E	S	-	E	T	-	-	-	-	-	-	-	-	-	-	-	-	-	A	-	A	-	-	-	-	-	-	Y	-	-	-
		AJ303531/SRL/2/1997	-	-	-	-	-	-	-	-	-	-	-	-	-	-	A	-	-	-	-	-	-	-	-	-	-	-	-	-	-	-	-	-	-	-	-	-	-	N	C	-	-	-	E	S	-	E	T	-	-	-	-	-	-	-	-	-	-	-	-	-	A	-	A	-	-	-	-	-	-	Y	-	-	-
		ME-SA/Srl-97/AJ303531/O/SRL/2/1997	-	-	-	-	-	-	-	-	-	-	-	-	-	-	A	-	-	-	-	-	-	-	-	-	-	-	-	-	-	-	-	-	-	-	-	-	-	N	C	-	-	-	E	S	-	E	T	-	-	-	-	-	-	-	-	-	-	-	-	-	A	-	A	-	-	-	-	-	-	Y	-	-	-
		MF947470/SRL/4/2011	-	-	-	-	-	-	-	-	-	-	-	-	Y	-	A	-	-	-	-	-	-	-	-	-	-	-	-	-	-	-	-	-	-	-	-	-	-	N	C	-	-	-	E	S	P	E	T	-	-	-	-	-	-	-	-	-	-	-	R	-	-	A	-	A	-	-	-	-	-	-	Y	-	-	-
	Pak-98	Pak98/DQ165068/O/PAK/16/2003	A	-	-	E	-	-	-	-	-	-	-	-	-	-	A	-	-	-	-	-	-	-	-	-	-	-	-	-	-	-	-	-	-	-	-	-	-	N	C	-	-	-	G	G	P	V	T	-	-	-	-	-	-	-	-	-	-	D	-	-	-	A	-	A	-	-	-	-	-	-	Y	-	-	-
		Pak98/DQ165070/O/PAK/73/2003	A	-	-	E	-	-	-	-	-	-	-	-	-	-	A	-	-	-	-	-	-	-	-	-	-	-	-	-	-	-	-	-	-	-	-	-	-	N	C	-	-	-	G	G	P	V	T	-	-	-	-	-	-	-	-	-	-	D	-	-	-	A	-	A	-	-	-	-	-	-	Y	-	-	-
		Pak98/DQ165069/O/PAK/17/2003	A	-	-	E	-	-	-	-	-	-	-	-	-	-	A	-	-	-	-	-	-	-	-	-	-	-	-	-	-	-	-	-	-	-	-	-	-	N	C	-	-	-	E	G	P	V	T	-	-	-	-	-	-	-	-	-	-	-	-	-	-	A	-	A	-	-	-	-	-	-	Y	-	-	-
	Unnamed	ME-SA/unnamed/AF204276/O/IND/R2/1975	N	-	-	-	-	-	-	-	-	-	-	-	-	-	A	-	-	-	-	-	-	-	-	-	-	-	-	Q	-	-	-	-	-	-	-	-	-	N	C	-	-	-	-	G	-	V	T	-	I	-	-	-	-	-	-	-	-	-	-	-	A	-	-	-	-	-	-	-	-	Y	-	-	-
		ME-SA/unnamed/AF292107/O/IND/53/1979	-	-	-	-	-	-	-	-	-	-	-	-	-	-	A	-	-	-	-	-	-	-	-	-	-	-	-	-	-	-	-	-	-	-	-	-	-	N	C	R	-	-	E	S	T	V	T	-	-	-	-	-	-	-	-	-	-	-	-	-	A	-	-	-	-	-	-	-	-	Y	-	-	-
	Tur-69	EU553844/O/ISR/1/1992	-	-	-	N	-	-	-	-	-	-	-	-	-	-	A	-	-	-	-	-	-	-	-	-	-	-	-	-	-	-	-	-	-	-	-	-	-	N	C	-	-	-	-	G	A	V	A	-	-	-	-	-	-	-	-	-	-	-	-	N	-	A	-	A	-	-	-	-	-	-	Y	-	-	-
		EU553845/O/ITL/1/1993	-	-	-	N	-	-	-	-	-	-	-	-	-	-	A	-	-	-	-	-	-	-	-	-	-	-	-	-	-	-	-	-	-	-	-	-	-	N	C	-	-	R	-	R	A	V	A	-	A	-	-	-	-	-	-	-	-	-	-	-	A	-	A	-	-	-	-	-	-	Y	-	-	-
		EU553846/O/OMN/3/1991	-	-	-	N	-	-	-	-	-	-	-	-	-	-	A	-	-	-	-	-	-	-	-	-	-	-	-	-	-	-	-	-	-	-	-	-	-	N	C	-	-	-	-	G	A	V	T	-	-	-	-	-	-	-	-	-	-	-	-	-	A	-	A	-	-	-	-	-	-	Y	-	-	-
		O/SRL/1/1991	-	-	-	N	-	-	-	-	-	-	-	-	-	-	A	-	-	-	-	-	-	-	-	-	-	-	-	-	-	-	-	-	-	-	-	-	-	N	C	-	-	-	-	G	A	V	T	-	-	-	-	-	-	-	-	-	-	-	-	-	A	-	A	-	-	-	-	-	-	Y	-	-	-

Topotype	Liniage	Isolate	97	98	99	100	101	102	103	104	105	106	107	108	109	110	111	112	113	114	115	116	117	118	119	120	121	122	123	124	125	126	127	128	129	130	131	132	133	134	135	136	137	138	139	140	141	142	143	144	145	146	147	148	149	150	151	152	153	154	155	156	157	158	159	160	161	162	163	164	165	166	167	168	169
Reference Sequnce		NC 004004 ref seq	T	A	L	D	N	T	T	N	P	T	A	Y	H	K	E	P	L	T	R	L	A	L	P	Y	T	A	P	H	R	V	L	A	T	V	Y	N	G	S	S	K	Y	G	D	T	S	T	N	N	V	R	G	D	L	Q	V	L	A	Q	K	A	E	R	T	L	P	T	S	F	N	F	G	A	I
		ME-SA/unnamed/AY593823/O1/Manisa/TUR/1969	A	-	-	-	-	-	-	-	-	-	-	-	-	-	A	-	-	-	-	-	-	-	-	-	-	-	-	-	-	-	-	-	-	-	-	-	-	N	-	-	-	-	-	G	T	V	A	-	-	-	-	-	-	-	-	-	-	-	-	-	A	-	A	-	-	-	-	-	-	Y	-	-	-
WCSA-1	Srl-77	O/SRL/7/88/1987	A	-	-	N	-	-	-	-	-	-	-	-	-	-	A	-	-	-	-	-	-	-	-	-	-	-	-	-	-	-	-	-	-	-	-	-	-	N	C	-	-	-	-	G	P	V	T	-	-	-	-	-	-	-	-	-	-	-	-	-	A	-	P	-	-	-	-	-	-	Y	-	-	-
		O/SRL/5/1987	A	-	-	N	-	-	-	-	-	-	-	-	-	-	A	-	-	-	-	-	-	-	-	-	-	-	-	-	-	-	-	-	-	-	-	-	-	N	C	-	-	-	-	G	P	V	T	-	-	-	-	-	-	-	-	-	-	-	-	-	A	-	P	-	-	-	-	-	-	Y	-	-	-
		O/SRL/4/88/1987	A	-	-	N	-	-	-	-	-	-	-	-	-	-	A	-	-	-	-	-	-	-	-	-	-	-	-	-	-	-	-	-	-	-	-	-	-	N	C	-	-	-	-	G	P	V	T	-	-	-	-	-	-	-	-	-	-	-	-	-	A	-	P	-	-	-	-	-	-	Y	-	-	-
		O/SRL/3/88/1987	A	-	-	N	-	-	-	-	-	-	-	-	-	-	A	-	-	-	-	-	-	-	-	-	-	-	-	-	-	-	-	-	-	-	-	-	-	N	C	-	-	-	-	G	P	V	T	-	-	-	-	-	-	-	-	-	-	-	-	-	A	-	P	-	-	-	-	-	-	Y	-	-	-
		O/SRL/6/88/1987	A	-	-	N	-	-	-	-	-	-	-	-	-	-	A	-	-	-	-	-	-	-	-	-	-	-	-	-	-	-	-	-	-	-	-	-	-	N	C	-	-	-	-	G	P	V	T	-	-	-	-	-	-	-	-	-	-	-	-	-	A	-	P	-	-	-	-	-	-	Y	-	-	-
		O/SRL/9/88/1987	A	-	-	N	-	-	-	-	-	-	-	-	-	-	A	-	-	-	-	-	-	-	-	-	-	-	-	-	-	-	-	-	-	-	-	-	-	N	C	-	-	-	-	G	P	V	T	-	-	-	-	-	-	-	-	-	V	-	-	-	A	-	P	-	-	-	-	-	-	Y	-	-	-
		O/SRL/2/88/1987	A	-	-	N	-	-	-	-	-	-	-	-	-	-	A	-	-	-	-	-	-	-	-	-	-	-	-	-	-	-	-	-	-	-	-	-	-	N	C	-	-	-	-	G	P	V	T	-	-	-	-	-	-	-	-	-	-	-	-	-	A	-	P	-	-	-	-	-	-	Y	-	-	-
		O/SRL/1/88/1987	A	-	-	N	-	-	-	-	-	-	-	-	-	-	A	-	-	-	-	-	-	-	-	-	-	-	-	-	-	-	-	-	-	-	-	-	-	N	C	-	-	-	-	G	P	V	T	-	-	-	-	-	-	-	-	-	-	-	-	-	A	-	P	-	-	-	-	-	-	Y	-	-	-
		O/SRL/8/88/1987	A	-	-	N	-	-	-	-	-	-	-	-	-	-	A	-	-	-	-	-	-	-	-	-	-	-	-	-	-	-	-	-	-	-	-	-	-	N	C	-	-	-	-	G	P	V	T	-	-	-	-	-	-	-	-	-	-	-	-	-	A	-	P	-	-	-	-	-	-	Y	-	-	-
		O/SRL/10/1988	A	-	-	N	-	-	-	-	-	-	-	-	-	-	A	-	-	-	-	-	-	-	-	-	-	-	-	-	-	-	-	-	-	-	-	-	-	N	C	-	-	-	-	G	P	V	T	-	-	-	-	-	-	-	-	-	-	-	-	-	A	-	P	-	-	-	-	-	-	Y	-	-	-
		O/SRL/1/1997	A	-	-	N	-	-	-	-	-	-	-	-	-	-	A	-	-	-	-	-	-	-	-	-	-	-	-	-	-	-	-	-	-	-	-	-	-	N	C	R	-	-	G	G	P	V	T	-	-	-	-	-	-	-	-	-	-	-	-	-	A	-	S	-	-	-	-	-	-	Y	-	-	-
		O/SRL/1/78/1977	A	-	-	-	-	-	-	-	-	-	-	-	-	-	A	-	-	-	-	-	-	-	-	-	-	-	-	-	-	-	-	-	-	A	-	-	-	N	C	-	-	-	A	G	P	V	T	-	-	-	-	-	-	-	-	-	-	-	-	-	A	-	P	-	-	-	-	-	-	Y	-	-	-
		O/SRL/3/1978	A	-	-	-	-	-	-	-	-	-	-	-	-	-	A	-	-	-	-	-	-	-	-	-	-	-	-	-	-	-	-	-	-	A	-	-	-	N	C	-	-	-	A	G	P	V	T	-	-	-	-	-	-	-	-	-	-	-	-	-	A	-	P	-	-	-	-	-	-	Y	-	-	-
		O/SRL/2/1978	A	-	-	-	-	-	-	-	-	-	-	-	-	-	A	-	-	-	-	-	-	-	-	-	-	-	-	-	-	-	-	-	-	A	-	-	-	N	C	-	-	-	A	G	P	V	T	-	-	-	-	-	-	-	-	-	-	-	-	-	A	-	P	-	-	-	-	-	-	Y	-	-	-
		O/SRL/87/VS/1987	A	-	-	-	-	-	-	-	-	-	-	-	-	-	A	-	-	-	-	-	-	-	-	-	-	-	-	-	-	-	-	-	-	A	-	-	-	N	C	-	-	-	A	G	P	V	T	-	-	-	-	-	-	-	-	-	-	-	-	-	A	-	P	P	-	-	-	-	-	Y	-	-	-
		O/SRL/3/1987	A	-	-	-	-	-	-	-	-	-	-	-	-	-	A	-	-	-	-	-	-	-	-	-	-	-	-	-	-	-	-	-	-	A	-	-	-	N	C	-	-	-	A	G	P	V	T	-	-	-	-	-	-	-	-	-	D	-	-	-	A	-	P	P	-	-	-	-	-	Y	-	-	-
	Syr-62	AJ004679/O/1492/Russia/USSR/88/1988	-	-	-	-	-	-	-	-	-	-	-	-	-	-	A	-	-	-	-	-	-	-	-	-	-	-	-	-	-	-	-	-	-	-	-	-	-	N	C	-	-	-	T	G	P	V	T	-	A	-	-	-	-	-	-	-	G	-	-	-	A	-	P	-	-	-	-	-	-	Y	-	-	-
		O/SYR/18/1962	-	-	-	-	-	-	-	-	-	-	-	-	-	-	A	-	-	-	-	-	-	-	-	-	-	-	-	-	-	-	-	-	-	-	-	-	-	N	C	-	-	-	T	G	P	V	T	-	-	-	-	-	-	-	-	-	-	-	-	-	A	-	P	-	-	-	-	-	-	Y	-	-	-
		AY593834/O/Rey/IRN/66/1966	-	-	-	-	-	-	-	-	-	-	-	-	-	-	A	-	-	-	-	-	-	-	-	-	-	-	-	-	-	-	-	-	-	-	-	-	-	N	C	-	-	-	T	G	P	V	A	-	-	-	-	-	-	-	-	-	-	-	-	-	A	-	P	-	-	-	-	-	-	Y	-	-	-
		AJ004663/O/738/Tajikistan/USSR/74/1974	-	-	-	-	-	A	-	-	-	-	-	-	-	-	A	-	-	-	-	-	-	-	-	-	-	-	-	-	-	-	-	-	-	-	-	-	-	N	C	-	-	-	T	G	P	V	T	-	-	-	-	-	-	-	-	-	-	-	-	-	A	-	P	-	-	-	-	-	-	Y	-	-	-
EA-3		EA-3/unnamed/FJ798109/O/ETH/3/2004	S	-	-	-	-	-	-	-	-	-	-	-	Y	-	A	-	-	-	-	-	-	-	-	-	-	-	-	-	-	-	-	-	-	-	-	-	-	N	C	-	-	-	V	-	-	V	T	-	-	-	-	-	-	-	-	-	-	-	-	-	A	-	P	-	-	-	-	-	-	Y	-	-	-
		EA-3/unnamed/FJ798137/O/ETH/1/2007	S	-	-	-	-	-	-	-	-	-	-	-	-	-	A	-	-	-	-	-	-	-	-	-	-	-	-	-	-	-	-	-	-	-	-	-	-	N	C	-	-	-	E	-	P	V	T	-	-	-	-	-	-	-	-	-	-	-	-	V	A	-	-	-	-	-	-	-	-	Y	-	-	-
		EA-3/unnamed/DQ165075/O/SUD/2/1986	-	-	-	-	-	-	-	-	-	-	-	-	-	-	A	-	-	-	-	-	-	-	-	-	-	-	-	-	-	-	-	-	-	-	-	-	-	N	C	R	-	-	E	A	P	V	T	-	-	-	-	-	-	-	-	-	-	-	-	-	A	-	-	-	-	-	-	-	-	Y	-	-	-
		EA-3/unnamed/FJ798127/O/ETH/2/2006	-	-	-	-	-	S	-	-	-	-	-	-	-	-	A	-	-	-	-	-	-	-	-	-	-	-	-	-	-	-	-	-	-	-	-	-	-	E	C	-	-	R	G	-	P	L	S	-	-	-	-	-	-	-	-	-	-	-	-	-	A	-	A	-	-	-	-	-	-	Y	-	-	-
EA-1		EA/AJ303511/KEN/83/1979	S	-	-	-	-	-	-	-	-	-	-	-	-	-	A	-	-	-	-	-	-	-	-	-	-	-	-	-	-	-	-	-	-	-	-	-	-	N	C	R	-	-	-	A	P	V	T	-	-	-	-	-	-	-	-	-	-	-	-	-	A	-	-	-	-	-	-	-	-	Y	-	-	-
		EA/AJ303514/KEN/2/95/K10/1995	S	-	?	-	-	-	-	T	?	-	-	-	-	-	A	-	-	-	-	-	-	-	-	-	-	-	-	-	-	-	-	-	-	-	-	-	-	N	C	R	-	?	R	A	P	V	T	-	-	-	-	-	-	-	-	-	-	-	-	-	A	-	-	-	-	-	-	-	-	Y	-	-	-
		EA/AJ296327/UGA/5/1996	S	-	-	-	-	-	-	-	-	-	-	-	-	-	A	-	-	-	-	-	-	-	-	-	-	-	-	-	-	-	-	-	-	-	-	-	-	N	C	R	-	-	V	-	P	V	T	-	-	-	-	-	-	-	-	-	V	-	-	-	-	A	-	-	-	-	-	-	-	-	Y	-	-	-
EA-2		EA-2/unnamed/DQ165073/O/KEN/5/2002	S	-	-	-	-	-	-	-	-	-	-	-	-	-	A	-	-	-	-	-	-	-	-	-	-	-	-	-	-	-	-	-	-	-	-	-	-	N	C	-	-	-	G	-	P	V	T	-	-	-	-	-	-	-	-	-	-	-	-	-	A	-	-	-	-	-	-	-	-	Y	-	-	-
		EA-2/unnamed/DQ165077/O/UGA/3/2002	A	-	-	-	-	-	-	-	-	-	-	-	-	-	A	-	-	-	-	-	-	-	-	-	-	-	-	-	-	-	-	-	-	-	-	-	-	N	C	-	-	S	-	S	-	V	T	-	-	-	-	-	-	-	-	-	-	-	-	-	A	-	A	-	-	-	-	-	-	Y	-	-	-
		EA-2/unnamed/KF561679/O/TAN/2/2004	-	-	-	-	-	-	-	-	-	-	-	-	-	-	A	-	-	-	-	-	-	-	-	-	-	-	-	-	-	-	-	-	-	-	-	-	-	N	C	-	-	-	G	-	P	V	T	-	-	-	-	-	-	-	-	-	-	-	-	-	A	-	-	-	-	-	-	-	-	Y	-	-	-
		EA-2/unnamed/DQ165074/O/MAL/1/1998	-	-	-	-	-	-	-	-	-	-	-	-	Y	-	A	-	-	-	-	-	-	-	-	-	-	-	-	-	-	-	-	-	-	-	-	-	-	N	C	-	-	D	-	-	P	V	T	-	-	-	-	-	-	-	-	-	-	-	-	-	A	-	-	-	-	-	-	-	-	Y	-	-	-
EA-4		EA-4/unnamed/FJ798141/O/ETH/58/2005	S	-	-	-	-	-	-	-	-	-	-	-	-	-	-	-	-	-	-	-	-	-	-	-	-	-	-	-	-	-	-	-	-	-	-	-	-	N	C	-	-	-	T	A	P	V	A	-	-	-	-	-	-	-	-	-	-	-	-	-	A	-	-	-	-	-	-	-	-	Y	-	-	-
		EA-4/unnamed/HM211075/O/UGA/17/1998	S	-	-	-	-	-	-	-	-	-	-	-	-	-	-	-	-	-	-	-	-	-	-	-	-	-	-	-	-	-	-	-	-	-	-	-	-	N	C	-	-	D	T	A	P	L	T	-	-	-	-	-	-	-	-	-	-	-	-	-	A	-	-	-	-	-	-	-	-	Y	-	-	-
WA		WA/AJ303481/ALG/1/1999	-	-	-	-	-	-	-	-	-	-	-	-	-	-	A	?	-	-	-	-	?	-	-	-	-	-	-	-	-	-	-	-	-	-	-	-	-	-	C	R	-	S	G	A	V	-	P	-	-	-	-	-	-	-	-	-	-	R	R	-	A	P	M	-	-	-	-	-	-	-	-	-	-
		WA/AJ303485/CIV/8/1999	S	-	-	-	-	-	-	-	-	-	-	-	-	-	A	-	-	-	-	-	-	-	-	-	-	-	-	-	-	-	-	-	-	-	-	-	-	-	C	R	-	S	G	A	V	-	P	-	-	-	-	-	-	-	-	-	-	R	R	-	A	P	M	-	-	-	-	-	-	-	-	-	-
		WA/AJ303488/GHA/5/1993	S	-	-	-	-	-	-	-	-	-	-	-	-	-	A	-	-	-	-	-	-	-	-	-	-	-	-	-	-	-	-	-	-	-	-	-	-	-	C	-	-	S	R	V	E	V	P	K	-	-	-	-	-	-	-	-	-	R	R	-	A	-	-	-	-	-	-	-	-	-	-	-	-
Cathay		CATHAY/unnamed/AF308157/O/Yunlin/TAW/1997	-	-	-	-	-	-	-	-	-	-	-	-	-	-	-	-	-	-	-	-	-	-	-	-	-	-	-	-	-	-	-	-	-	-	-	-	-	-	-	-	-	-	-	-	-	-	-	-	-	-	-	-	-	-	-	-	-	-	-	-	-	-	-	-	-	-	-	-	-	-	-	-	-
		Cathey/AJ296321/TAW/81/1997	-	-	-	-	-	-	-	-	-	-	-	-	-	-	-	-	-	-	-	-	-	-	-	-	-	-	-	-	-	-	-	-	-	-	-	-	-	-	-	-	-	-	-	-	-	-	-	-	-	-	-	-	-	-	-	-	-	-	-	-	-	-	-	-	-	-	-	-	-	-	-	-	-
		1997/AJ296322/TAW/83/1997	-	-	-	-	-	-	-	-	-	-	-	-	-	-	-	-	-	-	-	-	-	-	-	-	-	-	-	-	-	-	-	-	-	-	-	-	-	-	-	-	-	-	-	-	-	-	-	-	-	-	-	-	-	-	-	-	-	-	-	-	-	-	-	-	-	-	-	-	-	-	-	-	-
		AJ294923/O/HKN/16/1996	-	-	-	-	-	-	-	-	-	-	-	-	-	-	-	?	-	-	-	-	-	-	-	-	-	-	-	-	-	-	-	-	-	-	-	-	-	-	-	-	-	-	-	-	-	-	-	-	-	-	-	-	-	-	-	-	-	-	-	-	-	-	-	-	-	-	-	-	-	-	-	-	-
		Cathey/AJ004680/O/1685/Moscow/Russia/1995	-	-	-	-	-	-	-	-	-	-	-	-	-	-	-	-	-	-	-	-	-	-	-	-	-	-	-	-	-	-	-	-	-	-	-	-	-	-	-	-	-	-	-	-	-	-	-	-	-	-	-	-	-	-	-	-	-	-	-	-	-	-	-	-	-	-	-	-	-	-	-	-	-
		Cathey/AJ294926/O/PHI/7/1996	-	-	-	-	-	-	-	-	-	-	-	-	-	-	-	-	-	-	-	-	-	-	-	-	-	-	-	-	-	-	-	-	-	-	-	-	-	-	-	-	-	-	-	-	-	A	-	-	-	-	-	-	-	-	-	-	-	-	-	-	-	-	-	-	-	-	-	-	-	-	-	-	-
		CATHAY/unnamed/AJ294919/O/HKN/6/1983	-	-	-	-	-	-	-	-	-	-	-	-	-	-	-	?	-	-	-	-	-	-	-	-	-	-	-	-	-	-	-	-	-	-	-	-	-	-	C	-	-	S	-	A	R	V	S	-	-	-	-	-	-	-	-	-	-	-	-	-	-	-	-	-	-	-	-	-	-	-	-	-	-
		Cathey/AJ131468/GD/China/1986	-	-	-	-	-	-	-	-	-	-	-	-	-	-	A	-	-	-	-	-	-	-	-	-	-	-	-	-	-	-	-	-	-	-	-	-	-	-	C	-	-	S	-	A	R	V	S	-	-	-	-	-	-	R	-	-	-	-	-	-	-	-	A	-	-	-	-	S	-	Y	-	-	-
		Cathey/AJ294917/O/HKN/14/1982	-	-	-	-	-	-	-	-	-	-	-	-	-	-	A	L	-	-	-	-	-	-	-	-	-	-	-	-	-	-	-	-	-	-	-	-	-	-	C	-	-	S	-	A	R	A	S	-	-	-	-	-	-	-	-	-	-	-	-	-	-	-	A	-	-	-	-	-	-	Y	-	-	-
		CATHAY/unnamed/AJ294911/O/HKN/21/1970	-	-	-	-	-	-	-	-	-	-	-	-	-	-	A	-	-	-	-	-	-	-	-	-	-	-	-	-	-	-	-	-	-	-	-	-	-	-	C	-	-	S	-	-	H	M	S	-	-	-	-	-	-	-	-	-	T	-	-	-	S	-	A	-	-	-	-	-	-	Y	-	-	-
EURO-SA		EURO-SA/O1/AY593815/O1/BFS 1860/UK/1867	K	-	-	-	-	-	-	-	-	-	-	-	-	-	A	-	-	-	-	-	-	-	-	-	-	-	-	-	-	-	-	-	-	-	-	-	-	E	C	R	-	S	R	N	A	V	P	-	L	-	-	-	-	-	-	-	-	-	-	V	A	-	-	-	-	-	-	-	-	Y	-	-	-
		EuroSA/K01201/O1Campos/Brazil/1971	K	-	-	-	-	-	-	-	-	-	-	-	-	-	A	-	-	-	-	-	-	-	-	-	-	-	-	-	-	-	-	-	-	-	-	-	-	E	C	R	-	S	R	N	A	V	P	-	-	-	-	-	-	-	-	-	-	-	-	V	A	-	-	-	-	-	-	-	-	Y	-	-	-
		EuroSA/X00871/O1/Kaufbeuren/FRG/1966	K	-	-	-	-	-	-	-	-	-	-	-	-	-	A	-	-	-	-	-	-	-	-	-	-	-	-	-	-	-	-	-	-	-	-	-	-	E	C	R	-	N	R	N	A	V	P	-	L	-	-	-	-	-	-	-	-	-	-	V	A	-	-	-	-	-	-	-	-	Y	-	-	-
		EuroSA/Z21862/O/Yrigoyen/ARG/1982	K	-	-	G	-	-	-	-	-	-	-	-	Y	-	K	-	-	-	-	-	-	-	-	-	-	-	-	Y	-	-	-	-	-	-	-	-	-	E	C	T	-	S	S	N	A	V	P	-	-	-	-	-	P	N	L	-	E	-	-	-	A	-	M	-	-	A	Y	-	-	Y	-	-	-
		EuroSA/M89900/O1/Caseros/ARG/1967	Q	-	-	-	-	-	-	-	-	N	-	-	Y	-	K	R	-	-	-	-	-	-	-	-	-	-	-	-	-	-	-	-	-	-	-	-	-	E	C	-	-	S	S	?	A	V	P	-	-	-	-	-	-	N	-	-	E	-	-	-	A	-	-	-	-	-	-	-	-	Y	-	-	-
		Eurosa/M58601/O1/Lombardy/ITL/1946	S	-	-	E	-	-	-	-	-	-	-	-	-	-	A	?	-	-	-	-	-	-	-	-	-	-	-	-	-	-	-	-	-	-	-	-	-	G	-	R	-	S	R	N	T	V	P	-	-	-	-	-	-	-	-	-	T	-	-	-	A	-	-	-	-	-	-	-	-	Y	-	-	-
		EuroSA/M55287/O2/Brescia/ITL/1947	?	-	-	E	-	-	-	-	-	-	-	-	-	-	A	-	-	-	-	-	-	-	-	-	-	-	-	-	-	-	-	-	-	-	-	-	-	E	-	R	-	S	R	N	A	V	?	-	-	-	-	-	-	-	A	-	-	-	-	-	A	-	-	-	-	-	-	-	-	Y	-	-	-
		EuroSA/AJ004645/O3/Venezuela/1951	Q	-	-	-	-	-	-	-	-	-	-	-	-	-	A	-	-	-	-	-	-	-	-	-	-	-	-	-	-	-	-	-	-	-	-	-	-	R	C	R	-	S	R	D	V	V	T	-	-	I	-	-	-	-	A	-	-	H	-	-	A	-	S	-	-	-	-	-	-	Y	-	-	-
		EURO-SA/unnamed/DQ834727/O/Corrientes/ARG/2006	V	-	-	-	-	-	-	-	-	-	-	-	-	-	A	-	I	-	-	-	-	-	-	-	-	-	-	-	-	-	-	-	-	-	-	-	-	G	C	-	-	T	N	G	A	A	P	-	-	-	-	-	-	-	-	-	-	-	-	-	A	-	P	-	-	-	-	-	-	Y	-	-	-
CEY-1		O/CEY/1/71/1962	A	-	-	N	-	-	-	-	-	-	-	-	-	-	A	-	-	-	-	-	-	-	-	-	-	-	-	-	-	-	-	-	-	-	-	-	-	-	C	R	-	-	E	H	T	V	S	-	-	-	-	-	-	-	-	-	-	-	-	-	A	-	P	-	-	-	-	-	-	-	-	-	-
		O/SRL/1/76/1962	A	-	-	N	-	-	-	-	-	-	-	-	-	-	A	-	-	-	-	-	-	-	-	-	-	-	-	-	-	-	-	-	-	-	-	-	-	-	C	R	-	-	E	H	T	V	S	-	-	-	-	-	-	-	-	-	-	-	-	-	A	-	P	-	-	-	-	-	-	-	-	-	-
		O/SRL/1/1982	A	-	-	-	-	-	-	-	-	-	-	-	-	-	A	-	-	-	-	-	-	-	-	-	-	-	-	-	-	-	-	-	-	-	-	-	-	-	C	R	-	-	-	H	N	V	S	-	-	-	-	-	-	-	-	-	-	-	-	-	A	-	P	-	-	-	-	-	-	Y	-	-	-
ISA-2		ISA2/AJ303501/ISA/1/1974	A	-	-	-	-	-	-	-	-	-	-	-	-	-	K	-	-	-	-	-	-	-	-	-	-	-	-	-	-	-	-	-	-	-	-	-	-	N	C	-	-	T	S	V	A	M	A	-	E	-	-	-	-	-	-	-	T	-	-	-	A	-	P	-	-	-	-	-	-	Y	-	-	-
		ISA2/AJ303509/JAV/5/1972	A	-	-	-	-	-	-	-	-	-	-	-	-	-	-	-	-	-	-	-	-	-	-	-	-	-	-	-	-	-	-	-	-	-	-	-	-	N	C	-	-	T	S	V	A	M	A	-	G	-	-	-	-	-	-	-	T	-	-	-	A	-	P	-	-	-	-	-	-	Y	-	-	-
ISA-1		ISA1/AJ303503/ISA/8/1983	A	-	-	N	-	S	-	-	-	-	-	-	-	-	A	-	-	-	-	-	-	-	-	-	-	-	-	-	-	-	-	-	-	A	-	-	-	E	C	R	-	-	T	H	T	-	T	-	-	-	-	-	-	-	-	-	-	-	-	-	A	-	-	-	-	-	-	-	-	Y	-	-	-
		ISA1/AJ303502/ISA/9/1974	-	-	-	-	-	A	-	-	-	-	-	-	-	-	A	-	-	-	-	-	-	-	-	-	-	-	-	-	-	-	-	-	-	-	-	-	-	N	C	R	-	-	A	H	T	V	T	-	-	-	-	-	-	-	-	-	-	-	-	-	A	-	-	-	-	-	-	-	-	Y	-	-	-
		ISA1/AJ303500/ISA/1/1962	A	-	-	N	-	-	-	-	-	-	-	-	-	-	A	-	-	-	-	-	-	-	-	-	-	-	-	-	-	-	-	-	-	A	-	-	-	A	C	-	-	-	T	D	T	A	P	-	-	-	-	-	-	-	-	-	-	-	-	-	A	-	P	-	-	-	-	-	-	Y	-	-	-

Topotype	Liniage	Isolate	170	171	172	173	174	175	176	177	178	179	180	181	182	183	184	185	186	187	188	189	190	191	192	193	194	195	196	197	198	199	200	201	202	203	204	205	206	207	208	209	210	211	212	213	214
Reference Sequnce		NC 004004 ref seq	K	A	T	R	V	T	E	L	L	Y	R	M	K	R	A	E	T	Y	C	P	R	P	L	L	A	I	Q	P	S	D	A	R	H	K	Q	R	I	V	A	P	A	K	Q	-	-
SEA	May-78	O/MAY/1/1980	-	-	-	-	-	-	-	-	-	-	-	-	-	-	-	-	-	-	-	-	-	-	-	-	-	V	H	-	-	Q	-	-	-	-	-	K	-	-	-	-	V	-	-	T	L
		O/TAI/2/1980	-	-	-	-	-	-	-	-	-	-	-	-	-	-	-	-	-	-	-	-	-	-	-	-	-	V	H	-	-	Q	-	-	-	-	-	K	-	-	-	-	V	-	-	T	L
		O/MAY/9/1980	-	-	-	-	-	-	-	-	-	-	-	-	-	-	-	-	-	-	-	-	-	-	-	-	-	V	H	-	-	Q	-	-	-	-	-	K	-	-	-	-	V	-	-	T	L
		O/TAI/3/1980	-	-	-	-	-	-	-	-	-	-	-	-	-	-	-	-	-	-	-	-	-	-	-	-	-	V	H	-	-	Q	-	-	-	-	-	K	-	-	-	-	V	-	-	T	L
		O/MAY/11/1980	-	-	-	-	-	-	-	-	-	-	-	-	-	-	-	-	-	-	-	-	-	-	-	-	-	V	H	-	-	Q	-	-	-	-	-	K	-	-	-	-	V	-	-	T	L
		O/TAI/1/1980	-	-	-	-	-	-	-	-	-	-	-	-	-	-	-	-	-	-	-	-	-	-	-	-	-	V	H	-	-	Q	-	-	-	-	-	K	-	-	-	-	V	-	-	T	L
		O/MAY/7/1981	-	-	-	-	-	-	-	-	-	-	-	-	-	-	-	-	-	-	-	-	-	-	-	-	-	V	H	-	-	Q	-	-	-	-	-	K	-	-	-	-	V	-	-	T	L
		O/MAY/16/1981	-	-	-	-	-	-	-	-	-	-	-	-	-	-	-	-	-	-	-	-	-	-	-	-	-	V	H	-	-	Q	-	-	-	-	-	K	-	-	-	-	V	-	-	T	L
		O/LAO/2/1981	-	-	-	-	-	-	-	-	-	-	-	-	-	-	-	-	-	-	-	-	-	-	-	-	-	V	H	-	-	Q	-	-	-	-	-	K	-	-	-	-	V	-	-	T	L
		O/HKN/17/82/AJ294918/1982	-	-	-	-	-	-	-	-	-	-	-	-	-	-	-	-	-	-	-	-	-	-	-	-	-	V	H	-	N	Q	-	-	-	-	-	K	-	-	-	-	V	-	-	T	L
		O/MAY/19/1978	-	-	-	Q	-	-	-	-	-	-	-	-	-	-	-	-	-	-	-	-	-	-	-	-	-	V	H	-	-	Q	-	-	-	-	-	K	-	-	-	-	V	-	-	S	L
		O/MAY/24/1978	-	-	-	Q	-	-	-	-	-	-	-	-	-	-	-	-	-	-	-	-	-	-	-	-	-	V	H	-	-	Q	-	-	-	-	-	K	-	-	-	-	V	-	-	S	L
		O/MAY/1/1978	-	-	-	Q	-	-	-	-	-	-	-	-	-	-	-	-	-	-	-	-	-	-	-	-	-	V	H	-	-	Q	-	-	-	-	-	K	-	-	-	-	V	-	-	S	L
		O/MAY/3/1979	-	-	-	Q	-	-	-	-	-	-	-	-	-	-	-	-	-	-	-	-	-	-	-	-	-	-	H	-	-	Q	-	-	-	-	-	K	-	-	-	-	V	-	-	S	L
		O/MAY/1/1984	-	-	-	-	-	-	-	-	-	-	-	-	-	-	-	-	-	-	-	-	-	-	-	-	-	V	H	-	-	Q	-	-	-	-	-	K	-	-	-	-	V	-	-	T	L
		O/SRL/3/1984	-	-	-	Q	-	-	-	-	-	-	-	-	-	-	-	-	-	-	-	-	-	-	-	-	-	V	H	-	-	E	-	-	-	-	-	K	-	-	-	-	V	-	-	T	L
		O/SRL/2/1984	-	-	-	Q	-	-	-	-	-	-	-	-	-	-	-	-	-	-	-	-	-	-	-	-	-	V	H	-	-	E	-	-	-	-	-	K	-	-	-	-	V	-	-	T	L
		O/MAY/3/1981	-	-	-	-	-	-	-	-	-	-	-	-	-	-	-	-	-	-	-	-	-	-	-	-	-	V	H	-	-	Q	D	-	-	-	-	K	-	-	-	-	V	-	-	S	L
		O/MAY/10/1994	-	-	-	Q	-	-	-	-	-	-	-	-	-	-	-	-	-	-	-	-	-	-	-	-	-	V	L	-	-	E	-	-	-	-	-	K	-	-	-	-	V	-	-	T	L
		O/TAI/6/1995	-	-	-	-	-	-	-	-	-	-	-	-	-	-	-	-	-	-	-	-	-	-	-	-	-	V	H	-	-	E	-	-	-	-	-	K	-	-	-	-	V	-	-	T	L
		O/TAI/2/2000/DQ164979	-	-	R	Q	-	-	-	-	-	-	-	-	-	-	-	-	-	-	-	-	-	-	-	-	-	V	H	-	-	E	-	-	-	-	-	K	-	-	-	-	V	-	-	T	L
	Bur-77	O/BUR/2/1977	-	-	-	-	-	-	-	-	-	-	-	-	-	-	-	-	-	-	-	-	-	-	-	-	-	V	H	-	-	E	-	-	-	-	-	K	-	-	-	-	V	-	-	L	L
		O/BUR/8/1978	-	-	-	-	-	-	-	-	-	-	-	-	-	-	-	-	-	-	-	-	-	-	-	-	-	V	H	-	-	E	-	-	-	-	-	K	-	-	-	-	V	-	-	L	L
		O/BUR/2/1978	-	-	-	-	-	-	-	-	-	-	-	-	-	-	-	-	-	-	-	-	-	-	-	-	-	V	H	-	-	E	-	-	-	-	-	K	-	-	-	-	V	-	-	L	W
		O/LAO/1/1982	-	-	-	-	-	I	-	-	-	-	-	-	-	-	-	-	-	-	-	-	-	-	-	-	-	V	H	-	-	E	-	-	-	-	-	K	-	-	-	-	V	-	-	L	L
		O/LAO/5/1982	-	-	-	-	-	I	-	-	-	-	-	-	-	-	-	-	-	-	-	-	-	-	-	-	-	V	H	-	-	E	-	-	-	-	-	K	-	-	-	-	V	-	-	L	L
		O/BUR/11/1978	-	-	-	-	-	-	-	-	-	-	-	-	-	-	-	-	-	-	-	-	-	-	-	-	-	V	H	-	-	E	-	-	-	-	-	K	-	-	-	-	V	-	-	L	L
	Tai-87	O/MAY/1/1992	-	-	-	-	-	-	-	-	-	-	-	-	-	-	-	-	-	-	-	-	-	-	-	-	-	V	H	-	-	E	-	-	-	-	-	K	-	-	-	-	V	-	-	S	L
		O/MAY/5/1992	-	-	-	-	-	-	-	-	-	-	-	-	-	-	-	-	-	-	-	-	-	-	-	-	-	V	H	-	-	E	-	-	-	-	-	K	-	-	-	-	V	-	-	S	L
		O/MAY/5/1993	-	-	A	-	-	-	-	-	-	-	-	-	-	-	-	-	-	-	-	-	-	-	-	-	-	V	H	-	-	E	-	-	-	-	-	K	-	-	-	-	-	-	-	S	L
		O/LAO/1/1987	-	-	-	-	-	-	-	-	-	-	-	-	-	-	-	-	-	-	-	-	-	-	-	-	-	V	H	-	-	E	-	-	-	-	-	K	-	-	-	-	V	-	-	S	L
		O/LAO/1/1988	-	-	-	-	-	-	-	-	-	-	-	-	-	-	-	-	-	-	-	-	-	-	-	-	-	V	H	-	-	E	-	-	-	-	-	K	-	-	-	-	V	-	-	S	L
		KY091288/O/TAI/189/1987	-	-	-	W	-	-	-	-	-	-	-	-	-	-	-	-	-	-	-	-	-	-	-	-	-	V	H	-	-	E	-	-	-	-	-	K	-	-	-	-	V	-	-	S	L
		HQ116222/O/MYA/5/2002	-	-	-	-	-	-	-	-	-	-	-	-	-	-	-	-	-	-	-	-	-	-	-	-	-	V	H	-	-	E	-	-	-	-	-	K	-	-	-	-	V	-	-	S	L
		O/CAM/1/1989	-	-	R	-	-	-	-	-	-	-	-	-	-	-	-	-	-	-	-	-	-	-	-	-	-	-	H	-	D	Q	-	-	-	-	-	K	-	-	-	-	V	-	-	S	L
		O/CAM/3/1992	-	-	S	-	-	-	-	-	-	-	-	-	-	-	-	-	-	-	-	-	-	-	-	-	-	-	H	-	D	Q	-	-	-	-	-	K	-	-	-	-	V	-	-	S	L
		O/TAI/1/1994	-	-	-	-	-	-	-	-	-	-	-	-	-	-	-	-	-	-	-	-	-	-	-	-	-	V	H	-	-	E	-	-	-	-	-	K	-	-	-	-	V	-	-	A	L
	Mya-98	SEA/AJ303536/TAI/4/1999	-	-	-	-	-	-	-	-	-	-	-	-	-	-	-	-	-	-	-	-	-	-	-	-	-	V	H	-	D	G	-	-	-	N	-	E	L	-	-	?	V	-	-	S	L
		SEA/AJ303521/MYA/1/1998	-	-	-	-	-	-	-	-	-	-	-	-	-	-	-	?	-	-	-	-	-	-	-	-	-	V	H	-	D	E	-	-	-	-	-	K	-	-	-	-	V	-	-	S	L
		HQ116240/O/TAI/4/2007	-	-	-	-	-	-	-	-	-	-	-	-	-	-	-	-	-	-	-	-	-	-	-	-	-	V	H	-	D	E	-	-	-	-	-	K	-	-	-	-	V	-	-	S	L
		HQ116241/O/TAI/5/2007	-	-	-	-	-	-	-	-	-	-	-	-	-	-	-	-	-	-	-	-	-	-	-	-	-	V	H	-	D	E	-	-	-	-	-	K	-	-	-	-	V	-	-	S	L
		JQ070302/O/HKN/6/2010	-	-	-	-	-	-	-	-	-	-	-	-	-	-	-	-	-	-	-	-	-	-	-	-	-	V	H	-	-	A	-	-	-	-	-	K	-	-	-	-	V	-	-	S	L
		JQ070301/O/HKN/1/2010	-	-	-	-	-	-	-	-	-	-	-	-	-	-	-	-	-	-	-	-	-	-	-	-	-	V	H	-	-	A	-	-	-	-	-	K	-	-	-	-	V	-	-	S	L
		HQ116209/O/MAY/5/2007	-	-	-	-	-	-	-	-	-	-	-	-	-	-	-	-	-	-	-	-	-	-	-	-	-	V	H	-	D	Q	-	-	-	-	-	K	-	-	-	-	V	-	-	S	W
		HQ116247/O/TAI/12/2007	-	-	-	-	-	-	-	-	-	-	-	-	-	-	-	-	-	-	-	-	-	-	-	-	-	V	H	-	D	Q	-	-	-	-	-	K	-	-	-	-	V	-	-	S	W
		HM055494/O/VN/HB139/2009	-	-	-	-	-	-	-	-	-	-	-	-	-	-	-	-	-	-	-	-	-	-	-	-	-	V	H	-	D	Q	-	-	-	-	-	K	-	-	-	-	V	-	-	S	W
		HM055498/O/VN/HB162/2009	-	-	-	-	-	I	-	-	-	-	-	-	-	-	-	-	-	-	-	-	-	-	-	-	-	V	H	-	D	Q	-	-	-	-	-	K	-	-	-	-	V	-	-	S	W
		HQ116289/O/VIT/6/2006	-	-	-	-	-	-	-	-	-	-	-	-	-	-	-	-	-	-	-	-	-	-	-	-	-	V	H	-	D	V	-	-	Y	-	-	K	-	-	-	-	V	-	-	S	L
	Cam-94	AJ294908/O/CAM/1/1998	-	-	-	-	-	-	-	-	-	-	-	-	-	-	-	-	-	-	-	-	-	-	-	-	-	-	H	-	-	E	-	-	-	-	-	K	-	-	-	-	Q	-	-	L	L
		AJ294909/O/CAM/2/1998	-	-	-	-	-	-	-	-	-	-	-	-	-	-	-	-	-	-	-	-	-	-	-	-	-	-	H	-	-	E	-	-	-	-	-	K	-	-	-	-	Q	-	-	L	L
		AJ294910/O/CAM/3/1998	-	-	-	-	-	-	-	-	-	-	-	-	-	-	-	-	-	-	-	-	-	-	-	-	-	-	H	-	-	E	-	-	-	-	-	K	-	-	-	-	Q	-	-	L	L
		SA/AJ296328/VIT/7/1997	-	-	-	Q	-	-	-	-	-	-	-	-	-	-	-	-	-	-	-	-	-	-	-	-	-	-	H	-	D	E	-	-	-	-	-	K	-	-	-	-	E	-	-	L	L
		EU667442/O/LAO/2/1999	-	-	-	-	-	-	-	-	-	-	-	-	-	-	-	-	-	-	-	-	-	-	-	-	-	-	H	-	H	E	-	-	-	-	-	K	-	-	-	-	E	R	-	L	L
		EU667440/O/LAO/1/1998	-	-	-	-	-	-	-	-	-	-	-	-	-	-	-	-	-	-	-	-	-	-	-	-	-	-	H	-	N	E	-	-	-	-	-	K	-	-	-	-	E	R	-	L	L
ME-SA	PanAsia-2	ME-SA/PanAsia-2/ANT-10/KY091282/O/IRN/88/2009	-	-	-	-	-	N	-	-	-	-	-	-	-	-	-	-	-	-	-	-	-	-	-	-	-	-	H	-	N	E	-	-	-	-	-	K	-	-	-	-	V	-	-	L	L
		PA2/KJ831726/PAK/1/2010	-	-	-	-	-	N	-	-	-	-	-	-	-	-	-	-	-	-	-	-	-	-	-	-	-	-	H	-	N	E	-	-	-	-	-	K	-	-	-	-	V	-	-	-	-
		PA2/KR149715/O/IRN/31/2010	-	-	-	-	-	N	-	-	-	-	-	-	-	-	-	-	-	-	-	-	-	-	-	-	-	-	H	-	N	E	-	-	-	-	-	K	-	-	-	-	V	-	-	L	L
		PA2/KR149717/O/UAE/2/2010	-	-	-	-	-	N	-	-	-	-	-	-	-	-	-	-	-	-	-	-	-	-	-	-	-	-	H	-	N	E	-	-	-	-	-	K	-	-	-	-	V	-	-	L	L
		ME-SA/PanAsia-2/KY091281/O/IRN/8/2005	-	-	-	-	-	-	-	-	-	-	-	-	-	-	-	-	-	-	-	-	-	-	-	-	-	-	H	-	-	E	-	-	-	-	-	K	-	-	-	-	V	-	-	L	L
		ME-SA/PanAsia-2/FAR-09/KY091284/O/IRN/31/2009	-	-	-	Q	-	-	-	-	-	-	-	-	-	-	-	-	-	-	-	-	-	-	-	-	-	-	H	-	A	E	-	-	-	-	-	K	-	-	-	-	V	-	-	L	L
		ME-SA/PanAsia-2/BAL-09/KY091283/O/IRN/18/2010	-	-	-	Q	-	-	-	-	-	-	-	-	-	-	-	-	-	-	-	-	-	-	-	-	-	-	H	-	-	E	-	-	-	-	-	K	-	-	-	-	-	-	-	L	L
		ME-SA/PanAsia-2/PUN-10/KY091285/O/PAK/16/2010	-	-	-	-	-	-	-	-	-	-	-	-	-	-	-	-	-	-	-	-	-	-	-	-	-	-	H	-	N	E	-	-	-	-	-	K	-	-	-	-	V	-	-	L	L
		PA2/HQ439233/O/KAB/AFG/L643/2009	-	-	-	-	-	-	-	-	-	-	-	-	-	-	-	-	-	-	-	-	-	-	-	-	-	-	H	-	-	E	-	-	-	-	-	K	-	-	-	-	V	-	-	L	L
		PA2/HQ439234/O/BAG/AFG/L1494/2009	-	-	-	-	-	-	-	-	-	-	-	-	-	-	-	-	-	-	-	-	-	-	-	-	-	-	H	-	-	E	-	-	-	-	-	K	-	-	-	-	V	-	-	I	L
		PA2/HQ439232/O/KUN/AFG/L630/2009	-	-	-	-	-	-	-	-	-	-	-	-	-	-	-	-	-	-	-	-	-	-	-	-	-	V	H	-	-	E	-	-	-	-	-	K	-	-	-	-	V	-	-	L	L
	PanAsia	ME-SA/PanAsia/AJ539141/O/UKG/35/2001	-	-	-	-	-	-	-	-	-	-	-	-	-	-	-	-	-	-	-	-	-	-	-	-	-	-	H	-	-	E	-	-	-	-	-	K	-	-	-	-	V	-	-	L	L
		MESA/AJ303499/IRQ/30/2000	-	-	-	-	-	-	-	-	-	-	-	-	-	-	-	-	-	-	-	-	-	-	-	-	-	-	H	-	-	E	-	-	-	-	-	K	-	-	-	-	V	-	-	L	L
		PA/HQ116172/O/CAM/4/2006	-	-	-	-	-	-	-	-	-	-	-	-	-	-	-	-	-	-	-	-	-	-	-	-	-	-	H	-	-	E	-	-	-	-	-	K	-	-	-	-	V	-	-	L	L
		PA/HQ116173/O/CAM/5/2006	-	-	-	-	-	-	-	-	-	-	-	-	-	-	-	-	-	-	-	-	-	-	-	-	-	-	H	-	-	E	-	-	-	-	-	K	-	-	-	-	V	-	-	L	L
		PA/HQ116171/O/CAM/1/2004	-	-	-	-	-	-	-	-	-	-	-	-	-	-	-	-	-	-	-	-	-	-	-	-	-	-	H	-	-	E	-	-	-	-	-	K	-	-	-	-	V	-	-	F	L
		PA/HQ116174/O/CAM/1/2008	-	-	-	-	-	-	-	-	-	-	-	-	-	-	-	-	-	-	-	-	-	-	-	-	-	-	H	-	-	E	-	-	-	-	-	K	-	-	-	-	E	-	-	V	L
		ME-SA/PanAsia/EU667451/O/LAO/2/2003	-	-	-	-	-	-	-	-	-	-	-	-	-	-	-	-	-	-	-	-	-	-	-	-	-	-	H	-	-	E	-	-	-	-	-	K	-	-	-	-	V	-	-	L	L
	Irn-2001	Irn2001/DQ165062/O/PAK/15/2002	-	-	-	-	-	-	-	-	-	-	-	-	-	-	-	-	-	-	-	-	-	-	-	-	-	-	H	-	-	E	-	-	-	-	-	K	-	-	-	-	V	-	-	L	L
		Irn2001/DQ165063/O/PAK/16/2002	-	-	-	-	-	-	-	-	-	-	-	-	-	-	-	-	-	-	-	-	-	-	-	-	-	-	H	-	-	E	-	-	-	-	-	K	-	-	-	-	V	-	-	L	L
		Irn2001/DQ165064/O/PAK/18/2002	-	-	-	-	-	-	-	-	-	-	-	-	-	-	-	-	-	-	-	-	-	-	-	-	-	-	H	-	-	E	-	-	-	-	-	K	-	-	-	-	V	-	-	L	L
		AJ296325/TUR/1/1996	-	-	-	-	-	-	-	-	-	-	-	-	-	-	-	-	-	-	-	-	-	-	-	-	-	-	H	-	-	E	-	-	-	-	?	K	-	?	-	-	-	-	-	-	-
	Ind-2001	ME-SA/Ind-2001a/DQ164904/O/KUW/3/1997	-	-	-	-	-	-	-	-	-	-	-	-	-	-	-	-	-	-	-	-	-	-	-	-	-	-	H	-	-	E	-	-	-	-	-	K	-	-	-	-	V	-	-	L	L
		ME-SA/Ind-2001b/DQ164941/O/OMN/7/2001	-	-	-	-	-	-	-	-	-	-	-	-	-	-	-	-	-	-	-	-	-	-	-	-	-	-	H	-	N	E	-	-	-	-	-	K	-	-	-	-	V	-	-	L	L
		ME-SA/Ind-2001c/KM921876/O/UAE/4/2008	-	-	-	-	-	-	-	-	-	-	-	-	-	-	-	-	-	-	-	-	-	-	-	-	-	-	H	-	-	E	-	-	-	-	-	K	-	-	-	-	V	-	-	L	L
		MF947475/SRL/2/2014	-	-	-	-	-	-	-	-	-	-	-	-	-	-	-	-	-	-	-	-	-	-	-	-	T	-	H	-	-	E	-	-	-	-	-	K	-	-	-	-	V	-	-	L	L
		MF947474/SRL/1/2014	-	-	-	-	-	-	-	-	-	-	-	-	-	-	-	-	-	-	-	-	-	-	-	-	-	-	H	-	-	E	-	-	-	-	-	K	-	-	-	-	V	-	-	L	L
		MF947473/SRL/2/2013	-	-	-	-	-	-	-	-	-	-	-	-	-	-	-	-	-	-	-	-	-	-	-	-	-	-	H	-	-	E	T	-	-	-	-	K	-	-	-	-	V	-	-	L	L
		ME-SA/Ind-2001d/KM921814/O/BHU/3/2009	-	-	-	-	-	-	-	-	-	-	-	-	-	-	-	-	-	-	-	-	-	-	-	-	-	-	H	-	-	Q	-	-	-	-	-	K	-	-	-	-	V	-	-	L	L
	Srl-97	MF947468/SRL/13/2010	-	-	-	-	-	-	-	-	-	-	-	-	-	-	-	-	-	-	-	-	-	-	-	-	-	-	H	-	-	G	-	-	-	-	-	K	-	-	-	-	V	-	-	L	L
		MF947467/SRL/9/2010	-	-	-	-	-	-	-	-	-	-	-	-	-	-	-	-	-	-	-	-	-	-	-	-	-	-	H	-	-	E	-	-	-	-	-	K	-	-	-	-	V	-	-	L	L
		KJ831703/SRL/1/2009	-	-	-	-	-	-	-	-	-	-	-	-	-	-	-	-	-	-	-	-	-	-	-	-	-	-	H	-	-	E	-	-	-	-	-	K	-	-	-	-	V	-	-	-	-
		MF947463/SRL/1/2009	-	-	-	-	-	-	-	-	-	-	-	-	-	-	-	-	-	-	-	-	-	-	-	-	-	-	H	-	-	E	-	-	-	-	-	K	-	-	-	-	V	-	-	L	L
		MF947455/SRL/4/1999	-	-	-	-	-	-	-	-	-	-	-	-	-	-	-	-	-	-	-	-	-	-	-	-	-	-	H	-	N	E	-	-	-	-	-	K	-	-	-	-	V	-	-	L	L
		MF947453/SRL/1/1999	-	-	-	-	-	-	-	-	-	-	-	-	-	-	-	-	-	-	-	-	-	-	-	-	-	-	H	-	N	E	-	-	-	-	-	K	-	-	-	-	V	-	-	L	L
		MF947454/SRL/3/1999	-	-	-	-	-	-	-	-	-	-	-	-	-	-	-	-	-	-	-	-	-	-	-	-	-	-	H	-	N	E	-	-	-	-	-	K	-	-	-	-	V	-	-	L	L
		AJ303531/SRL/2/1997	-	-	-	-	-	-	-	-	-	-	-	-	-	-	-	-	-	-	-	-	-	-	-	-	-	-	H	-	N	E	-	-	-	-	-	K	-	-	-	-	V	-	-	L	L
		ME-SA/Srl-97/AJ303531/O/SRL/2/1997	-	-	-	-	-	-	-	-	-	-	-	-	-	-	-	-	-	-	-	-	-	-	-	-	-	-	H	-	N	E	-	-	-	-	-	K	-	-	-	-	V	-	-	L	L
		MF947470/SRL/4/2011	-	-	-	-	-	-	-	-	-	-	-	-	-	-	-	-	-	-	-	-	-	-	-	-	-	-	H	-	-	E	-	-	-	-	-	K	-	-	-	-	V	-	-	L	L
	Pak-98	Pak98/DQ165068/O/PAK/16/2003	-	-	-	-	-	-	-	-	-	-	-	-	-	-	-	-	-	-	-	-	-	-	-	-	-	-	H	-	N	G	-	-	-	-	-	K	-	-	-	-	M	-	-	L	F
		Pak98/DQ165070/O/PAK/73/2003	-	-	-	-	-	-	-	-	-	-	-	-	-	-	-	-	-	-	-	-	-	-	-	-	-	-	H	-	N	G	-	-	-	-	-	K	-	-	-	-	M	-	-	L	F
		Pak98/DQ165069/O/PAK/17/2003	-	-	-	-	-	-	-	-	-	-	-	-	-	-	-	-	-	-	-	-	-	-	-	-	-	-	H	-	N	G	D	-	-	-	-	K	-	-	-	-	M	-	-	L	L
	Unnamed	ME-SA/unnamed/AF204276/O/IND/R2/1975	-	-	-	-	-	-	-	-	-	-	-	-	-	-	-	-	-	-	-	-	-	-	-	-	-	-	H	-	N	E	-	-	-	-	-	K	-	-	-	-	V	-	-	L	L
		ME-SA/unnamed/AF292107/O/IND/53/1979	-	-	-	-	-	I	-	-	-	-	-	-	-	-	-	-	-	-	-	-	-	-	-	-	-	-	H	-	N	E	-	-	-	-	-	K	-	-	-	-	V	-	-	L	L
	Tur-69	EU553844/O/ISR/1/1992	-	-	-	-	-	-	-	-	-	-	-	-	-	-	-	-	-	-	-	-	-	-	-	-	-	-	H	?	-	E	-	-	-	-	-	K	-	-	-	-	-	-	-	L	L
		EU553845/O/ITL/1/1993	-	-	-	-	-	-	-	-	-	-	-	-	-	-	-	-	-	-	-	-	-	-	-	-	-	-	H	-	-	E	-	-	-	-	-	K	-	-	-	-	-	-	-	L	L
		EU553846/O/OMN/3/1991	-	-	-	-	-	-	-	-	-	-	-	-	-	-	-	-	-	-	-	-	-	-	-	-	-	-	H	-	-	E	-	-	-	-	-	K	-	-	-	-	-	-	-	L	L
		O/SRL/1/1991	-	-	-	-	-	-	-	-	-	-	-	-	-	-	-	-	-	-	-	-	-	-	-	-	-	-	H	-	-	E	-	-	-	-	-	K	-	-	-	-	V	-	-	L	L

Topotype	Liniage	Isolate	170	171	172	173	174	175	176	177	178	179	180	181	182	183	184	185	186	187	188	189	190	191	192	193	194	195	196	197	198	199	200	201	202	203	204	205	206	207	208	209	210	211	212	213	214
Reference Sequnce		NC 004004 ref seq	K	A	T	R	V	T	E	L	L	Y	R	M	K	R	A	E	T	Y	C	P	R	P	L	L	A	I	Q	P	S	D	A	R	H	K	Q	R	I	V	A	P	A	K	Q	-	-
		ME-SA/unnamed/AY593823/O1/Manisa/TUR/1969	-	-	-	-	-	-	-	-	-	-	-	-	-	-	-	-	-	-	-	-	-	-	-	-	-	-	H	-	D	Q	-	-	-	-	-	K	-	-	-	-	V	-	-	L	L
WCSA-1	Srl-77	O/SRL/7/88/1987	-	-	A	-	-	-	-	-	-	-	-	-	-	-	-	-	-	-	-	-	-	-	-	-	-	-	H	-	D	E	-	-	-	-	-	K	-	-	-	-	S	-	-	L	L
		O/SRL/5/1987	-	-	A	-	-	-	-	-	-	-	-	-	-	-	-	-	-	-	-	-	-	-	-	-	-	-	H	-	D	E	-	-	-	-	-	K	-	-	-	-	S	-	-	L	L
		O/SRL/4/88/1987	-	-	A	-	-	-	-	-	-	-	-	-	-	-	-	-	-	-	-	-	-	-	-	-	-	-	H	-	D	E	-	-	-	-	-	K	-	-	-	-	S	-	-	L	L
		O/SRL/3/88/1987	-	-	A	-	-	-	-	-	-	-	-	-	-	-	-	-	-	-	-	-	-	-	-	-	-	-	H	-	D	E	-	-	-	-	-	K	-	-	-	-	S	-	-	L	L
		O/SRL/6/88/1987	-	-	A	-	-	-	-	-	-	-	-	-	-	-	-	-	-	-	-	-	-	-	-	-	-	-	H	-	D	E	-	-	-	-	-	K	-	-	-	-	S	-	-	L	L
		O/SRL/9/88/1987	-	-	A	-	-	-	-	-	-	-	-	-	-	-	-	-	-	-	-	-	-	-	-	-	-	-	H	-	D	E	-	-	-	-	-	K	-	-	-	-	S	-	-	L	L
		O/SRL/2/88/1987	-	-	A	-	-	-	-	-	-	-	-	-	-	-	-	-	-	-	-	-	-	-	-	-	-	-	H	-	D	E	-	-	-	-	-	K	-	-	-	-	S	-	-	L	L
		O/SRL/1/88/1987	-	-	A	-	-	-	-	-	-	-	-	-	-	-	-	-	-	-	-	-	-	-	-	-	-	-	H	-	D	E	-	-	-	-	-	K	-	-	-	-	S	-	-	L	L
		O/SRL/8/88/1987	-	-	A	-	-	-	-	-	-	-	-	-	-	-	-	-	-	-	-	-	-	-	-	-	-	-	H	-	D	E	-	-	-	-	-	K	-	-	-	-	P	-	-	L	L
		O/SRL/10/1988	-	-	A	-	-	-	-	-	-	-	-	-	-	-	-	-	-	-	-	-	-	-	-	-	-	-	H	-	D	E	-	-	-	-	-	K	-	-	-	-	S	-	-	L	L
		O/SRL/1/1997	-	-	E	-	-	-	-	-	-	-	-	-	-	-	-	-	-	-	-	-	-	-	-	-	-	V	H	-	N	E	-	-	-	-	-	K	-	-	-	-	S	R	-	L	L
		O/SRL/1/78/1977	-	-	-	-	-	-	-	-	-	-	-	-	-	-	-	-	-	-	-	-	-	-	-	-	-	-	H	-	-	E	-	-	-	-	-	K	-	-	-	-	T	-	-	L	L
		O/SRL/3/1978	-	-	-	-	-	-	-	-	-	-	-	-	-	-	-	-	-	-	-	-	-	-	-	-	-	-	H	-	-	E	-	-	-	-	-	K	-	-	-	-	T	-	-	L	L
		O/SRL/2/1978	-	-	-	-	-	-	-	-	-	-	-	-	-	-	-	-	-	-	-	-	-	-	-	-	-	-	H	-	-	E	-	-	-	-	-	K	-	-	-	-	T	-	-	L	L
		O/SRL/87/VS/1987	-	-	-	-	-	-	-	-	-	-	-	-	-	-	-	-	-	-	-	-	-	-	-	-	-	-	H	-	-	E	-	-	-	-	-	K	-	-	-	-	-	-	-	L	L
		O/SRL/3/1987	-	-	-	-	-	-	-	-	-	-	-	-	-	-	-	-	-	-	-	-	-	-	-	-	-	V	H	-	-	E	-	-	-	-	-	K	-	-	-	-	-	-	-	L	L
	Syr-62	AJ004679/O/1492/Russia/USSR/88/1988	-	-	D	-	-	-	-	-	-	-	-	-	-	-	-	-	-	-	-	-	-	-	-	-	-	-	H	-	-	E	-	-	-	-	-	K	-	-	-	-	-	-	-	L	L
		O/SYR/18/1962	-	-	D	-	-	-	-	-	-	-	-	-	-	-	-	-	-	-	-	-	-	-	-	-	-	-	H	-	-	E	-	-	-	-	-	K	-	-	-	-	-	-	-	L	L
		AY593834/O/Rey/IRN/66/1966	-	-	V	-	-	-	-	-	-	-	-	-	-	-	-	-	-	-	-	-	-	-	-	-	-	-	H	-	D	E	-	-	-	-	-	K	-	-	-	-	T	-	-	L	L
		AJ004663/O/738/Tajikistan/USSR/74/1974	-	-	E	-	-	-	-	-	-	-	-	-	-	-	-	-	-	-	-	-	-	-	-	-	-	-	H	-	-	E	-	-	-	-	-	K	-	-	-	-	E	-	-	L	L
EA-3		EA-3/unnamed/FJ798109/O/ETH/3/2004	-	-	-	Q	-	-	-	-	-	-	-	-	-	-	-	-	-	-	-	-	-	-	-	-	-	-	H	-	-	E	-	-	-	-	-	K	-	-	-	-	V	-	-	L	L
		EA-3/unnamed/FJ798137/O/ETH/1/2007	-	-	-	-	-	-	-	-	-	-	-	-	-	-	-	-	-	-	-	-	-	-	-	-	-	-	H	-	-	E	-	-	-	-	-	K	-	-	-	-	V	-	-	L	L
		EA-3/unnamed/DQ165075/O/SUD/2/1986	-	-	-	-	-	-	-	-	-	-	-	-	-	-	-	-	-	-	-	-	-	-	-	-	-	-	H	-	T	E	-	-	-	-	-	K	-	-	-	-	V	-	-	L	L
		EA-3/unnamed/FJ798127/O/ETH/2/2006	-	-	-	-	-	V	-	-	-	-	-	-	-	-	-	-	-	-	-	-	-	-	-	-	-	-	H	-	-	E	-	-	-	-	-	K	-	-	-	-	E	-	-	L	L
EA-1		EA/AJ303511/KEN/83/1979	-	-	-	-	-	-	-	-	-	-	-	-	-	-	-	-	-	-	-	-	-	-	-	-	-	T	H	-	-	E	-	-	-	-	-	-	-	-	-	-	-	-	-	?	L
		EA/AJ303514/KEN/2/95/K10/1995	-	-	-	-	-	I	-	-	-	-	-	-	-	-	-	-	-	-	-	-	-	-	-	-	-	T	H	-	-	E	-	-	-	-	-	-	-	I	-	-	-	-	-	L	L
		EA/AJ296327/UGA/5/1996	-	-	-	-	-	-	-	-	-	-	-	-	-	-	-	-	-	-	-	-	-	-	-	-	-	-	H	-	-	E	-	-	-	-	-	K	-	-	-	-	-	R	-	L	L
EA-2		EA-2/unnamed/DQ165073/O/KEN/5/2002	-	-	-	-	-	-	-	-	-	-	-	-	-	-	-	-	-	-	-	-	-	-	-	-	-	-	H	-	-	E	-	-	-	-	-	K	-	-	-	-	V	-	-	L	L
		EA-2/unnamed/DQ165077/O/UGA/3/2002	-	-	-	Q	-	-	-	-	-	-	-	-	-	-	-	-	-	-	-	-	-	-	-	-	-	-	H	-	-	-	-	-	-	-	-	K	-	-	-	-	V	-	-	L	L
		EA-2/unnamed/KF561679/O/TAN/2/2004	-	-	-	-	-	-	-	-	-	-	-	-	-	-	-	-	-	-	-	-	-	-	-	-	-	-	H	-	-	-	-	-	-	-	-	K	-	-	-	-	-	-	-	L	L
		EA-2/unnamed/DQ165074/O/MAL/1/1998	-	-	-	-	-	-	-	-	-	-	-	-	-	-	-	-	-	-	-	-	-	-	-	-	-	-	H	-	-	E	-	-	-	-	-	K	-	-	-	-	V	-	-	L	L
EA-4		EA-4/unnamed/FJ798141/O/ETH/58/2005	-	-	-	-	-	I	-	-	-	-	-	-	-	-	-	-	-	-	-	-	-	-	-	-	-	V	H	-	-	E	-	-	-	-	-	K	-	-	-	-	V	-	-	L	L
		EA-4/unnamed/HM211075/O/UGA/17/1998	-	-	-	-	-	-	-	-	-	-	-	-	-	-	-	-	-	-	-	-	-	-	-	-	-	V	H	-	T	E	-	-	-	-	-	K	-	-	-	-	V	-	-	L	L
WA		WA/AJ303481/ALG/1/1999	-	-	-	-	-	-	-	-	-	-	-	-	-	-	-	-	-	-	-	-	-	-	-	-	?	F	H	-	-	E	-	G	-	-	?	E	?	L	-	-	V	-	-	L	L
		WA/AJ303485/CIV/8/1999	-	-	-	-	-	-	-	-	-	-	-	-	-	-	-	-	-	-	-	-	-	-	-	-	-	F	H	-	-	E	-	-	-	-	-	K	-	-	-	-	-	-	-	L	L
		WA/AJ303488/GHA/5/1993	-	-	-	-	-	-	-	-	-	-	-	-	-	-	-	-	-	-	-	-	-	-	-	-	-	V	H	-	-	E	T	-	-	-	-	K	-	-	-	-	M	-	-	L	L
Cathay		CATHAY/unnamed/AF308157/O/Yunlin/TAW/1997	-	-	-	-	-	-	-	-	-	-	-	-	-	-	-	-	-	-	-	-	-	-	-	-	-	-	-	-	-	-	-	-	-	-	-	-	-	-	-	-	-	-	-	L	L
		Cathey/AJ296321/TAW/81/1997	-	-	-	-	-	-	-	-	-	-	-	-	-	-	-	-	-	-	-	-	-	-	-	-	-	-	-	-	-	-	-	-	-	-	-	-	-	-	-	-	-	-	-	L	L
		1997/AJ296322/TAW/83/1997	-	-	I	-	-	-	-	-	-	-	-	-	-	-	-	-	-	-	-	-	-	-	-	-	-	?	-	-	-	-	-	-	-	-	-	-	-	-	-	-	-	-	-	?	L
		AJ294923/O/HKN/16/1996	-	-	-	-	-	-	-	-	-	-	-	-	-	-	-	-	A	-	-	-	-	-	-	-	-	-	-	-	-	-	-	-	-	-	-	K	-	-	-	-	-	R	-	L	?
		Cathey/AJ004680/O/1685/Moscow/Russia/1995	-	-	-	-	-	-	-	-	-	-	-	-	-	-	-	-	-	-	-	-	-	-	-	-	-	-	-	-	-	-	-	-	-	-	-	K	-	-	-	?	-	-	-	L	L
		Cathey/AJ294926/O/PHI/7/1996	-	-	-	-	-	-	-	-	-	-	-	-	-	-	-	-	-	-	-	-	-	-	-	-	-	-	-	-	-	-	-	-	R	-	-	K	-	-	-	-	-	-	-	L	L
		CATHAY/unnamed/AJ294919/O/HKN/6/1983	-	-	-	-	-	-	-	-	-	-	-	-	-	-	-	-	-	-	-	-	S	-	-	-	-	-	-	-	-	-	-	-	-	-	-	K	-	-	-	-	-	-	-	L	L
		Cathey/AJ131468/GD/China/1986	-	-	-	-	-	-	-	-	-	-	-	-	-	-	-	-	-	-	-	-	-	-	-	-	-	-	-	-	-	-	-	-	-	-	-	K	-	-	-	-	-	-	-	L	L
		Cathey/AJ294917/O/HKN/14/1982	-	-	-	-	-	-	-	-	-	-	-	-	-	-	-	-	-	-	-	-	-	-	-	-	-	-	-	-	-	-	-	-	-	-	-	K	-	-	-	-	-	-	-	L	L
		CATHAY/unnamed/AJ294911/O/HKN/21/1970	-	-	-	-	-	-	-	-	-	-	-	-	-	-	-	-	-	-	-	-	-	-	-	-	-	-	-	-	-	-	-	-	-	-	-	K	-	-	-	-	-	-	-	L	L
EURO-SA		EURO-SA/O1/AY593815/O1/BFS 1860/UK/1867	-	-	-	-	-	-	-	-	-	-	-	-	-	-	-	-	-	-	-	-	-	-	-	-	-	-	H	-	T	E	-	-	-	-	-	K	-	-	-	-	V	-	-	T	L
		EuroSA/K01201/O1Campos/Brazil/1971	-	-	-	-	-	-	-	-	-	-	-	-	-	-	-	-	-	-	-	-	-	-	-	-	-	-	H	-	T	E	-	-	-	-	-	K	-	-	-	-	V	-	-	T	L
		EuroSA/X00871/O1/Kaufbeuren/FRG/1966	-	-	-	-	-	-	-	-	-	-	-	-	-	-	-	-	-	-	-	-	-	-	-	-	-	-	H	-	T	E	-	-	-	-	-	K	-	-	-	-	V	-	-	T	L
		EuroSA/Z21862/O/Yrigoyen/ARG/1982	-	-	-	-	-	-	-	-	-	-	-	-	-	-	-	-	-	-	-	-	-	-	-	-	-	-	H	-	T	-	-	-	-	-	R	K	-	-	-	-	V	-	R	T	L
		EuroSA/M89900/O1/Caseros/ARG/1967	-	-	S	-	-	-	-	-	-	-	-	-	-	-	-	-	-	-	-	-	-	-	-	-	-	-	H	-	T	-	-	-	-	-	-	K	-	-	-	A	V	-	-	T	L
		Eurosa/M58601/O1/Lombardy/ITL/1946	R	-	-	-	-	-	-	-	-	-	-	-	-	-	-	-	-	-	-	-	-	-	-	-	-	-	H	-	T	E	-	-	-	-	-	K	-	-	-	-	V	-	-	T	L
		EuroSA/M55287/O2/Brescia/ITL/1947	R	-	-	-	-	-	-	-	-	-	-	-	-	-	-	-	-	-	-	-	-	-	-	-	-	-	H	-	T	E	-	-	-	-	-	K	-	-	-	-	-	-	-	T	L
		EuroSA/AJ004645/O3/Venezuela/1951	-	-	-	-	-	-	-	-	-	-	-	-	-	-	-	-	-	-	-	-	-	-	-	-	-	-	H	-	T	E	-	-	-	-	-	K	-	-	-	-	V	-	-	T	L
		EURO-SA/unnamed/DQ834727/O/Corrientes/ARG/2006	-	-	-	-	-	-	-	-	-	-	-	-	-	-	-	-	-	-	-	-	-	-	-	-	-	V	H	-	-	E	-	-	-	-	-	K	-	-	-	-	V	-	-	S	L
CEY-1		O/CEY/1/71/1962	-	-	-	Q	-	-	-	-	-	-	-	-	-	-	-	-	V	-	-	-	-	-	-	-	-	-	-	-	-	E	-	-	-	-	-	K	-	-	-	-	V	-	-	A	L
		O/SRL/1/76/1962	-	-	-	Q	-	-	-	-	-	-	-	-	-	-	-	-	V	-	-	-	-	-	-	-	-	-	-	-	-	E	-	-	-	-	-	K	-	-	-	-	V	-	-	A	L
		O/SRL/1/1982	-	-	-	Q	-	S	-	-	-	-	-	-	-	-	-	-	V	-	-	-	-	-	-	-	-	V	L	-	-	E	-	-	-	-	-	K	-	-	-	-	-	-	-	V	L
ISA-2		ISA2/AJ303501/ISA/1/1974	-	-	-	-	-	V	-	-	-	-	-	-	-	-	-	-	A	-	-	-	-	-	-	-	-	-	-	-	-	E	-	-	-	-	-	K	-	-	-	-	V	-	-	T	L
		ISA2/AJ303509/JAV/5/1972	-	-	-	-	L	V	-	-	-	-	-	-	-	-	-	-	A	-	-	-	-	-	-	-	-	-	-	-	-	E	-	-	-	-	-	K	-	-	-	-	V	-	-	T	L
ISA-1		ISA1/AJ303503/ISA/8/1983	-	-	-	-	-	I	-	-	-	-	-	I	-	-	-	-	-	-	-	-	-	-	-	-	-	-	H	-	-	E	-	-	-	-	-	K	-	-	-	-	T	-	-	T	L
		ISA1/AJ303502/ISA/9/1974	-	-	-	-	-	-	-	-	-	-	-	I	-	-	-	-	-	-	-	-	-	-	-	-	-	-	H	-	T	E	-	-	-	-	-	K	-	-	-	-	T	-	-	T	L
		ISA1/AJ303500/ISA/1/1962	-	-	-	-	-	-	-	-	-	-	-	I	-	-	-	-	-	-	-	-	-	-	-	-	-	-	R	-	-	E	-	-	-	-	-	K	-	-	-	-	V	-	-	T	L
